# Machine Learning Guides Peptide Nucleic Acid Flow Synthesis and Sequence Design

**DOI:** 10.1002/advs.202201988

**Published:** 2022-10-21

**Authors:** Chengxi Li, Genwei Zhang, Somesh Mohapatra, Alex J. Callahan, Andrei Loas, Rafael Gómez‐Bombarelli, Bradley L. Pentelute

**Affiliations:** ^1^ Department of Chemistry Massachusetts Institute of Technology 77 Massachusetts Avenue Cambridge MA 02139 USA; ^2^ College of Chemical and Biological Engineering Zhejiang University No.866 Yuhangtang Road Hangzhou Zhejiang 310030 P. R. China; ^3^ ZJU‐Hangzhou Global Scientific and Technological Innovation Center No.733 Jianshe San Road, Xiaoshan District Hangzhou Zhejiang 311200 P. R. China; ^4^ Department of Materials Science and Engineering Massachusetts Institute of Technology 77 Massachusetts Avenue Cambridge MA 02139 USA; ^5^ The Koch Institute for Integrative Cancer Research Massachusetts Institute of Technology 500 Main Street Cambridge MA 02142 USA; ^6^ Center for Environmental Health Sciences Massachusetts Institute of Technology 77 Massachusetts Avenue Cambridge MA 02139 USA; ^7^ Broad Institute of MIT and Harvard 415 Main Street Cambridge MA 02142 USA

**Keywords:** automated synthesis, drug design, machine learning, peptide nucleic acid, yield prediction

## Abstract

Peptide nucleic acids (PNAs) are potential antisense therapies for genetic, acquired, and viral diseases. Efficiently selecting candidate PNA sequences for synthesis and evaluation from a genome containing hundreds to thousands of options can be challenging. To facilitate this process, this work leverages machine learning (ML) algorithms and automated synthesis technology to predict PNA synthesis efficiency and guide rational PNA sequence design. The training data is collected from individual fluorenylmethyloxycarbonyl (Fmoc) deprotection reactions performed on a fully automated PNA synthesizer. The optimized ML model allows for 93% prediction accuracy and 0.97 Pearson's *r*. The predicted synthesis scores are validated to be correlated with the experimental high‐performance liquid chromatography (HPLC) crude purities (correlation coefficient *R* ^2^ = 0.95). Furthermore, a general applicability of ML is demonstrated through designing synthetically accessible antisense PNA sequences from 102 315 predicted candidates targeting exon 44 of the human dystrophin gene, SARS‐CoV‐2, HIV, as well as selected genes associated with cardiovascular diseases, type II diabetes, and various cancers. Collectively, ML provides an accurate prediction of PNA synthesis quality and serves as a useful computational tool for informing PNA sequence design.

## Graphical abstract

This work reports a highly effective machine learning (ML) model to predict peptide nucleic acid (PNA) synthesis efficiency. The optimized algorithm is applied to guide rational PNA sequence design for 18 different disease targets.

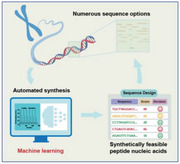

## 1 Introduction

In the past 5 years, antisense oligonucleotide (ASO) based drug development resulted in five Food and Drug Administration approved drugs, i.e., Eteplirsen,^[^ ^1^ ^]^ Golodirsen,^[^ ^2^ ^]^ Casimersen,^[^ ^3^ ^]^ Viltepso^[^ ^4^ ^]^ (based on phosphorodiamidate morpholino oligomers, PMOs), and Spinraza^[^ ^5^ ^]^ (based on 2′‐*O*‐methoxyethyl‐phosphorothioate). Backbone modifications increase the therapeutic potential of ASO‐based drugs due to improved pharmacokinetic and pharmacodynamic profiles. By assembling a charge‐neutral ASO, peptide nucleic acid (PNA) based chemistry is also gaining popularity for developing gene‐specific therapies.^[^ ^6^ ^]^ The amide‐based backbone of PNAs offers unique physicochemical properties including enhanced chemical, thermal, and enzymatic stability, as well as high hybridization affinity and specificity with DNA and RNA.^[^ ^7^ ^]^

To evaluate biologically active PNA sequences for a given indication, the existing approach is to screen a small PNA library that typically contains up to dozens of candidates, each with a length of about 20 bases. There typically are hundreds to thousands of sequence design options available when targeting a specific gene or genome. For example, the genome of severe acute respiratory syndrome coronavirus 2 (SARS‐CoV‐2) contains nearly 30 000 bases,^[^ ^8^ ^]^ raising a selection challenge when designing anti‐SARS‐CoV‐2 sequences. Therefore, it is crucial to select “high value” PNA sequences from the multitude of available options to minimize costs and workload in the development process. In addition, sequence‐dependent coupling efficiency should also be considered for each variant produced. The availability of routine computational algorithms, such as those enabled by machine learning (ML) to predict the efficiency of PNA synthesis, would represent a major step forward in improving overall PNA sequence design. To achieve this goal, a large high quality dataset and reliable training methods are essential.

In chemical synthesis, access to high quality, interpretable, and standardized collections of data suitable for ML remains limited.^[^ ^9^ ^]^ The data from published literature are usually collected using different reaction conditions and setups, and the reported results often exist in different formats.^[^ ^9a^ ^]^ Furthermore, it is difficult to ascertain the irreproducible literature data.^[^ ^10^ ^]^ Each of these aspects can contribute to an unsatisfactory ML model performance. The automated experimental platforms, on the other hand, can generate reproducible and highly consistent data, which could improve the model performance, but the dataset size is usually limited. We recently demonstrated the advantages of automated fast‐flow antisense PMO and PNA synthesis over traditional batch techniques in terms of higher synthetic fidelity, improved purity, and significantly decreased synthesis time.^[^ ^11^ ^]^ The high‐throughput reproducible flow synthesis data can provide a foundation for building robust ML models to predict and improve synthesis quality.

ML algorithm advancement can aid in uncovering nonobvious complex relationships. In biological transformations, ML has been previously applied to identification of drug‐resistant cell phenotypes,^[^ ^12^ ^]^ analysis of singe‐cell metabolomics data,^[^ ^13^ ^]^ and prediction of antibody toxicity.^[^ ^14^ ^]^ Furthermore, the combination of state‐of‐the‐art ML with automated chemical synthesis platforms can facilitate drug lead design and therapeutic development. In this regard, ML methods have been recently used to assist organic synthesis design,^[^ ^15^ ^]^ and predict efficient organic synthetic pathways.^[^ ^16^ ^]^ In addition, ML has also found applications in facilitating biopolymer production, for example toward optimizing fast‐flow peptide synthesis,^[^ ^9b^ ^]^ discovering effective antimicrobial peptides through evolutionary algorithms,^[^ ^17^ ^]^ and designing nuclear‐targeting abiotic miniproteins.^[^ ^18^ ^]^ Overall, using ML algorithms to mine the complex dataset can unveil hidden patterns through performing data clustering, model regression, and trend prediction.

**Figure 1:**
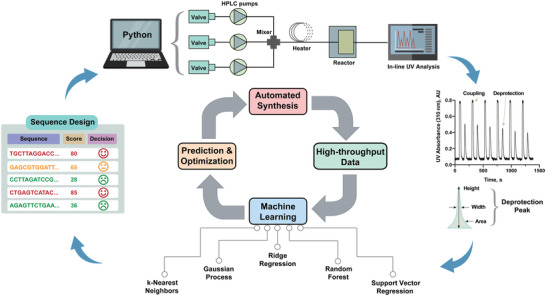
Combining machine learning (ML) with automated synthesis technology delivers a design‐build‐test‐learn cycle for PNA sequence design. A Python program‐controlled automated oligonucleotide synthesizer is used to synthesize PNAs, with a real‐time UV–Vis trace monitoring all coupling and deprotection reactions. ML was applied over the integral peak areas calculated from the deprotection steps in the experimental data. A trained and optimized ML model makes prediction on the synthesis efficiency for any arbitrary PNA sequences, and therefore, enables informed sequence design.

Here, we demonstrate that the in‐line collected synthesis UV data can be used to train effective ML models to predict the synthesis yield of PNA sequences (**Figure** **1**). After training and optimizing 10 different modern ML methods using 239 individual PNA coupling reactions, we developed a predictive ML model that allows for 93% prediction accuracy of the PNA synthesis. The predicted synthesis scores (defined as the deprotection peak area of the last coupling step after normalization to the average deprotection peak area of the first three lysine residues) were found to be highly correlated with the experimental high‐performance liquid chromatography (HPLC) crude purity, with a correlation coefficient *R* ^2^ = 0.95.

To further demonstrate the applicability of our optimized ML model toward efficient antisense PNA sequence design, we predicted all possible 18‐mer antisense PNA candidates targeting human dystrophin gene exon 44, which contributes to ≈8% of Duchenne muscular dystrophy (DMD) patients but currently lacks treatments.^[^ ^19^ ^]^ Three antisense PNA sequences were selected to represent easy, medium difficulty, and difficult sequences for synthesis, and the purified product yields validated the model predictions. To benefit DMD antisense therapy development, the top 100 synthetically facile antisense PNA sequences targeting the exon 44 were reported. Similarly, top antisense PNA sequences were designed as potential candidates to target therapeutically‐relevant genes that are associated with SARS‐CoV‐2, HIV‐1, as well as cardiovascular‐related diseases, type II diabetes, and solid tumors. Taken together, nominating candidates that are synthetically easy to obtain can accelerate the overall process of producing bioactive PNAs. As a step forward, in this study, we show that optimized ML models can guide efficient PNA sequence design and accelerate the process of antisense drug development.

## 2 Results and Discussion

### 2.1 Training Data was Compiled from a Fully Automated PNA Synthesizer

Recently, our laboratory developed a Python program‐controlled fully automated PNA synthesizer,^[^ ^11^ ^]^ which enables rapid formation of each amide bond in approximately 10 s, a process significantly more rapid than either commercial peptide synthesizers or routine batch protocols.^[^ ^11b^ ^]^ On our platform, the deprotection of fluorenylmethyloxycarbonyl (Fmoc) groups during PNA synthesis can be monitored using an in‐line UV–Vis detector (at 310 nm). Under optimized reaction conditions,^[^ ^11b^ ^]^ the Fmoc deprotection UV trace can be used as an indicator of the synthesis quality. To fully use this information, we attempted to quantitatively investigate the relationship between the deprotection UV traces and the overall PNA synthesis efficiency via ML. To our knowledge, such a standardized UV–Vis dataset on PNA synthesis was not previously accessible with conventional PNA synthesis protocols.

To prepare the training data for our ML algorithm, we installed a 3‐mer lysine linker on the C‐terminus of each PNA sequences for data normalization. The peak area of every deprotection peak was then computed in Pythonnvironment. The PNA sequence information was used to prepare training features and the deprotection peak areas were used as the response. Due to the peak variations caused by the resin amount loaded onto the synthesizer, the integral of deprotection peaks is normalized to the average peak area of the first three lysine residues. The final dataset obtained contains 239 unique PNA pre‐chain and nucleotide combinations.

### 2.2 ML Provides a Robust Tool for Accurate PNA Synthesis Prediction

**Figure 2:**
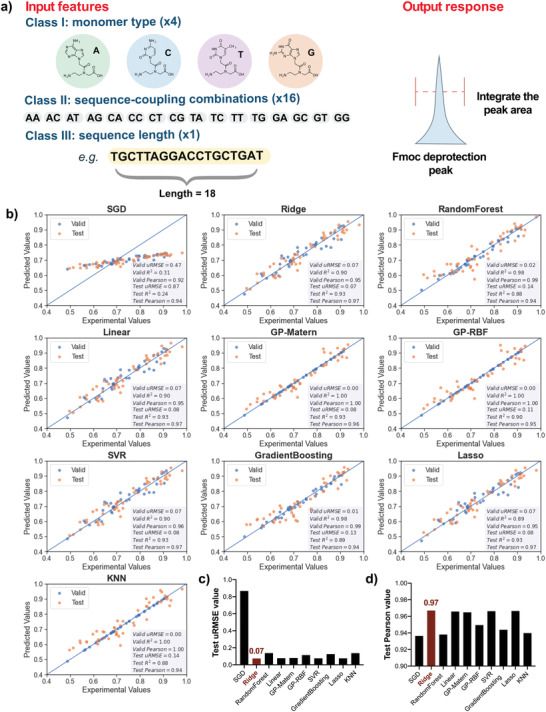
Benchmark 10 ML model architectures for accurate PNA synthesis prediction. a) The input features include 4 PNA monomers, 16 sequence‐coupling combinations, and sequence length. The integration of the Fmoc deprotection peak area is the output response. b) Performance of 10 different ML model architectures on validation and testing datasets, visualized using parity plots. Individual scatter plots have points in blue for sequences in the validation dataset, and points in orange for sequences in the held‐out testing dataset. Metrics for model performance, unitless/relative root‐mean‐squared‐error (uRMSE), *R* ^2^, and Pearson's correlation, have been noted for validation and testing datasets in the inset textboxes. Titles of the subplots refer to the specific model architectures. c) Test uRMSE values of 10 ML models of which Ridge model presents the lowest value: 0.07. d) Test Pearson values of 10 ML models of which Ridge model presents the highest score: 0.97. For more model performance details, see Tables S1 and S2 (Supporting Information). Abbreviations: SGD, stochastic gradient descent; GP, Gaussian process; SVR, support vector regression; RF, random forest; GB, gradient boosting; kNN, k‐nearest neighbors.

Establishing a reliable training approach is key to achieving accurate model prediction. Many modern ML methods can implement complex biological and chemical data analysis.^[^ ^18^, ^20^ ^]^ To find the best ML approach, we benchmarked the performance of 10 ML model architectures, i.e, Linear, Ridge, Lasso, stochastic gradient descent (SGD, SGD represents the “SGDRegressor” function that we used to train the model from scikit‐learn), gaussian process (GP) with two different kernel functions (“Matern” and “RBF”), support vector regression (SVR), random forest (RF), gradient boosting (GB), and k‐nearest neighbors (kNN). Three‐fold cross‐validation was used for a random split of 60% training, 20% validation, and 20% held‐out testing datasets.^[^ ^18^ ^]^ The input features consist of 21 different parameters: the PNA sequence length, 4 PNA monomers, and 16 possible sequence‐coupling combinations within the sequence, while the integrated Fmoc deprotection peak area is treated as the output response (**Figure** **2**a). The last‐step synthesis efficiency was used as the final prediction score for each input PNA sequence.

The compiled dataset collected on our automated PNA synthesizer was used to train and build all aforementioned ML architectures. After parameter optimization using a grid search approach, each optimized ML model was validated using the same validation dataset and their prediction accuracy was tested and compared using the same testing dataset. The model performances of 10 ML models were listed in Figure 2b. Except SGD, all models were able to achieve an optimal validation *R* ^2^ and Pearson's correlation coefficient, indicating robust model fitting. On the held‐out testing dataset, Ridge, Linear, Lasso, and SVR yielded the same Pearson's *r* correlation coefficient, but Ridge regression outperformed all other model architectures by achieving a unitless/relative root‐mean‐squared error (uRMSE) of 0.07. Thus, we selected the optimized ML model based on Ridge regression for subsequent experimental validations and predictions.

### 2.3 ML Informs the Feature Importance for Model Performance

Data mining over the training dataset informs the feature importance for model performance. The relative feature importance contributing to the model prediction was summarized using *n‐grams* representation approach and Ridge ML algorithm respectively (Figures S4 and S5, Supporting Information). In line with the common intuition, the PNA chain length was ranked as a top important feature in both cases. In addition, besides the sequence length, we observed that four PNA monomers, i.e., guanine (G), thymine (T), cytosine (C), and adenine (A), contribute significantly to the model performance. Overall, chain length and four monomers play a more important role than any of the 16 possible dimer permutations with respect to our model performance.

### 2.4 ML Predictions Agree with Experimental Data

**Figure 3:**
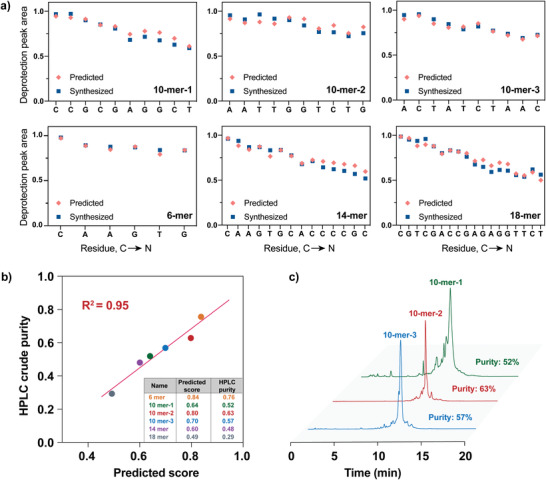
Predicted peptide nucleic acid (PNA) synthesis scores agree with experimental validation. a) Six PNA sequences were randomly generated, including three 10‐mers, one 6‐mer, one 14‐mer, and one 18‐mer. ML predicts the synthesis efficiency, denoted as deprotection peak area of each step, and the trace were found consistent with the experimentally recorded UV data. b) The HPLC crude purities of the six randomly generated PNAs show strong correlation (*R* ^2^ = 0.95) with ML‐predicted synthesis scores. c) The crude HPLC traces of three same‐length PNAs were compared to demonstrate the distinguishing capability of the ML model. Integration was applied over the main product peaks, as indicated by LC–MS data (Section S3, Supporting Information).

To experimentally validate the prediction accuracy of thoptimized ML model, we randomly generated six PNA sequences for re‐synthesis, including three 10‐mers, one 6‐mer, one 14‐mer, and one 18‐mer. Synthesis efficiency, denoted as the deprotection peak area at each coupling step, was predicted using the optimized ML model (**Figure** **3**a). The six randomly generated sequences were individually synthesized on the automated PNA synthesizer, and the in‐line deprotection data were collected and integrated. Notably, the experimental synthesis data were found highly consistent with the predicted traces (Figure 3a), indicating that our model enables an accurate prediction on the PNA synthesis quality based off sequences.

Side‐reactions such as monomer deletion, rearrangement, and isomerization can occur during PNA synthesis,^[^ ^11b^ ^]^ which cause lower reaction yield than predicted scores or potentially inconsistent results, and this information is difficult to track using UV–Vis surveillance. To validate the correlation between the ML‐predicted synthesis scores and the actual yield of the synthetic materials, all six synthesized PNAs were cleaved off the resin and their crude sample purities were measured. After HPLC analysis, the crude product yield was calculated via integration over the main product peaks, which were characterized with liquid chromatography–mass spectrometry (LC–MS, in Section S3, Supporting Information). As shown in Figure 3b,c, the HPLC crude purities of the six randomly generated PNAs show strong correlation (*R* ^2^ = 0.95) with ML‐predicted synthesis scores, suggesting that ML‐predicted synthesis scores can further indicate the crude product yield.

### 2.5 ML Designs Antisense PNA Sequences Targeting Various Diseases and Cancers

**Figure 4:**
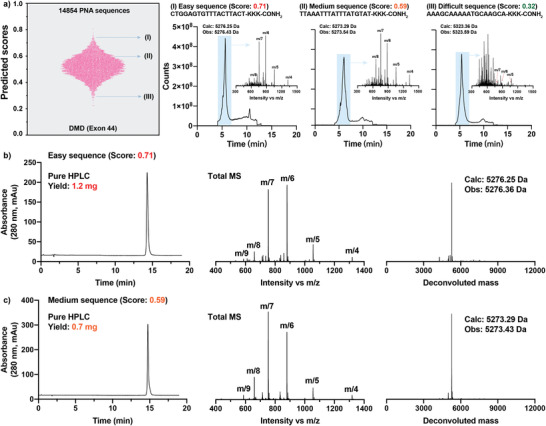
ML predicts “high value” antisense PNA sequences for DMD. a) Left, predicted scores for 14 854 18‐mer PNA sequences targeting exon 44 of human dystrophin gene; right, the crude total ion current (TIC) chromatogram and full range mass spectrum of three representative PNA sequences after individual synthesis. b) Yield, HPLC trace, total mass spectrum, and deconvoluted mass of purified easy sequence I. c) Yield, HPLC trace, total mass spectrum, and deconvoluted mass of purified medium sequence II. Failed to obtain pure product of difficult PNA sequence III after purification.

To further demonstrate the practical application of our optimized ML model, we predicted all the potential antisense PNA sequences (14854 18‐mers in total, **Figure** **4**a) targeting the exon 44 of human dystrophin gene, which contributes to ∼8% of all DMD patients and for which, at present, no treatment is available.^[^ ^19^ ^]^ To validate the prediction accuracy experimentally, we selected one easy sequence with scores >0.65 (sequence Ι, predicted score: 0.71), one sequence of medium synthetic difficulty with scores from 0.45 to 0.65 (sequence ΙΙ, predicted score: 0.59), and one difficult sequence with scores <0.45 (sequence ΙΙΙ, predicted score: 0.32), and synthesized them on the automated flow instrument. As the mass spectrum shows in Figure 4a, only trace amounts of the desired product were found for sequence ΙΙΙ, indicating an unsatisfactory synthesis. In contrast, for both sequences Ι and ΙΙ, the major peaks were identified as the desired products with an observation that sequence Ι presented a cleaner mass spectrum ion trace than sequence ΙΙ. Moreover, all the three PNA samples were purified with mass‐directed reversed‐phase HPLC (RP‐HPLC). After purification, 1.2 and 0.7 mg of pure products were obtained for easy PNA (sequence I) and medium PNA (sequence II), respectively (Figure 4b,c). We failed to obtain measurable amounts of pure product for the difficult PNA (sequence III) due to the observed low crude quality. Taken together, we confirmed that the PNA synthesis and purification outcomes are correlated with ML predictions. To potentially accelerate DMD antisense therapy development, we reported the top 100 easy antisense PNA sequences for targeting exon 44 of human dystrophin gene (sequences and predicted scores are available in Section S9, Supporting Information).

**Figure 5:**
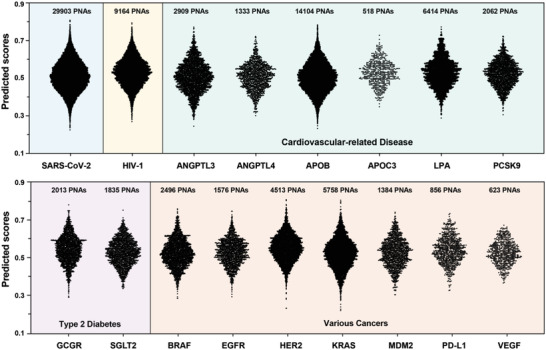
ML predicts synthetically accessible antisense PNA sequences for various diseases and cancer targets. Predicted scores for all possible 18‐mer PNA sequences targeting the whole genome of SARS‐CoV‐2 and HIV‐1, or mRNA sequences of ANGPTL3, ANGPTL4, APOB, APOC3, LPA, PCSK9, GCGR, SGLT2, BRAF, EGFR, HER2, KRAS, MDM2, PD‐L1, and VEGF. Top 100 antisense PNA sequences for each target can be found in Section S9 (Supporting Information).

In addition, to show a broad applicability of our ML model, we attempted to design antisense PNA sequences for viral diseases, cardiovascular‐related diseases, and various cancer types. Based on literature precedence, we selected two viral diseases, the ongoing COVID‐19 disease caused by the SARS‐CoV‐2 virus^[^ ^8^, ^11^ ^]^ and incurable HIV‐1,^[^ ^21^ ^]^ as well as six protein targets (ANGPTL3, ANGPTL4, APOB, APOC3, LPA, and PCSK9) for cardiovascular‐related diseases,^[^ ^22^ ^]^ two protein targets (GCGR and SGLT2) for type 2 diabetes,^[^ ^23^ ^]^ and seven protein targets (BRAF, EGFR, HER2, KRAS, MDM2, PD‐L1, and VEGF) for various cancers^[^ ^24^ ^]^ in consideration of their pharmaceutical potentials of developing antisense therapies (**Figure** **5**). After predicting all possible antisense PNAs targeting the corresponding mRNA coding regions of the aforementioned n targets, the top 100 most synthetically facile sequences were also reported (sequences and predicted scores can be found in Section S9, Supporting Information). In principle, the ML model can be used to guide antisense PNA sequence design for targeting any pharmaceutically relevant oligonucleotide sequences.

Collectively, we believe our ML prediction results are encouraging because the ability to design high‐yielding PNA sequences from a vast candidate pool can save significant amounts of lab effort and reduce the overall costs of the synthesis process. The presented data processing and ML workflow can be used in principle for similar stepwise flow chemistry reaction setups with the capability of in‐line analysis. Toward accelerating the antisense drug development, we envision our strategy, combining automated synthesis technology with ML algorithms, can also be applied to guide other oligonucleotide sequence design, e.g., PMO,^[^ ^11a^ ^]^ locked nucleic acid (LNA),^[^ ^25^ ^]^ or DNA with already demonstrated potentials for therapeutic development.

## 3 Conclusion

In this study, a large training dataset was generated on an automated PNA synthesizer, providing suitable input for the development of a robust ML algorithm. We then applied the optimized ML model to predict the efficiency of sequence‐dependent solid‐phase synthesis events. This model allows for accurate prediction of PNA synthesis efficiency and can serve as a useful tool to inform PNA sequence design.

Ten state‐of‐the‐art ML algorithms were compared in our study. Ridge stands out as a robust approach among tested ML methods after hyper parameter tuning and optimization, allowing for 93% prediction accuracy of the synthesis using PNA sequences as the only input. Moreover, the predicted synthesis scores were validated to have a strong correlation with the experimental HPLC crude purities.

As a broad application of our ML model, we showed that it can design antisense PNA sequences for genetic and viral diseases, as well as cardiovascular disorders and cancer. Several representative protein targets and two viral genomes were selected as showcases in consideration of their pharmaceutical potentials to develop antisense therapies, and top antisense PNA sequences were reported. To conclude, the ML model we developed here is effective to design synthetically accessible PNA sequences, with the potential to accelerate antisense oligonucleotide drug development.

## 4 Experimental Section

### Automated Flow PNA Synthesis and UV−Vis Data Collection

All PNA sequences were synthesized on a fully automated flow synthesizer, which was built in the Pentelute laboratory and described previously.^[^ ^11^ ^]^ The automated setup records every deprotection reaction efficiency in real‐time through an in‐line UV–Vis detector. Optimized synthesis conditions, as detailed in the previous publication, were used to synthesize all the PNA sequences. The following stock solutions were used for PNA synthesis: Fmoc and benzhydryloxycarbonyl (Bhoc) protected PNA monomers: Fmoc‐A(Bhoc)‐aeg‐OH, Fmoc‐G(Bhoc)‐aeg‐OH, Fmoc‐C(Bhoc)‐aeg‐OH, and Fmoc‐T‐aeg‐OH as a 0.2 m stock solution in *N*,*N*‐dimethylformamide (DMF), activating agent *N,N,N’,N’*‐tetramethyl‐*O*‐(1*H*‐benzotriazol‐1‐yl)uronium hexafluorophosphate (HBTU) as a 0.19 m stock solution in DMF, *N*,*N*‐diisopropylethylamine (DIEA) (10% v/v), and deprotection stock solution (20% piperidine, 2% formic acid, 78% DMF). DMF was pretreated with AldraAmine trapping agents >24 h before synthesis. Ten milligrams of H‐Rink amide resin (0.49 mmol g^−1^ loading) were used in all experiments in the dataset; details on resin and scale are given for synthesis examples in the Supporting Information. A standard synthesis cycle involves (a) prewashing of the resin, (b) iterative coupling, washing, deprotection, and washing steps per PNA monomer building block. Deprotection was performed with one‐part 20% piperidine, 2% formic acid (v/v) in DMF, and one‐part DMF for 50 s in the room‐temperature loop. UV–Vis in‐line analysis is recorded after passing the reactor and before waste collection. The UV synthesis data at a wavelength of 310 nm were collected for each individual deprotection step. The crude samples were cleaved off the resin and characterized with HPLC and LC–MS (for more details, see Supporting Information).

### Data Preprocessing

The raw UV–Vis dataset obtained from the automated system was exported as JSON files through its Python program. Exported JSON files were further processed using the customized Python codes. After baseline subtraction using the “baseline” function from module “peakutils,”^[^ ^26^ ^]^ peaks were identified using “find_peaks” function from the Python library SciPy, with the prominence level set at 20% of the maximum value, width defined smaller than 1000, and the rest of parameters left using default values. The “Integrate” function from SciPy library was used to calculate peak areas following the composite Simpson's rule. Only the deprotection peak areas were retained for subsequent model training.

### Featurization

To enable synthesis efficiency predictions directly from the PNA sequences, 21 training features were selected that consist of the PNA sequence length, quantities of each PNA monomer (i.e., A, T, C, and G), and 16 dimer combinations to enumerate all coupling conditions. As a response, the deprotection peak areas were normalized to the average of the first three lysine deprotections. The final processed dataset contains only unique PNA pre‐chain and nucleotide combinations (*n* = 239).

### Model Training and Hyperparameter Optimization

Ten ML model architectures, i.e, Linear, Ridge, Lasso, SGD, GP with two different kernel functions (“Matern” and “RBF”), SVR, RF, GB, and kNN, were implemented in the Python programming environment, version 3.9.5. Three‐fold cross‐validation was used for a random split of 60% training, 20% validation, and 20% held‐out testing datasets. Training datasets were used to train all the ML models before being validated using the validation dataset. Testing dataset was used to evaluate the model prediction performance after training and optimization. All ML algorithms were imported from module “sklearn,” and the hyperparameters were tuned using “GridSearchCV” function (imported from “sklearn.model_selection”). Optimized parameters can be found in Section S6 (Supporting Information). Otherwise, default values were applied.

### Statistical Analysis

A total of 239 unique PNA pre‐chain and nucleotide combinations were used to build and test the ML models in this study with 3‐fold cross‐validation and a random split of 143 samples for training, 48 samples for validation, and another 48 samples held‐out for testing. ML algorithms were imported from module “sklearn” in Python, and the hyperparameters were tuned using “GridSearchCV” function (imported from “sklearn.model_selection”). Data was pre‐processed according to the workflow described above.

Predicted top 100 PNA sequences for various diseases are included in the Supporting Information.

The Python code for automated operation of the flow synthesis instrument is available at: https://github.com/L‐Chengxi/MechWolf_Pull. All code used for training and optimization of the model is available at: https://github.com/genweizhang/Tiny_Tide.

## Conflict of Interest

B.L.P. is a co‐founder and/or member of the scientific advisory board of several companies focusing on the development of protein and peptide therapeutics. All other authors declare no competing interests.

## Supporting information

Supporting Information

## Data Availability

The data that support the findings of this study are available in the main text and supporting information of this article.
