# Supplementary material for: Machine Learning Guides Peptide Nucleic Acid Flow Synthesis and Sequence Design
Source: Adv Sci (Weinh). 2022 Oct 21;9(34):2201988. doi: 10.1002/advs.202201988 (PMC9731686; doi:10.1002/advs.202201988)
Supplement: Supplementary file 1 — Supporting Information [file ADVS-9-2201988-s001.pdf]

ADVANCED SCIENCE
Open Access

Supporting Information

for *Adv. Sci.*, DOI 10.1002/advs.202201988

Machine Learning Guides Peptide Nucleic Acid Flow Synthesis and Sequence Design

*Chengxi Li, Genwei Zhang, Somesh Mohapatra, Alex J. Callahan, Andrei Loas, Rafael Gómez-Bombarelli and Bradley L. Pentelute**

Supporting Information for

Machine Learning Guides Peptide Nucleic Acid Flow Synthesis and Sequence Design

*Chengxi Li[†], Genwei Zhang[†], Somesh Mohapatra, Alex J. Callahan, Andrei Loas, Rafael Gómez-Bombarelli and Bradley L. Pentelute**

Affiliations

Prof. Chengxi Li, Dr. Genwei Zhang, Alex J. Callahan, Dr. Andrei Loas, Prof. Bradley L. Pentelute

Department of Chemistry, Massachusetts Institute of Technology, 77 Massachusetts Avenue, Cambridge, MA 02139, USA.

Dr. Somesh Mohapatra, Prof. Rafael Gómez-Bombarelli

Department of Materials Science and Engineering, Massachusetts Institute of Technology, 77 Massachusetts Avenue, Cambridge, MA 02139, USA

Prof. Chengxi Li

College of Chemical and Biological Engineering, Zhejiang University, No.866 Yuhangtang Road, Hangzhou, Zhejiang, China, 310030

ZJU-Hangzhou Global Scientific and Technological Innovation Center, No.733 Jianshe San Road, Xiaoshan District, Hangzhou, Zhejiang, China, 311200

Prof. Bradley L. Pentelute

The Koch Institute for Integrative Cancer Research, Massachusetts Institute of Technology, 500 Main Street, Cambridge, MA 02142, USA

Center for Environmental Health Sciences, Massachusetts Institute of Technology, 77 Massachusetts Avenue, Cambridge, MA 02139, USA

Broad Institute of MIT and Harvard, 415 Main Street, Cambridge, MA 02142, USA

[*] Email: blp@mit.edu

[†] These authors contributed equally to this work.

This PDF file includes:

Materials and Methods
Figures S1 to S5
Table S1 to S2
Synthetic UV-vis Traces
HPLC, LC-MS traces

Table of Contents

1. General Information *4*
1.1 General reagent information 4
1.2 Analytical high-performance liquid chromatography (HPLC) analysis 5
1.3 Liquid chromatography–mass spectrometry (LC-MS) analysis 5
2. Preparation of PNAs via an automated flow-based instrument *5*
2.1 Introduction of automated PNA synthesizer 'Tiny Tides' 5
2.2 Automated Flow PNA Synthesis and UV−Vis Data Collection 7
2.3 PNA cleavage 8
2.4 ML Prediction and Experimental Validation 9
2.5 Design PNA sequences for various diseases and cancer protein targets 9
3. Synthetic UV-vis, HPLC, and LC-MS data for validation experiments *9*
4. LC-MS and HPLC data for anti-exon 44 PNA samples *22*
5. Analysis of features importance *32*
5.1 Analysis of features using *n-grams* representation approach 32
5.2 Analysis of features using *Ridge* model 33
6. Hyperparameters and optimized hyperparameters *33*
7. ML model architecture performance on validation and test data sets *35*
8. References *36*
9. Synthetically facile antisense PNA sequences for diseases predicted by ML *36*
9.1 Top 100 antisense PNA sequences for exon 44 of human dystrophin gene 36
9.2 Top 100 antisense PNA sequences for SARS-CoV-2 39
9.3 Top 100 antisense PNA sequences for HIV-1 41
9.4 Top 100 antisense PNA sequences for ANGPTL3 44
9.5 Top 100 antisense PNA sequences for ANGPTL4 47
9.6 Top 100 antisense PNA sequences for APOB 49
9.7 Top 100 antisense PNA sequences for APOC3 52
9.8 Top 100 antisense PNA sequences for LPA 55

9.9 Top 100 antisense PNA sequences for PCSK9 57

9.10 Top 100 antisense PNA sequences for GCGR... 60

9.11 Top 100 antisense PNA sequences for SGLT2 63

9.12 Top 100 antisense PNA sequences for BRAF.. 65

9.13 Top 100 antisense PNA sequences for EGFR.. 68

9.14 Top 100 antisense PNA sequences for HER2 71

9.15 Top 100 antisense PNA sequences for KRAS.. 73

9.16 Top 100 antisense PNA sequences for MDM2 76

9.17 Top 100 antisense PNA sequences for PD-L1 79

9.18 Top 100 antisense PNA sequences for VEGF.. 81

1. General Information

1.1 General reagent information

Synthesis reagents: 0.49 mmol/g H-Rink Amide resin was purchased from PCAS Biomatrix (The structure was shown in Figure S1). Fluorenylmethyloxycarbonyl (Fmoc) and benzhydryloxycarbonyl (Bhoc) protected PNA monomers, i.e., moA, moC, moG, and moT, were purchased from PNA bio. The H-Rink Amide resin and four monomer structures were shown in Figure S1. Fmoc-protected amino acids Fmoc-Lys (Boc)-OH was purchased from the Novabiochem-line from Sigma Millipore. *N,N,N′,N′*-tetramethyl-O-(1H-benzotriazol-1-yl) uronium hexafluoro-phosphate (HBTU, ≥97.0%) was purchased from P3 Biosystems. Diisopropylethylamine (DIEA, 99.5%), piperidine (≥99.0%), and formic acid (FA, ≥95.0%) were purchased from Sigma-Aldrich. *N*,*N*-Dimethylformamide (DMF, Biosynthesis OmniSolv® grade) was purchased from EMD Millipore (DX1732-1). AldraAmine trapping agents (for 1000-4000 mL DMF, catalog number Z511706) was purchased from Sigma-Aldrich.

Cleavage reagents: HPLC grade trifluoroacetic acid (TFA, ≥99.0%), triisopropylsilane (TIPS, ≥98.0%) and GC grade 1,2-ethanedithiol (EDT, ≥98.0%) were purchased from Sigma-Aldrich.

Purification and analytical reagents: Water for HPLC was purified to 18.2 MΩ·cm resistivity on a Millipore Milli-Q system. HPLC-grade acetonitrile was purchased from VWR International (Philadelphia, PA). LC-MS grade acetonitrile was purchased from Sigma-Aldrich (St. Louis, MO). Unless specified otherwise, all other reagents and solvents were purchased from Sigma-Aldrich and kept over activated 3 Å molecular sieves, they were used directly without further purification.

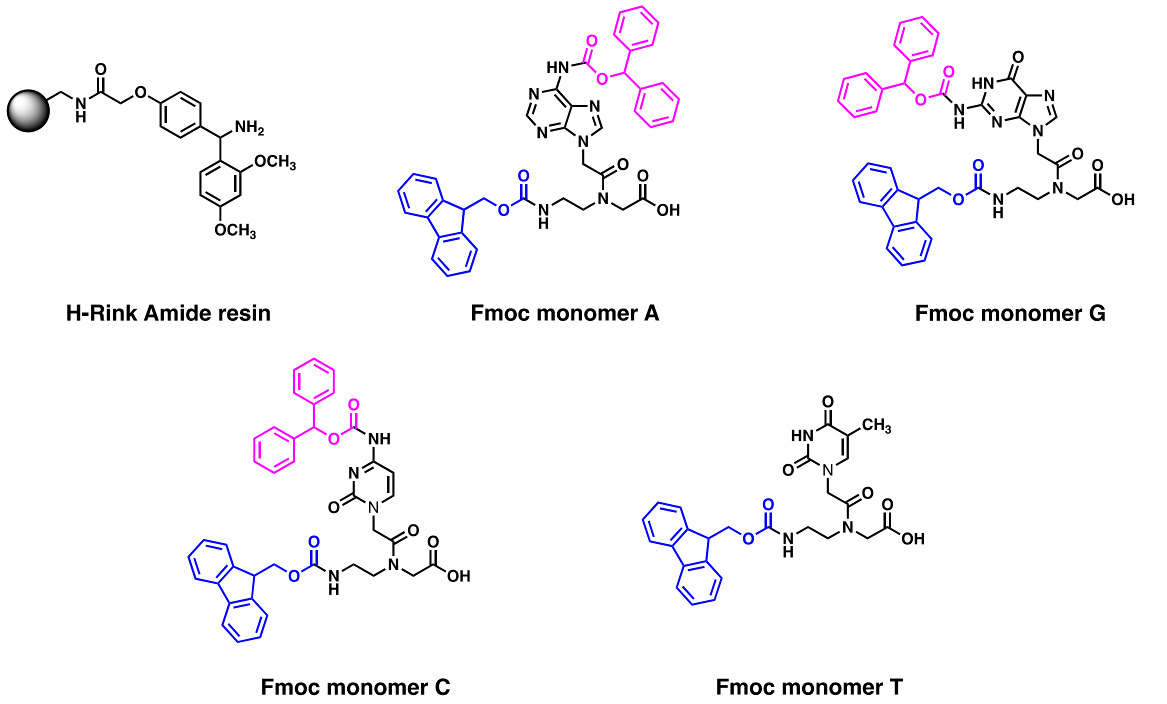

Figure S1: Structure of the H-Rink Amide resin and Fmoc-protected PNA monomers.

1.2 Analytical high-performance liquid chromatography (HPLC) analysis

All crude PNA samples were dissolved with 30% acetonitrile in water (with 0.1% TFA additive), the solutions were filtered and then diluted to approximately 0.1 mg/mL concentration. The sample purity analysis was carried out on an Agilent 1200 series HPLC system.

Conditions: Phenomenex Kinetex column (100 x 2.1 mm, 2.6 um, 100 Å silica) was used; Flow rate was set at 0.375 mL/minute; UV detection was performed at 280 nm. A linear gradient of acetonitrile with 0.08% TFA additive (solvent B) in water with 0.1% TFA additive (solvent A) was used. Method: 0-2 min: hold 2% B; 2-17 min: 2-32% B; 17-17.5 min: 32-65% B; 17.5-19 min: hold 65% B; 19-25 min: 2% B. The crude HPLC purities were determined by manual integration of all detectable peak signals, and the original traces were shown in ***Section S3***.

1.3 Liquid chromatography–mass spectrometry (LC-MS) analysis

The main peaks from the HPLC were loaded onto an Agilent 1290 Infinity HPLC, and the mass was analyzed by an Agilent 6550 Q-TOF with Dual Jet Stream ESI ionization and iFunnel.

Conditions: The mass spectrometer was run in positive ionization mode with extended dynamic range (2GHz), and low mass range (m/z in range 100 to 1700). Solvents: 0.1% formic acid in H_2O (solvent A) and 0.1% formic acid in acetonitrile (solvent B). Column: Phenomenex Luna C18 (3 um, 1 x 150 mm, 100 Å silica); Flow Rate: 0.4 mL/min; Gradient: 1% B 0-1 min, linearly ramp from 1% B to 61% B 1 to 8 min, then 61%-99% 8-9 min. Data were processed using Agilent MassHunter Workstation Qualitative Analysis Version B.06.00 with BioConfirm software.

2. Preparation of PNAs via an automated flow-based instrument

2.1 Introduction of automated PNA synthesizer 'Tiny Tides'

All the PNA sequences studied in this work were prepared on an automated PNA synthesizer, 'Tiny Tides', which was designed previously in our lab.[1,2] This automated instrument contains seven major modules: a central control computer, three HPLC pumps, reaction zone, a UV-visible detector, a solution storage system, heating elements, and three multi-position valves. All the modules were controlled by a modular script under the Mechwolf programming environment. An overview of the Tiny Tides was presented in ***Figure S2***, and every individual part was labeled with corresponding names. More designing details on this automated synthesizer can be found in our recently published work.[1,2]

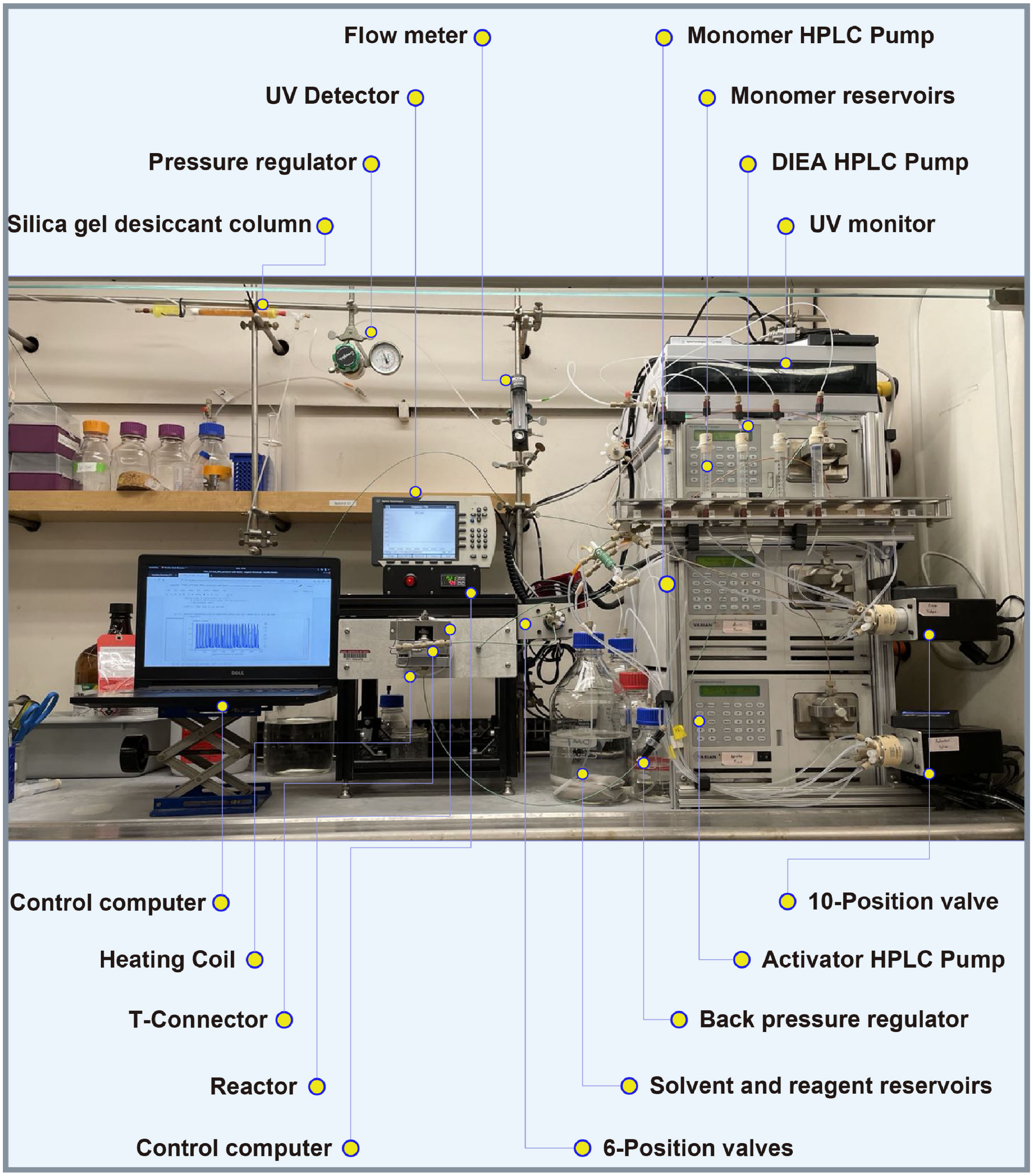

Figure S2. Overview of the automated synthesizer with the major components labeled in position.

2.2 Automated Flow PNA Synthesis and UV−Vis Data Collection

All PNA sequences were synthesized on a fully automated flow synthesizer, which was built in the Pentelute lab and described previously.[1,2] The automated setup records every deprotection reaction efficiency in real-time through an in-line UV-vis monitor. Optimized synthesis conditions, as detailed in our previous publication,[2] were used to synthesize all the PNA sequences. The following stock solutions were used for PNA synthesis: Fmoc and benzhydryloxycarbonyl (Bhoc) protected PNA monomers: Fmoc-A(Bhoc)-aeg-OH, Fmoc-G(Bhoc)-aeg-OH, Fmoc-C(Bhoc)-aeg-OH, Fmoc-T-aeg-OH as a 0.2 M stock solution in DMF, activating agent *N,N,N',N'*-tetramethyl-*O*-(1*H*-benzotriazol-1-yl)uronium hexafluorophosphate (HBTU) as a 0.19 M stock solution in DMF, DIEA (10% v/v), and deprotection stock solution (20% piperidine, 2% formic acid, 78% DMF v/v/v). DMF was pretreated with AldraAmine trapping agents >24 h before synthesis. Ten milligrams of H-Rink amide resin (0.49 mmol/g loading) were used in all experiments in the data set. A standard synthesis cycle involves (a) prewashing of the resin, (b) iterative coupling, washing, deprotection, and washing steps per PNA monomer building block. No capping or multiple couplings were needed, and each coupling cycle took 3 minutes. The workflow, timeline, and reagents for a complete coupling cycle were shown in ***Figure S3***. Steps 1-6 were repeated until the elongations of all residues completed. Deprotection was performed with one-part 20% piperidine, 2% formic acid (v/v) in DMF, and one-part DMF for 50 seconds in the room-temperature loop. UV-vis in-line analysis is recorded after passing the reactor and before waste collection. The UV synthesis data at a wavelength of 310 nm were collected for 239 individual deprotection steps from PNA synthesis experiments. The crude samples were cleaved off the resin and characterized with HPLC and LC-MS.

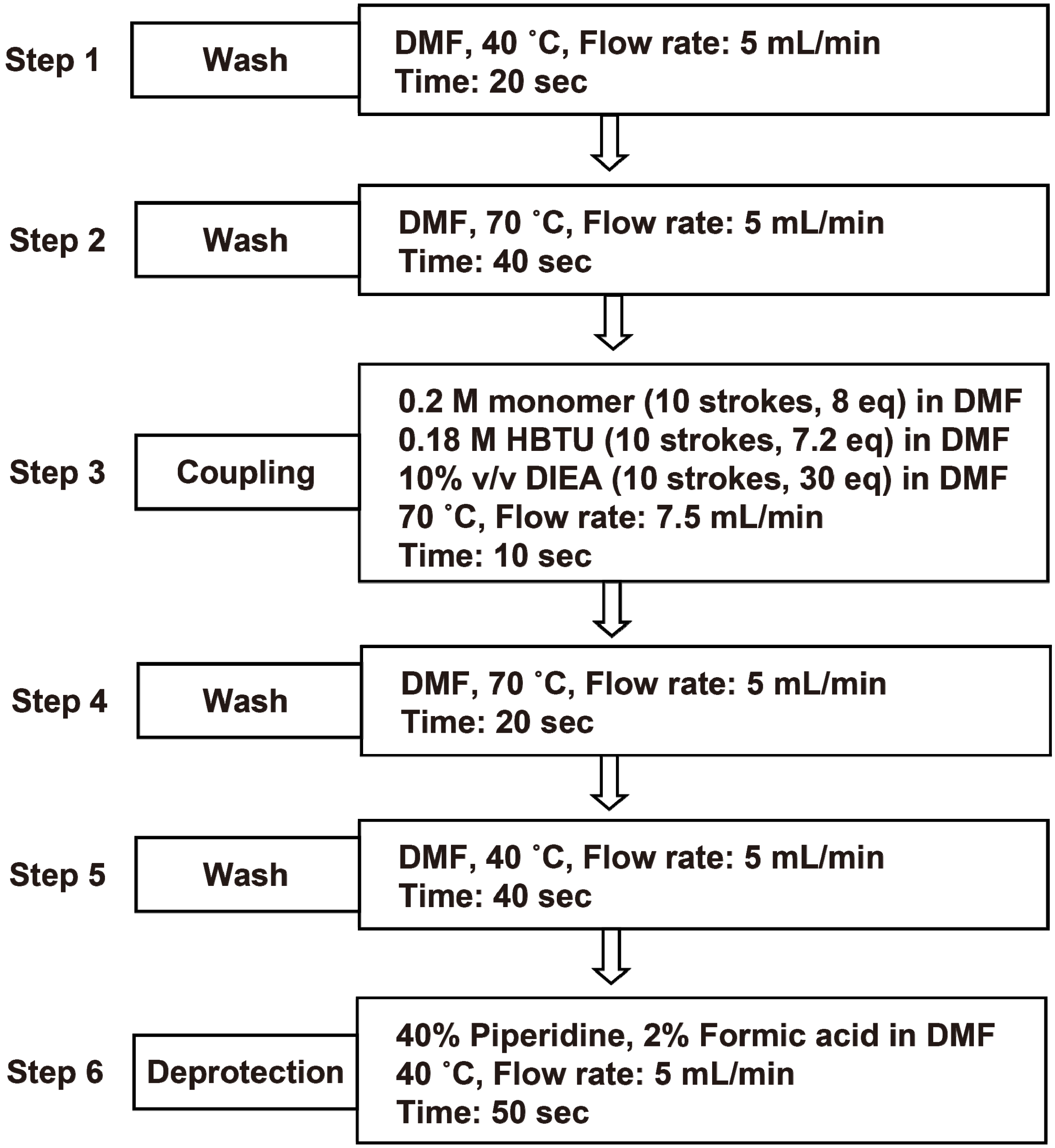

Figure S3. Workflow of a complete coupling cycle, performed during automated flow PNA synthesis.

2.3 PNA cleavage

Synthesized PNAs were cleaved off the solid support using trifluoroacetic acid (TFA) for purity characterizations. In brief, the cleavage from the resin and all side-chain group deprotection were carried out simultaneously with a cleavage cocktail containing 2.5% (v/v) 1,2-ethanedithiol (EDT), 2.5% (v/v) water, and 1% (v/v) triisopropylsilane in neat TFA for 2.0 h at room temperature. Five mL of the cleavage cocktail was used for approximately 0.1 mmol

compound. The cleaved crude PNAs were washed with dry ice-cold ether and followed by centrifugation at 4,000 rpm for 3 min for precipitation. The resultant solids were then dissolved in water/acetonitrile (50:50, v/v) and dried through lyophilization.

2.4 ML Prediction and Experimental Validation

Six PNA sequences with the length varied from 6- to 18-mer were randomly generated and the synthesis predictions were performed using the optimized ML model. To validate the model prediction results, all 6 PNA sequences were experimentally synthesized on our automated flow PNA synthesizer. UV-vis in-line records were compared with ML-predicted traces. Furthermore, the synthesized PNAs were cleaved off the resin and their crude purities were measured using HPLC. The PNA crude purities were compared with ML-predicted scores and the correlation strength was calculated.

2.5 Design PNA sequences for various diseases and cancer protein targets

All viral genome and mRNA sequences for the selected disease and cancer types were retrieved from GenBank. The accession numbers for human dystrophin gene exon 44, SARS-CoV-2, HIV-1, ANGPTL3, ANGPTL4, APOB, APOC3, LPA, PCSK9, GCGR, SGLT2, BRAF, EGFR, HER2, KRAS, MDM2, PD-L1, and VEGF are M81257.1, NC_045512.2, NC_001802.1, NM_014495.4, KR711164.1, NM_000384.3, NM_000040.3, NM_005577.4, EF692496.1, NM_000160.5, BC131542.1, M95712.2, U48722.1, M11730.1, M54968.1, GQ848196.1, AY254342.1, and AY047581.1, respectively. The reverse complement sequences were prepared using an online tool (https://www.bioinformatics.org) before inputting into the ML pipeline for synthesis quality predictions.

3. Synthetic UV-vis, HPLC, and LC-MS data for validation experiments

All PNAs including one 6-mer, three 10-mers, one 14-mer and one 18-mer, were synthesized automatically on synthesizer 'Tiny Tides' under the conditions depicted in ***Section S2.2*** workflow. Then PNA cleavage was performed following conditions in ***Section S2.3***. After cleavage, the crude purity of each PNA sample was analyzed with HPLC using the methods in ***Section S1.2*** and determined through integration of the HPLC chromatogram. The major peak of each sample was characterized with LC-MS under the conditions in ***Section S1.3***.

The synthetic UV-vis (310 nm), HPLC, and LC-MS data of all six validation PNAs were shown as below. The wide and tall peaks indicate the coupling steps, and the narrow but sharp peaks after each coupling peak represent fluorenylmethyloxycarbonyl (Fmoc) deprotection. Both of the coupling and deprotection peaks were labeled with a "×" symbol. The relative peak areas were integrated through a python script. Of note, there are some undesired peaks right after the

deprotection peak, which was caused by the ‘air bubble’ generated during valve switch, and these peaks were omitted in the machine learning training data set.

Sample: 6mer in **Figure 6**

Sequence: GTGAAC-KKK-$CONH_2$.

Synthesis method: Automated flow synthesis.

Resin: 10 mg Rink Amide resin (0.49 mmol/g).

Tiny Tides in-line synthetic data at UV absorbance 310 nm.

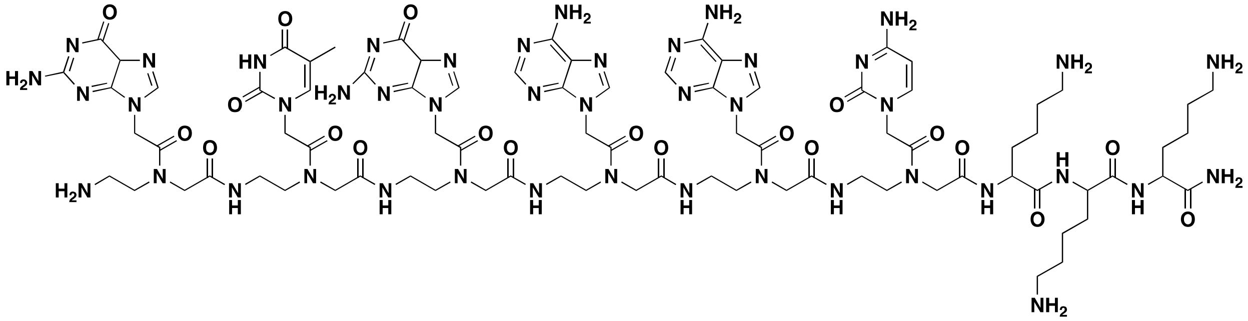

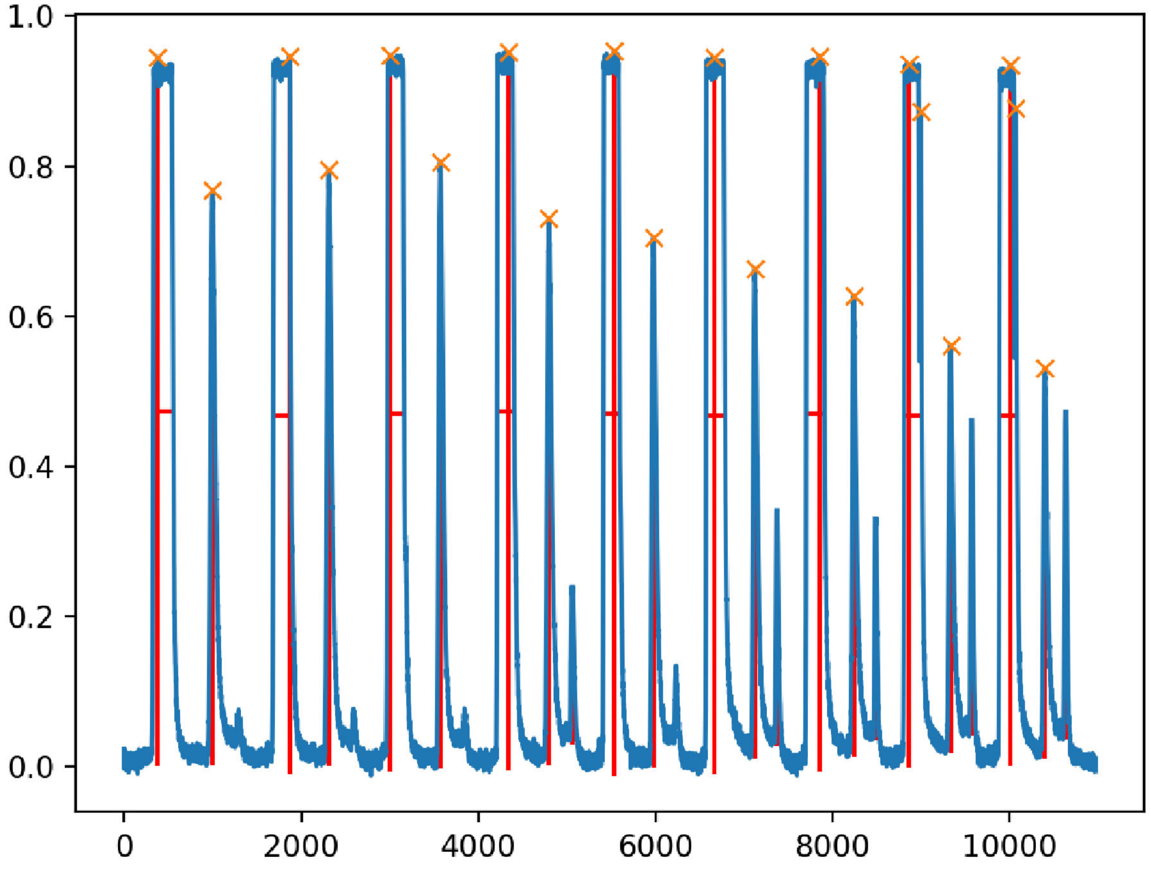

6mer	**1**	**2**	**3**	**4**	**5**	**6**
Predicted	0.978	0.900	0.848	0.870	0.810	0.835
Synthesized	0.970	0.887	0.843	0.877	0.769	0.839

HPLC method: see ***Section S1.2***
Crude purity: 76%

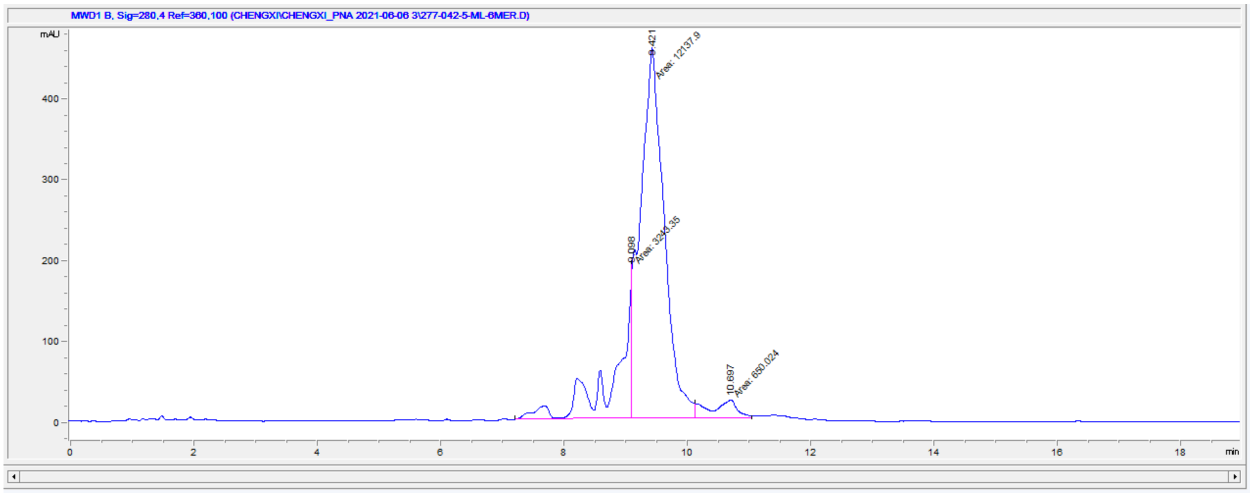

#	Time	Area	Height	Width	Area%	Symmetry
1	9.098	3243.4	188.4	0.287	20.231	7677.85
2	9.421	12137.9	458.6	0.4412	75.714	0.898
3	10.697	650	22.1	0.4891	4.055	2.694

LC-MS method: see ***Section S1.3***
Calculated: 2050.96 Da
Observed: 2050.98 Da

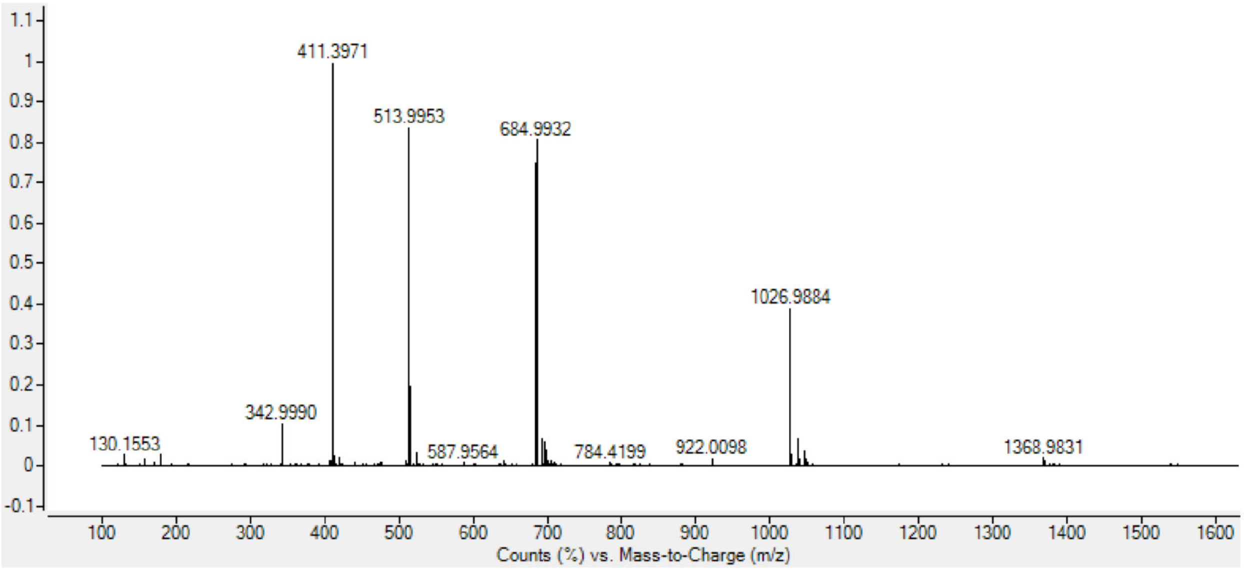

Sample: 10mer-1 in **Figure 6**
Sequence: TCGGAGCGCC-KKK-CONH2.
Synthesis method: Automated flow synthesis.
Resin: 10 mg Rink Amide resin (0.49 mmol/g).
Tiny Tides in-line synthetic data at UV absorbance 310 nm.

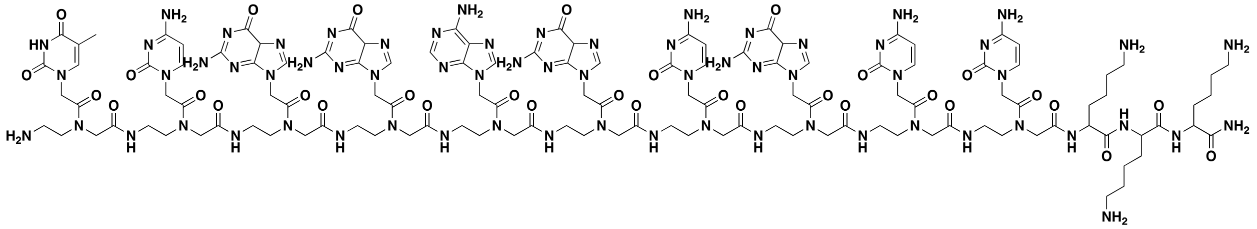

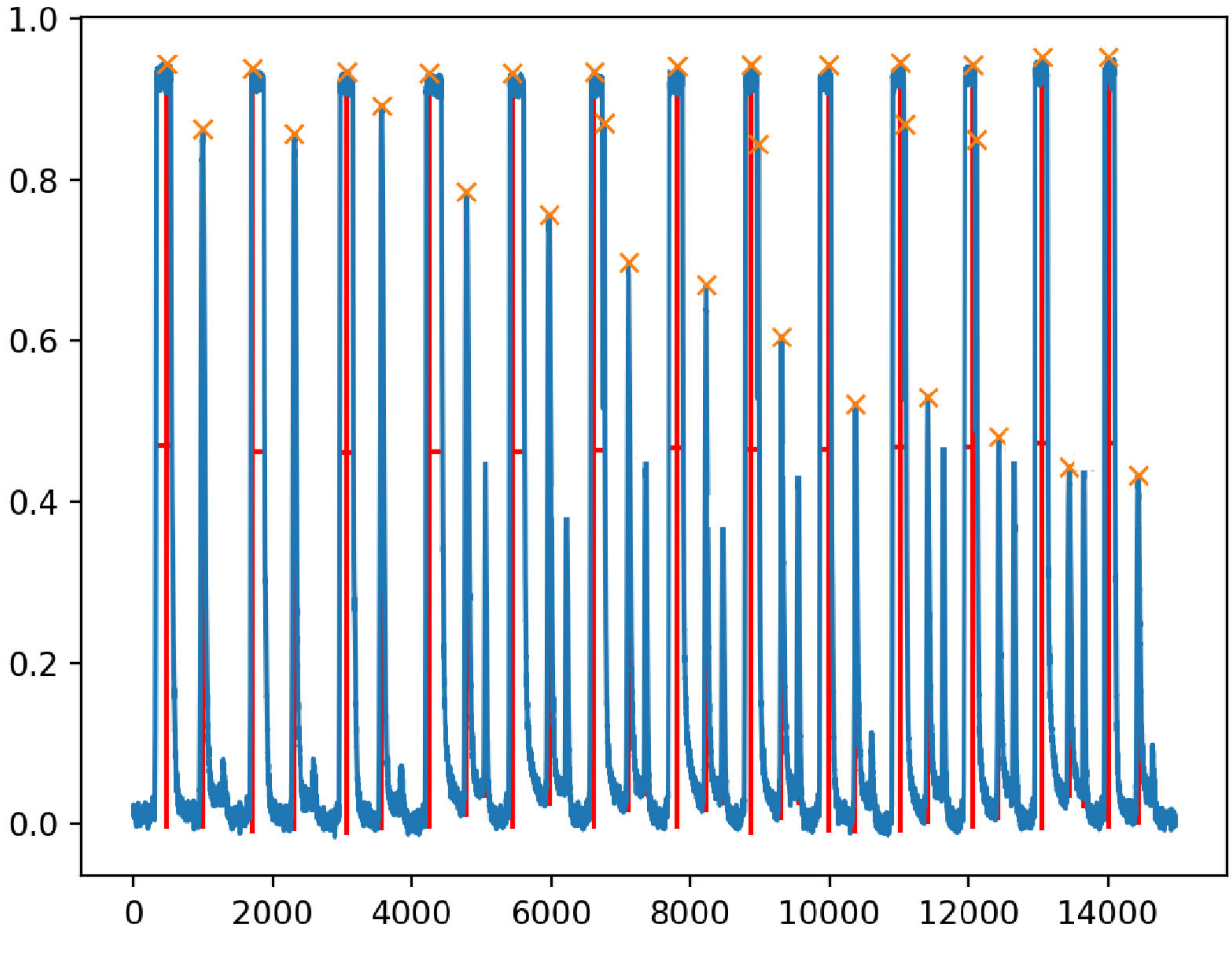

10mer-1	**1**	**2**	**3**	**4**	**5**	**6**	**7**	**8**	**9**	**10**
Predicted	0.994	0.983	0.900	0.872	0.811	0.710	0.730	0.718	0.702	0.660
Synthesized	0.970	0.955	0.938	0.873	0.856	0.770	0.804	0.789	0.724	0.636

HPLC method: see ***Section S1.2***
Crude purity: 52%

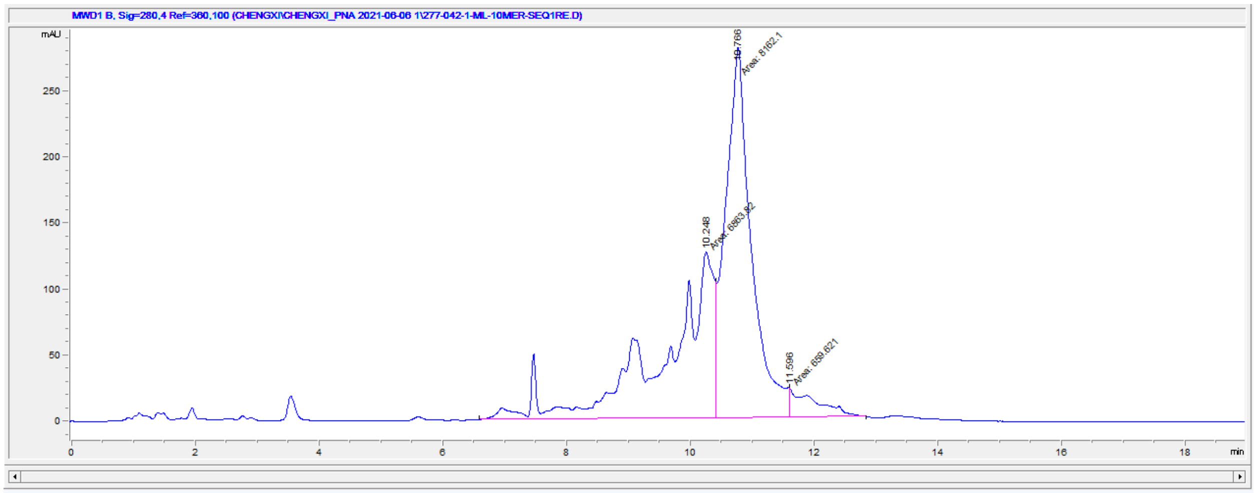

#	Time	Area	Height	Width	Area%	Symmetry
1	10.248	6863.8	125.7	0.9104	43.759	5.271
2	10.766	8162.1	280.4	0.4852	52.036	0.893
3	11.596	659.6	22.4	0.4913	4.205	0

LC-MS method: see ***Section S1.3***
Calculated: 3111.37 Da
Observed: 3111.39 Da

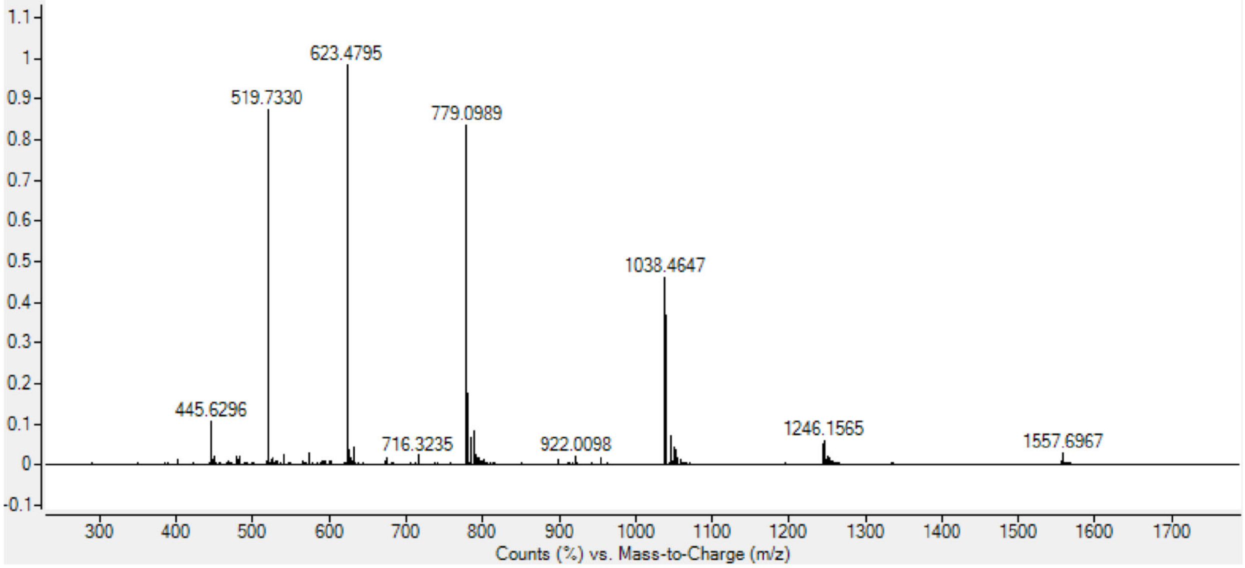

Sample: 10mer-2 in **Figure 6**
Sequence: AATTGGTCTG-KKK-CONH2.
Synthesis method: Automated flow synthesis.
Resin: 10 mg Rink Amide resin (0.49 mmol/g).
Tiny Tides in-line synthetic data at UV absorbance 310 nm.

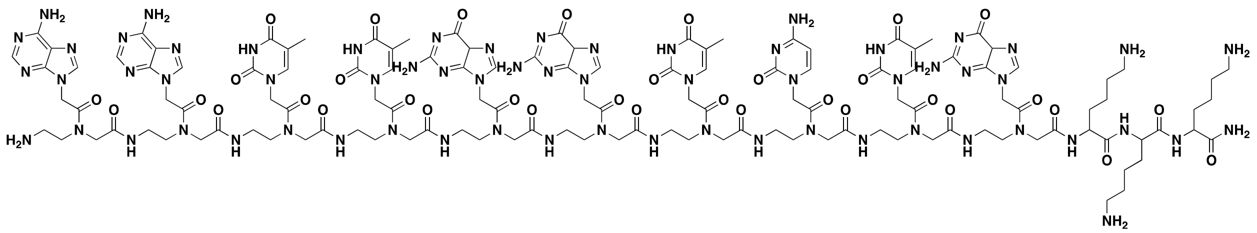

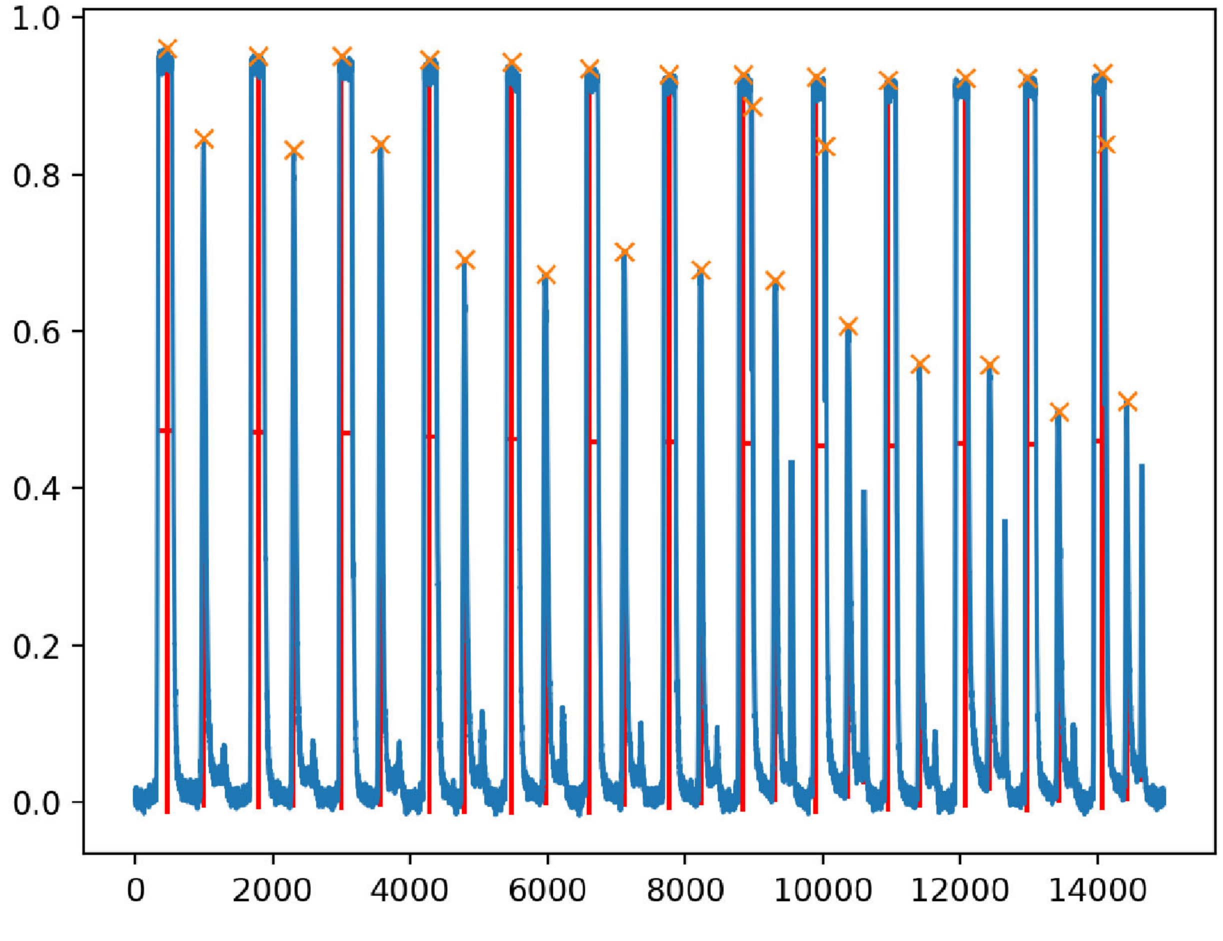

10mer-2	**1**	**2**	**3**	**4**	**5**	**6**	**7**	**8**	**9**	**10**
Predicted	0.931	0.869	0.866	0.856	0.884	0.853	0.798	0.815	0.755	0.785
Synthesized	0.891	0.847	0.856	0.836	0.906	0.891	0.783	0.819	0.731	0.801

HPLC method: see ***Section S1.2***
Crude purity: 63%

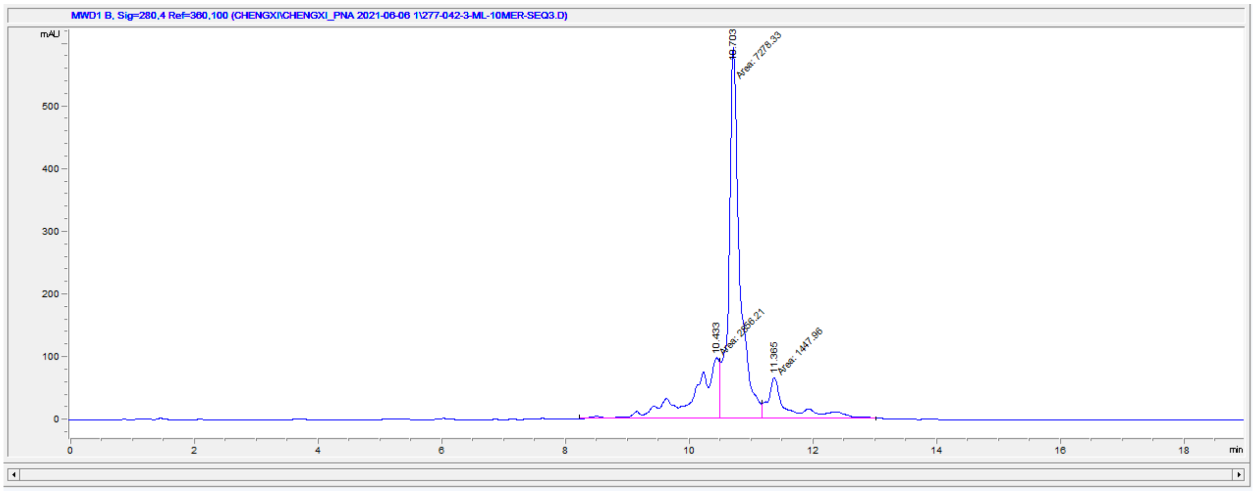

#	Time	Area	Height	Width	Area%	Symmetry
1	10.433	2856.2	96.8	0.4918	24.660	8.175
2	10.703	7278.3	592.5	0.2047	62.839	0
3	11.365	1448	65.1	0.3706	12.501	0.407

LC-MS method: see ***Section S1.3***
Calculated: 3140.37 Da
Observed: 3140.39 Da

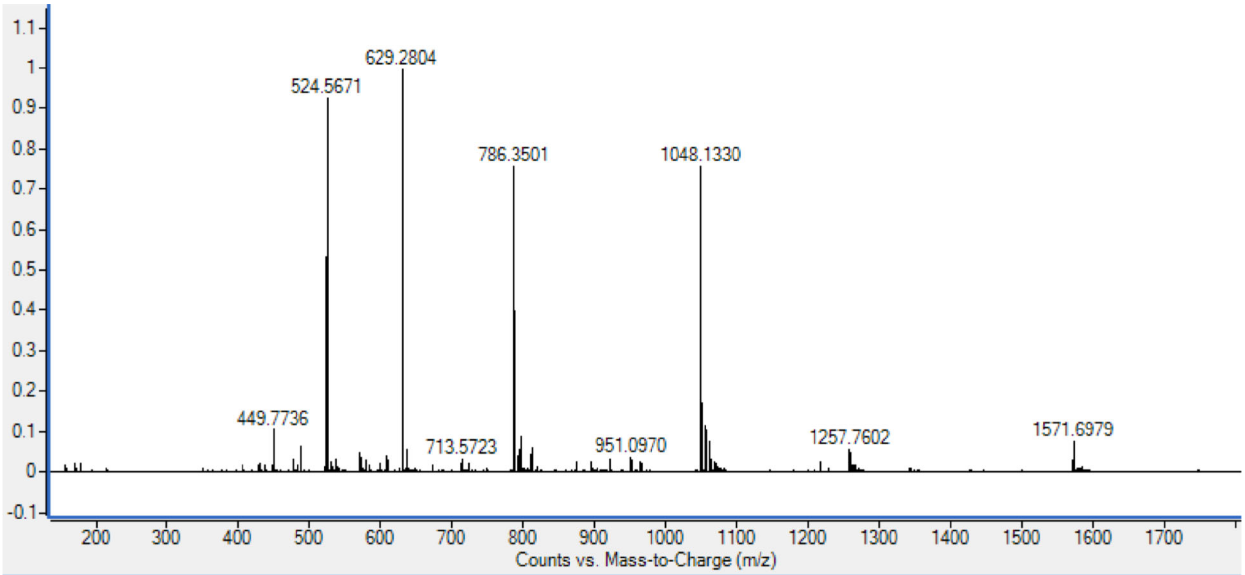

Sample: 10mer-3 in **Figure 6**
Sequence: ACTATCTAAC-KKK-CONH2.
Synthesis method: Automated flow synthesis.
Resin: 10 mg Rink Amide resin (0.49 mmol/g).
Tiny Tides in-line synthetic data at UV absorbance 310 nm.

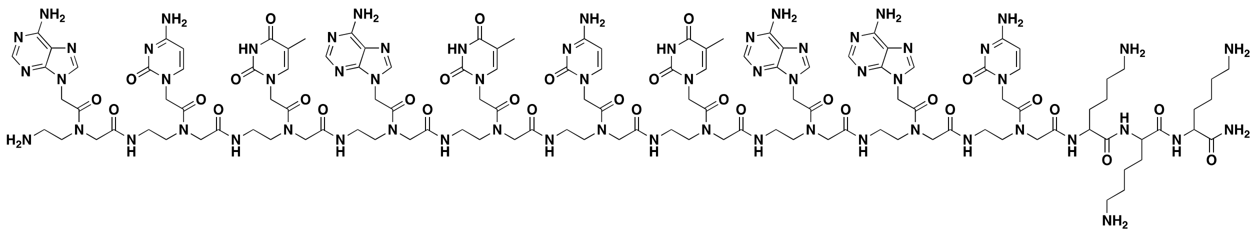

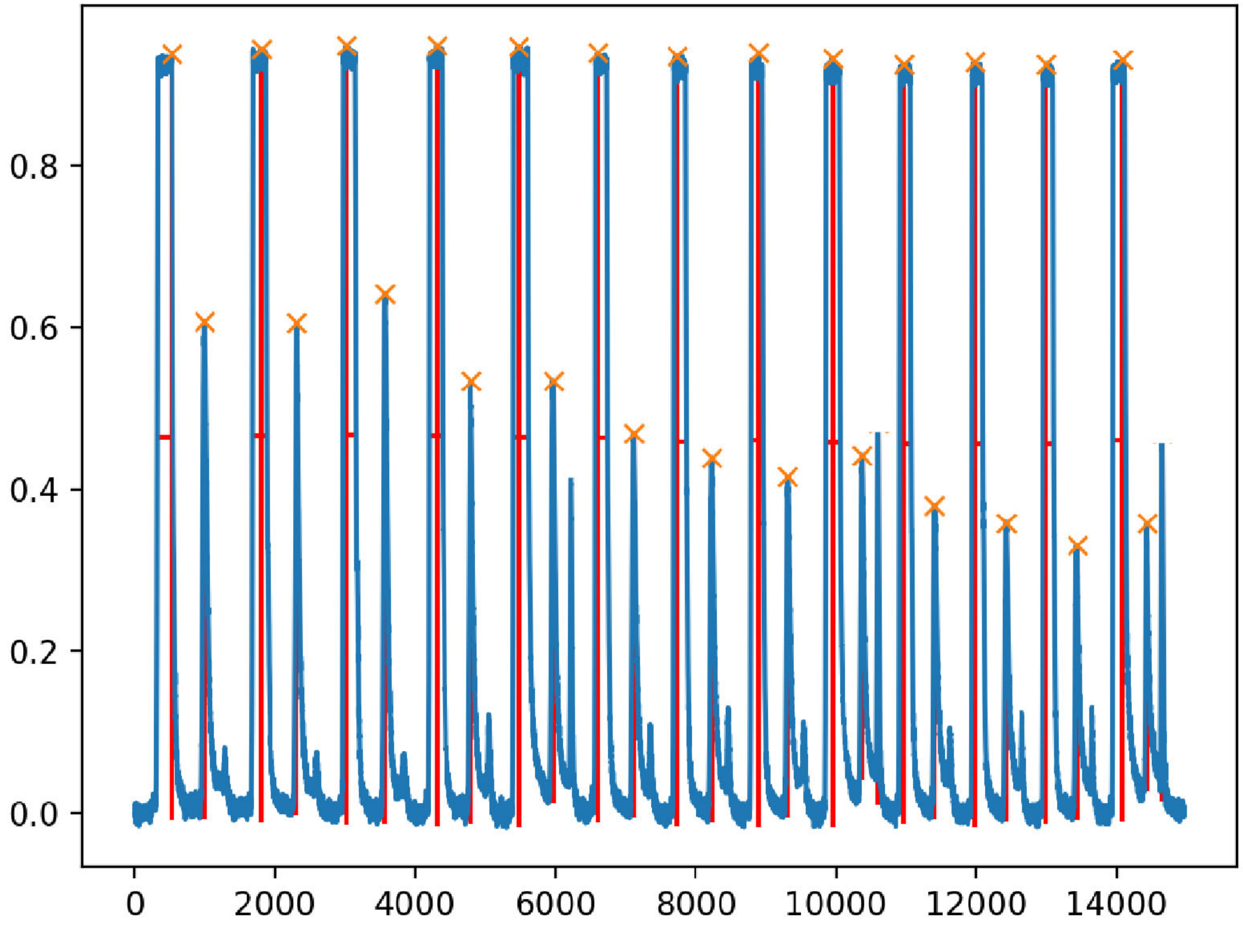

10mer-3	**1**	**2**	**3**	**4**	**5**	**6**	**7**	**8**	**9**	**10**
Predicted	0.936	0.948	0.891	0.849	0.861	0.849	0.784	0.745	0.687	0.729
Synthesized	0.891	0.929	0.841	0.797	0.806	0.842	0.754	0.711	0.667	0.705

HPLC method: see ***Section S1.2***
Crude purity: 57%

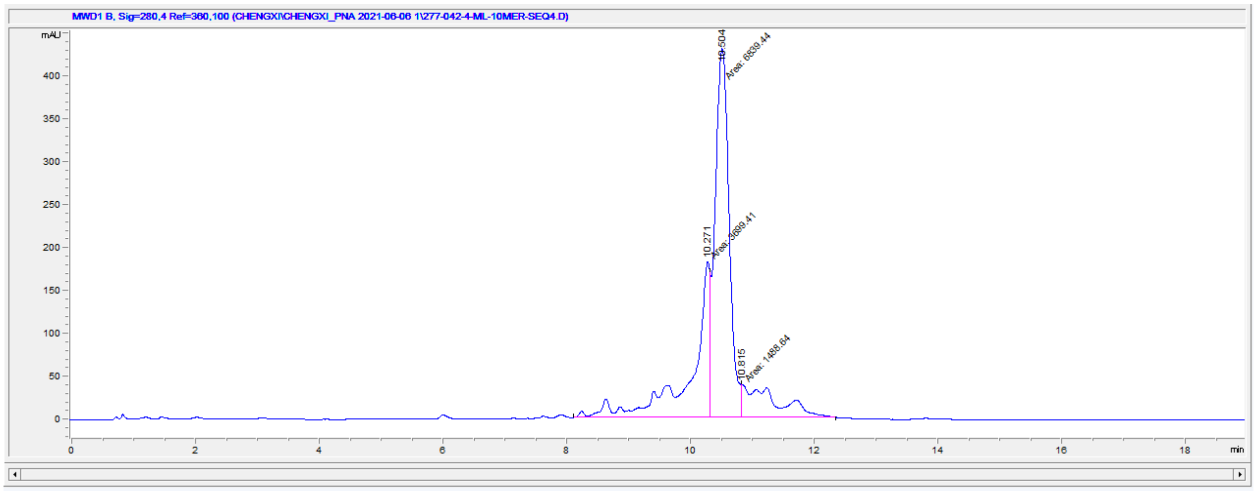

#	Time	Area	Height	Width	Area%	Symmetry
1	10.271	3699.4	181.6	0.3394	30.758	7.883
2	10.504	6839.4	430.3	0.2649	56.865	0.97
3	10.815	1488.6	38.4	0.6458	12.377	0

LC-MS method: see ***Section S1.3***
Calculated: 3053.37 Da
Observed: 3053.41 Da

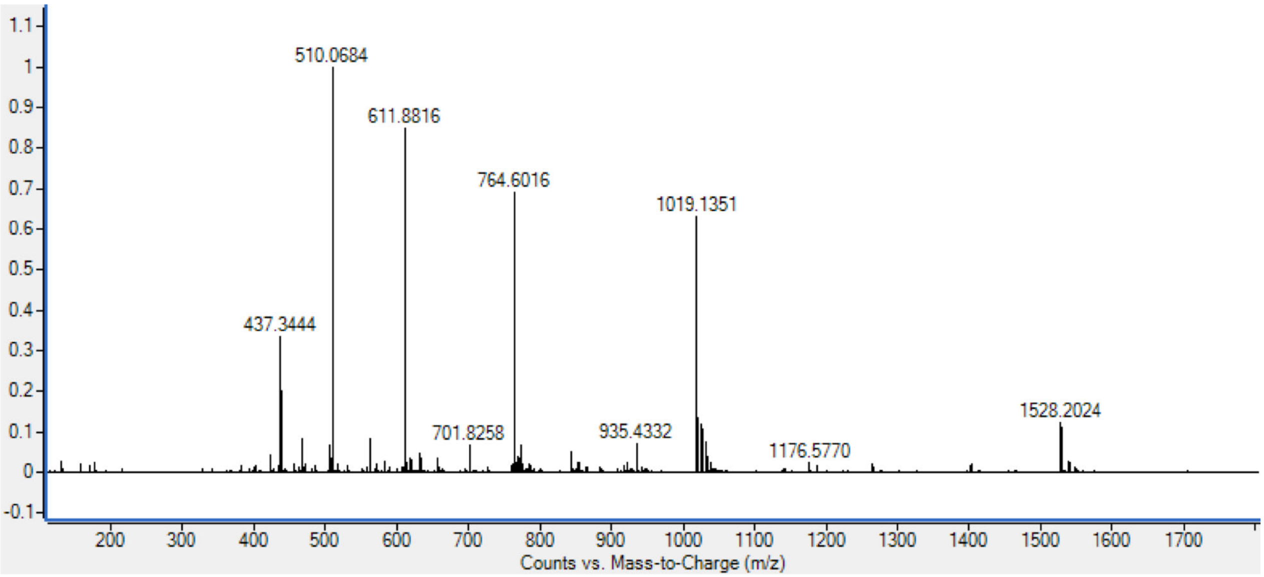

Sample: 14mer in **Figure 6**
Sequence: CAAGTGCACCCCGC-KKK-$CONH_2$.
Synthesis method: Automated flow synthesis.
Resin: 10 mg Rink Amide resin (0.49 mmol/g).
Tiny Tides in-line synthetic data at UV absorbance 310 nm.

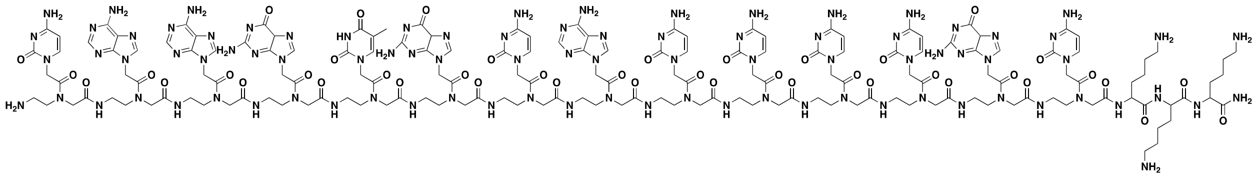

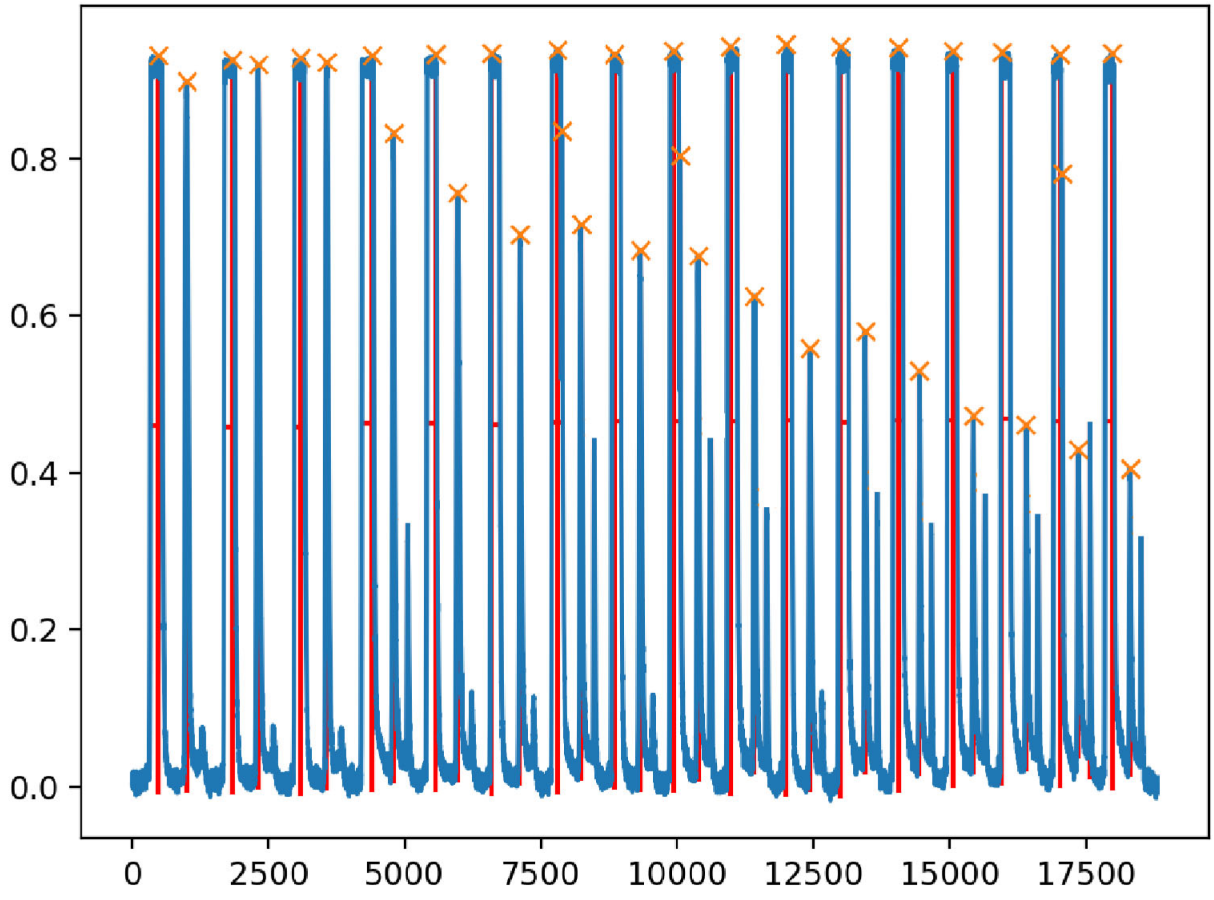

14mer	**1**	**2**	**3**	**4**	**5**	**6**	**7**	**8**	**9**	**10**
Predicted	0.965	0.892	0.831	0.850	0.789	0.824	0.793	0.722	0.745	0.706
Synthesized	0.970	0.887	0.843	0.877	0.769	0.839	0.774	0.691	0.729	0.713
	**11**	**12**	**13**	**14**						
Predicted	0.688	0.671	0.625	0.598						
Synthesized	0.697	0.682	0.665	0.600						

HPLC method: see ***Section S1.2***
Crude purity: 48%

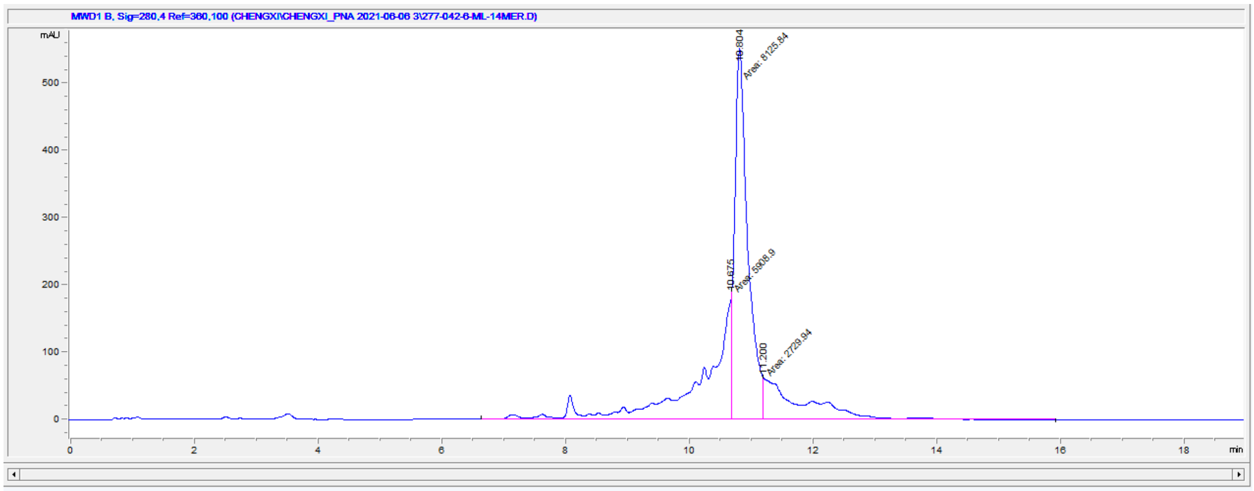

#	Time	Area	Height	Width	Area%	Symmetry
1	10.675	5908.9	185.6	0.5307	35.246	1.832
2	10.804	8125.8	552.5	0.2451	48.470	0.517
3	11.2	2729.9	61.3	0.7417	16.284	0

LC-MS method: see ***Section S1.3***
Calculated: 4123.79 Da
Observed: 4123.82 Da

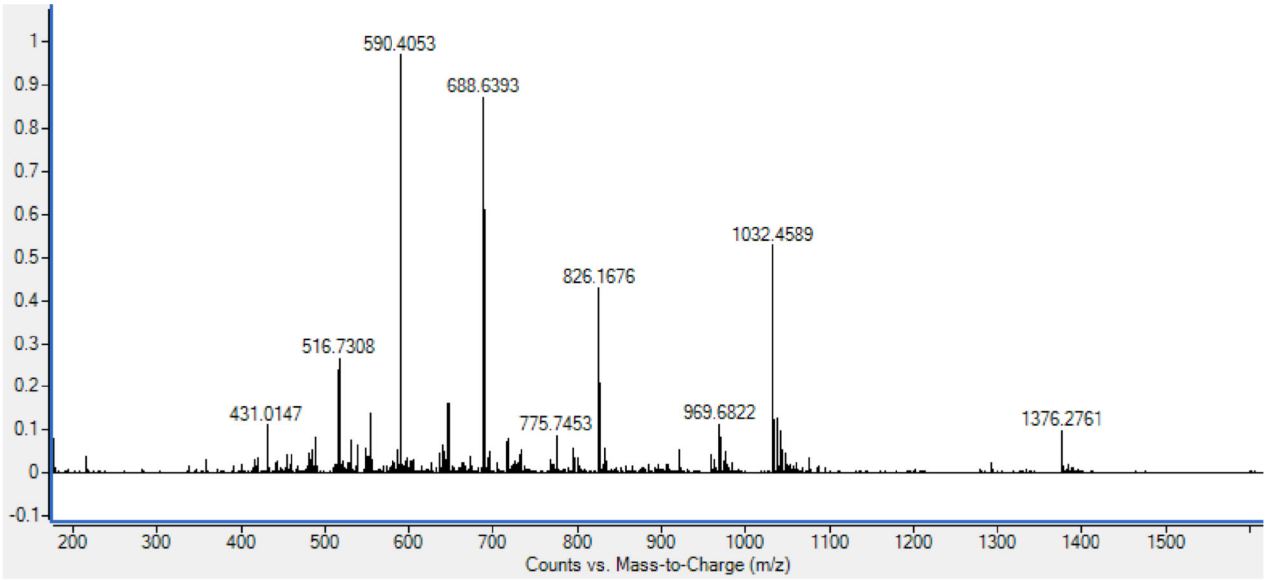

Sample: 18mer in **Figure 6**
Sequence: CGTCGACCGAGAGGTTCT-KKK-CONH2.
Synthesis method: Automated flow synthesis.
Resin: 10 mg Rink Amide resin (0.49 mmol/g).
Tiny Tides in-line synthetic data at UV absorbance 310 nm.

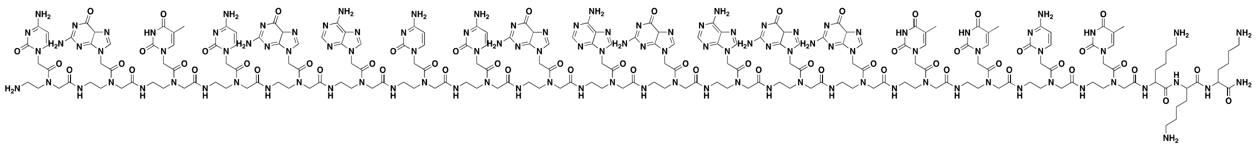

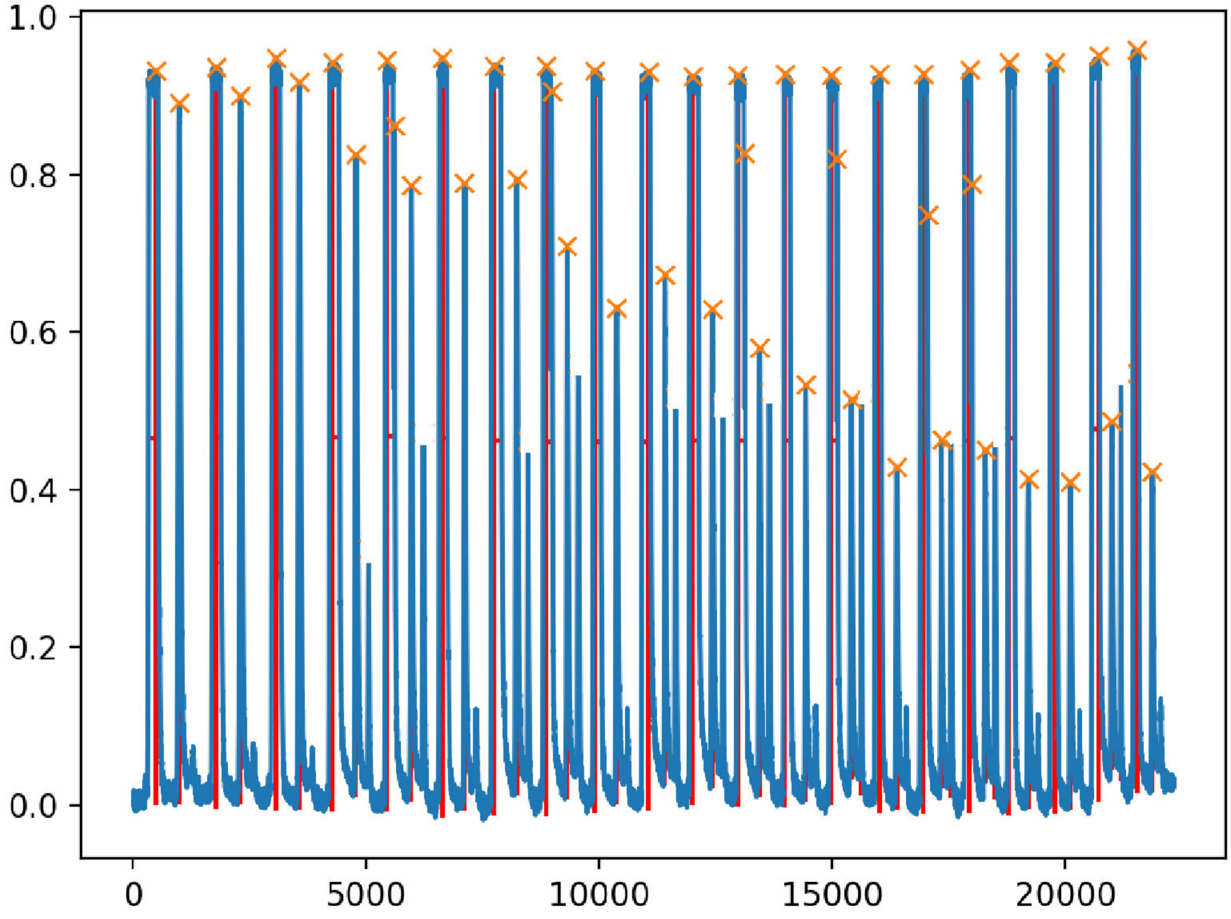

18MER	**1**	**2**	**3**	**4**	**5**	**6**	**7**	**8**	**9**
Predicted	0.971	0.941	0.878	0.905	0.854	0.780	0.798	0.768	0.731
Synthesized	0.970	0.953	0.845	0.882	0.865	0.779	0.817	0.801	0.785
	**10**	**11**	**12**	**13**	**14**	**15**	**16**	**17**	**18**
Predicted	0.657	0.683	0.623	0.648	0.640	0.588	0.578	0.605	0.546
Synthesized	0.699	0.733	0.647	0.680	0.665	0.557	0.537	0.574	0.486

HPLC method: see ***Section S1.2***
Crude purity: 29%

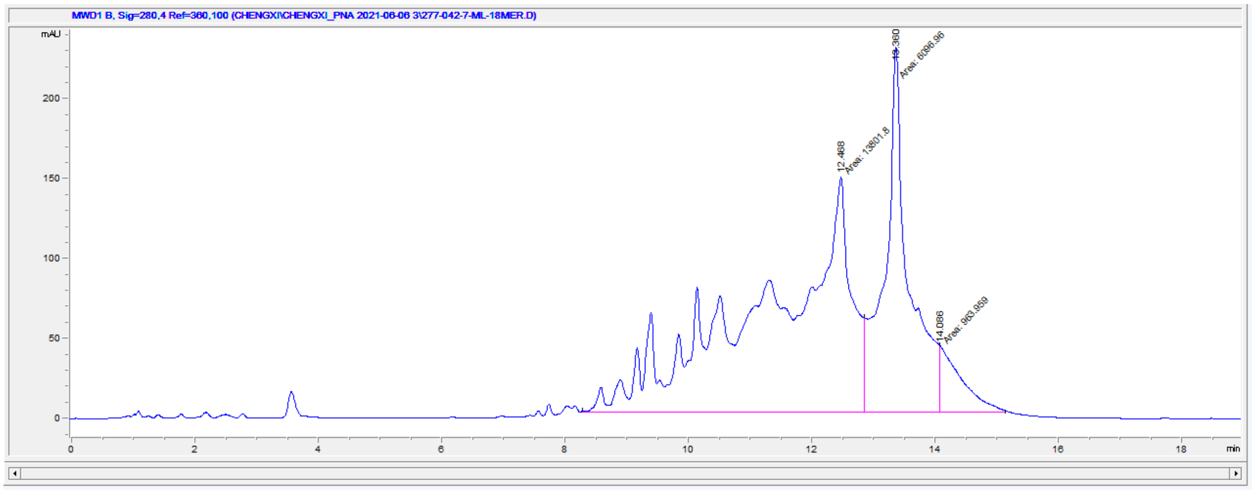

#	Time	Area	Height	Width	Area%	Symmetry
1	12.468	13801.8	147	1.5644	66.155	6.224
2	13.36	6097	228.2	0.4454	29.224	0.81
3	14.086	964	41.1	0.3912	4.620	0

LC-MS method: see ***Section S1.3***
Calculated: 5293.21 Da
Observed: 5293.25 Da

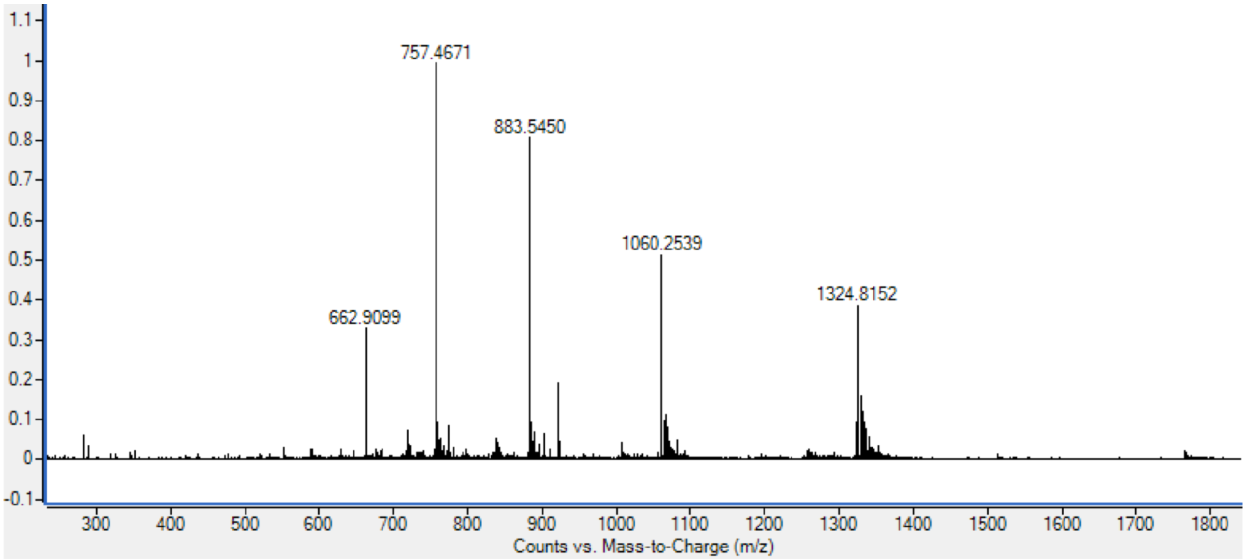

4. LC-MS and HPLC data for anti-exon 44 PNA samples

(I) Easy sequence, score: 0.71
PNA sequence: CTGGAGTGTTTACTTACT-KKK-$CONH_2$
Calculated mass: 5276.25 Da
Observed mass: 5276.43 Da

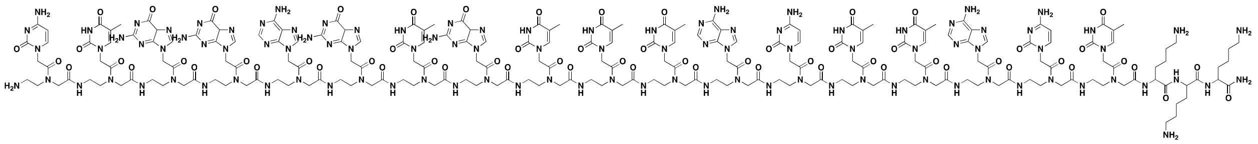

Crude LC-MS data:

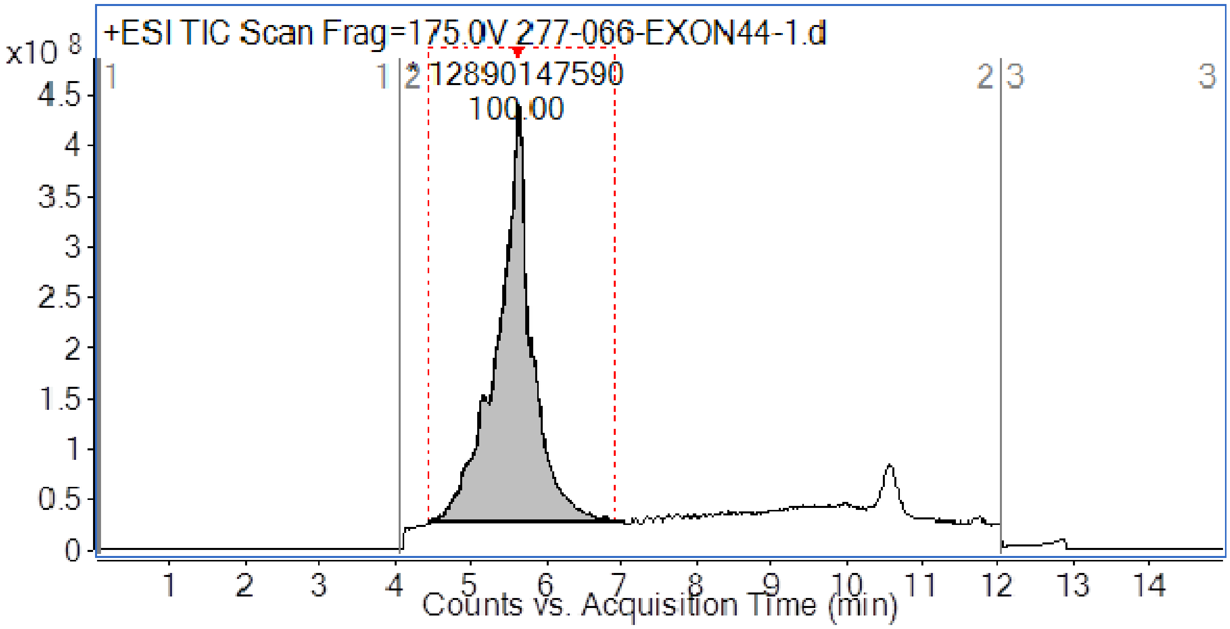

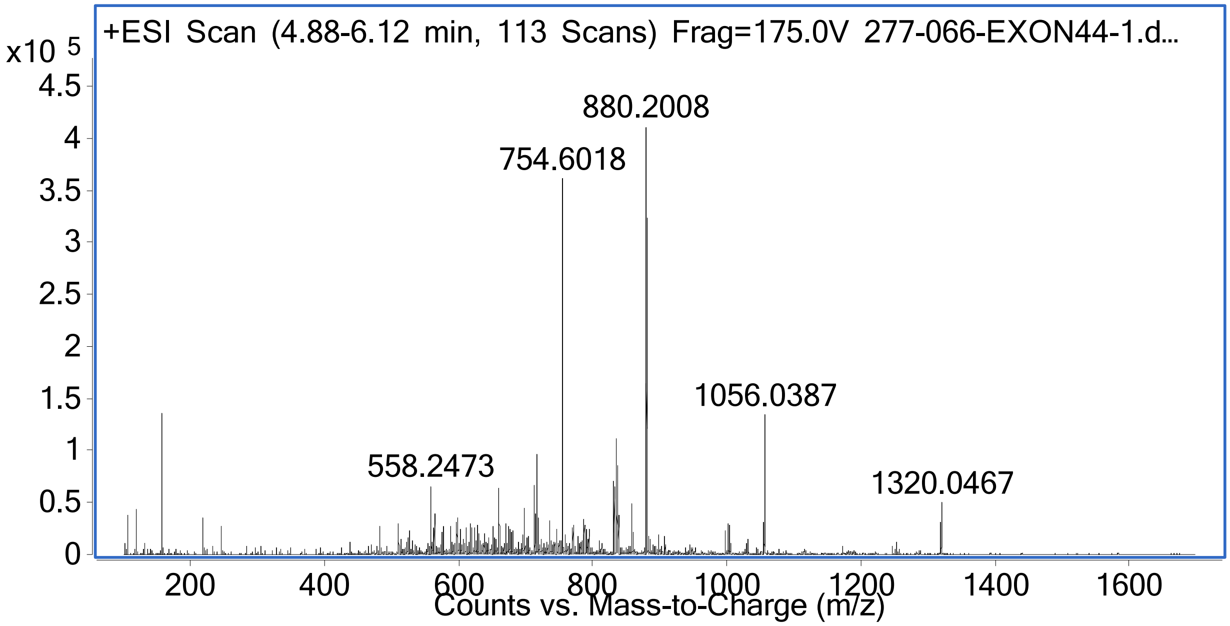
+ESI Scan (4.88-6.12 min, 113 Scans) Frag=175.0V 277-066-EXON44-1.d...
x10 5
880.2008
754.6018
1056.0387
558.2473
1320.0467
Counts vs. Mass-to-Charge (m/z)

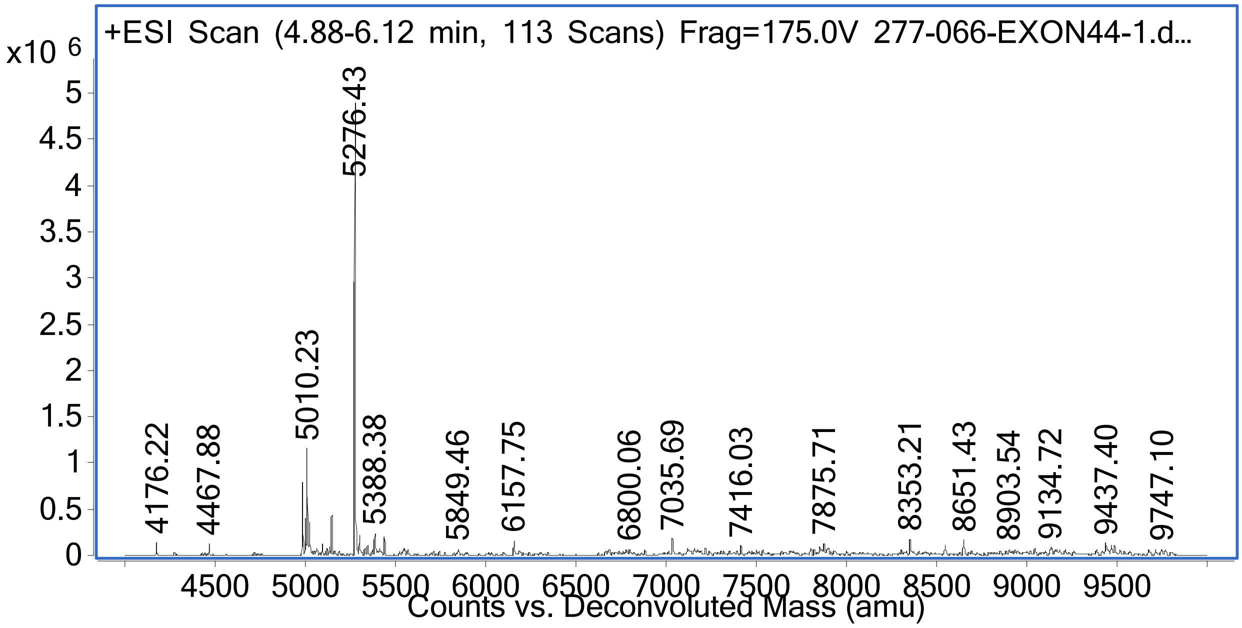
+ESI Scan (4.88-6.12 min, 113 Scans) Frag=175.0V 277-066-EXON44-1.d...
x10 6
5276.43
5010.23
4176.22
4467.88
5388.38
5849.46
6157.75
6800.06
7035.69
7416.03
7875.71
8353.21
8651.43
8903.54
9134.72
9437.40
9747.10
Counts vs. Deconvoluted Mass (amu)

Pure HPLC trace:

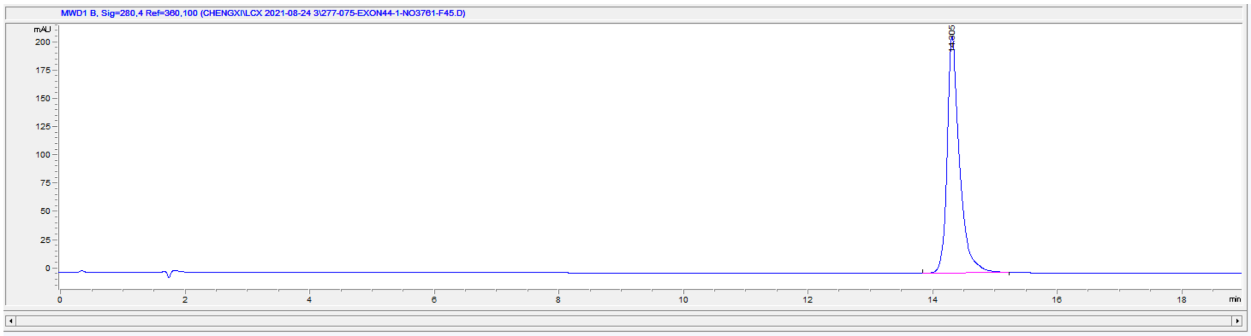

#	Time	Area	Height	Width	Area%	Symmetry
1	14.305	2915.8	209.1	0.2027	100.000	0.592

Pure LC-MS data:

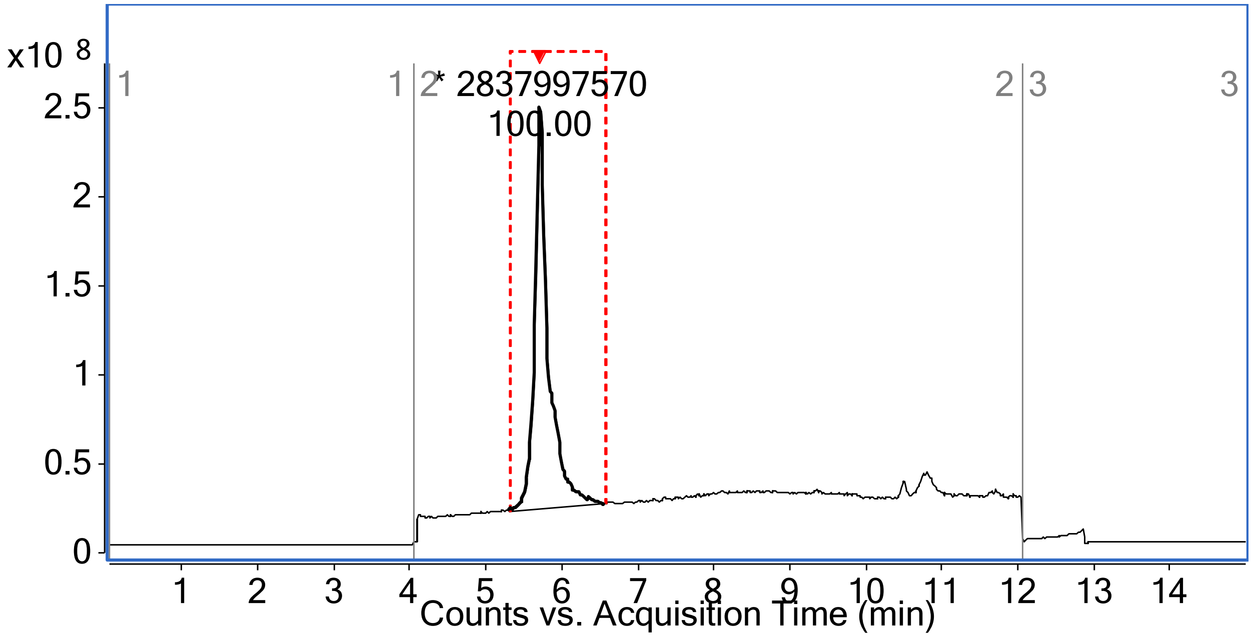

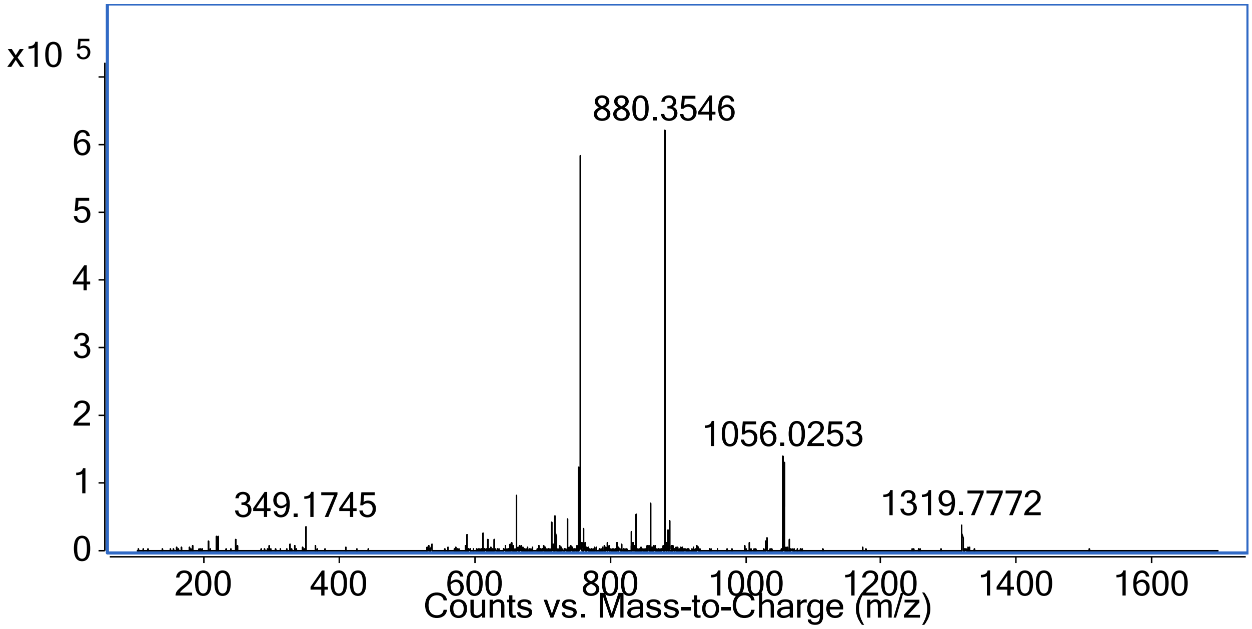
x10 5
880.3546
1056.0253
349.1745
1319.7772
0
1
2
3
4
5
6
200
400
600
800
1000
1200
1400
1600
Counts vs. Mass-to-Charge (m/z)

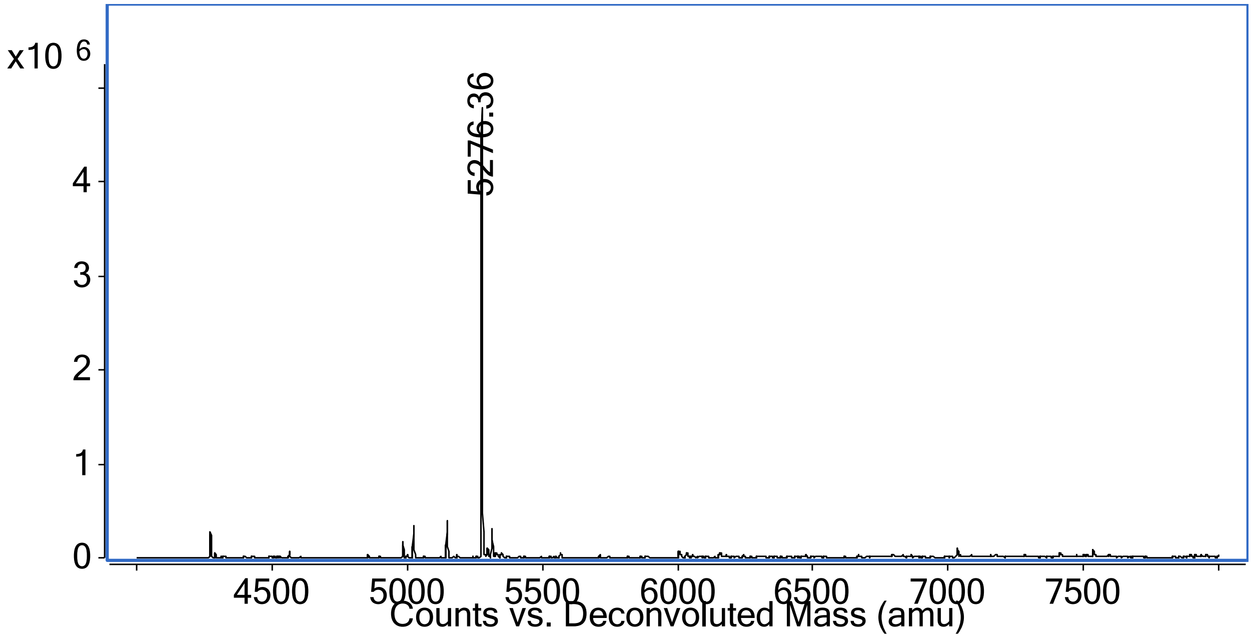
x10 6
5276.36
0
1
2
3
4
4500
5000
5500
6000
6500
7000
7500
Counts vs. Deconvoluted Mass (amu)

(II) Medium sequence, score: 0.59

PNA sequence: TTAAATTTATTTATGTAT-KKK-$CONH_2$

Calculated mass: 5273.29 Da

Observed mass: 5273.54 Da

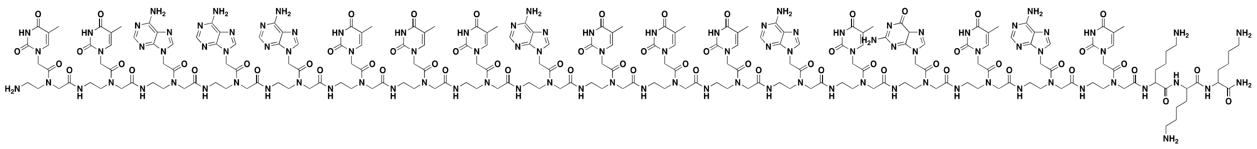

Crude LC-MS data:

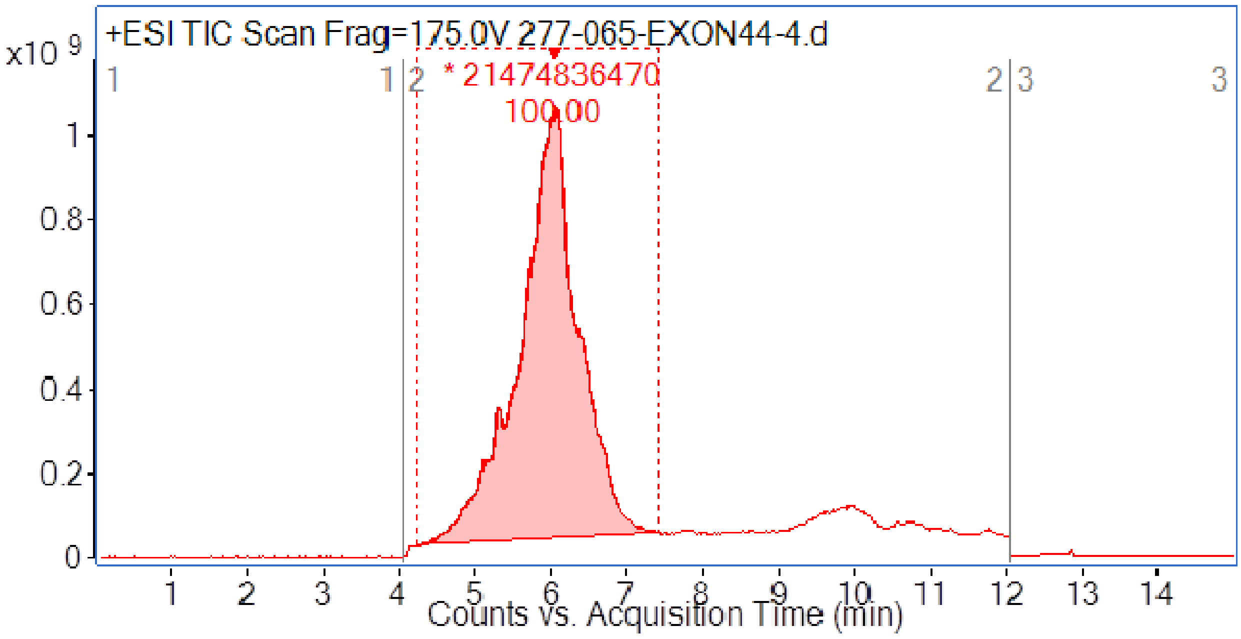

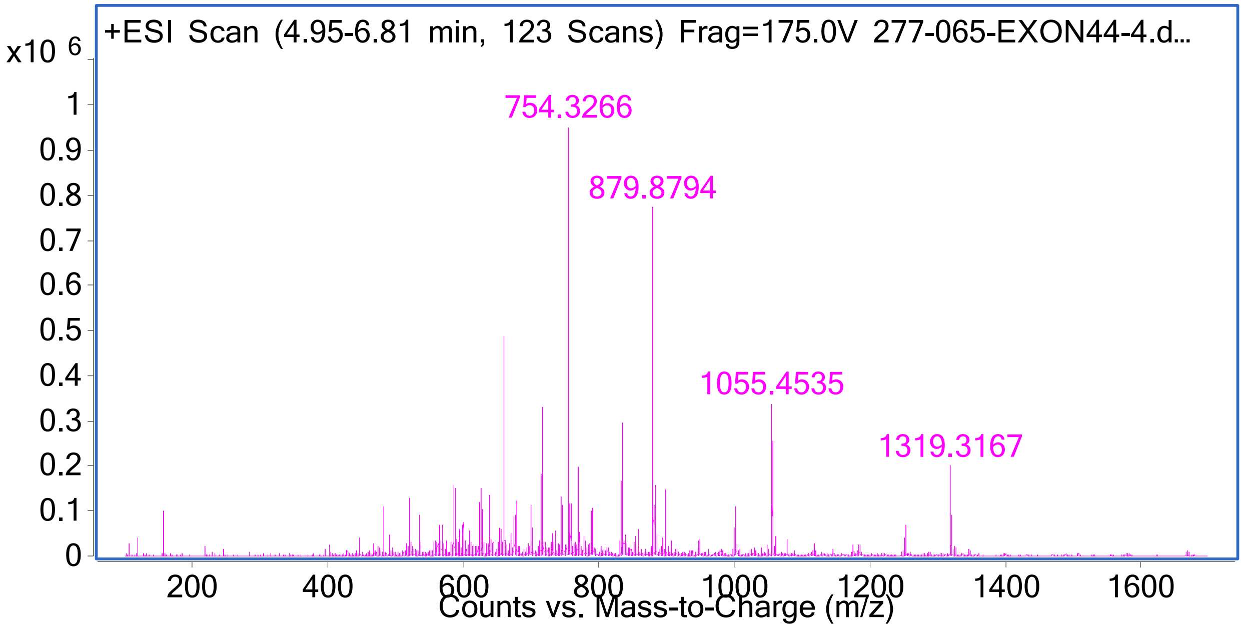

+ESI Scan (4.95-6.81 min, 123 Scans) Frag=175.0V 277-065-EXON44-4.d...
x10 6
1
0.9
0.8
0.7
0.6
0.5
0.4
0.3
0.2
0.1
0
754.3266
879.8794
1055.4535
1319.3167
200
400
600
800
1000
1200
1400
1600
Counts vs. Mass-to-Charge (m/z)

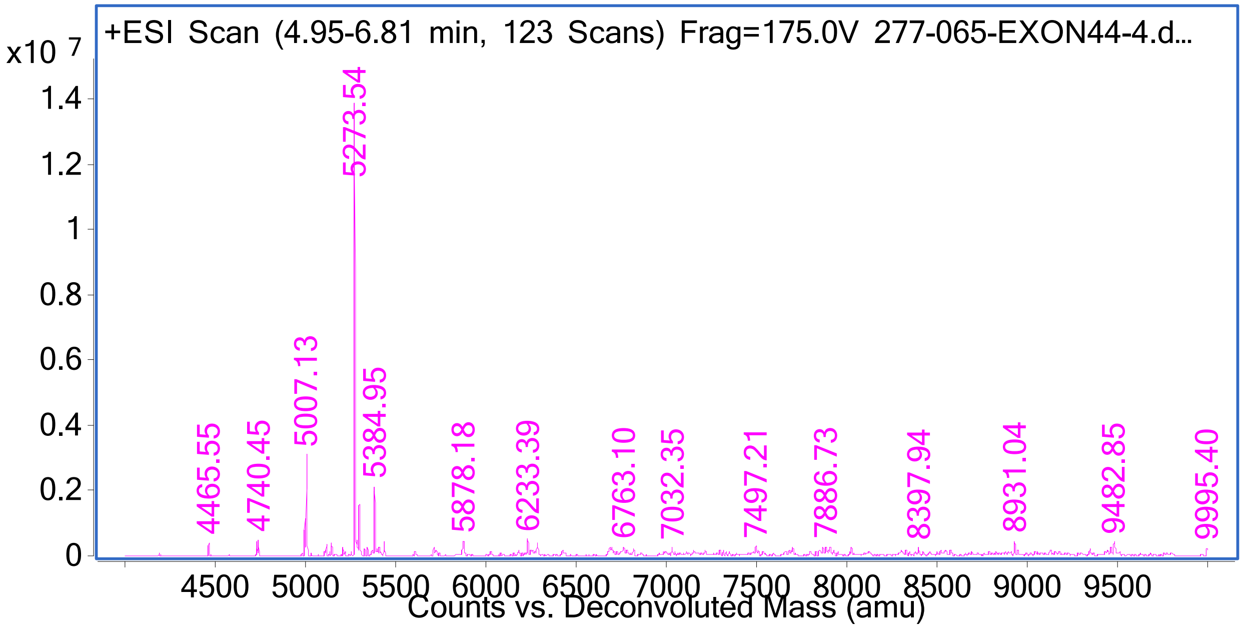

+ESI Scan (4.95-6.81 min, 123 Scans) Frag=175.0V 277-065-EXON44-4.d...
x10 7
1.4
1.2
1
0.8
0.6
0.4
0.2
0
5273.54
5007.13
5384.95
4465.55
4740.45
5878.18
6233.39
6763.10
7032.35
7497.21
7886.73
8397.94
8931.04
9482.85
9995.40
4500
5000
5500
6000
6500
7000
7500
8000
8500
9000
9500
Counts vs. Deconvoluted Mass (amu)

Pure HPLC trace:

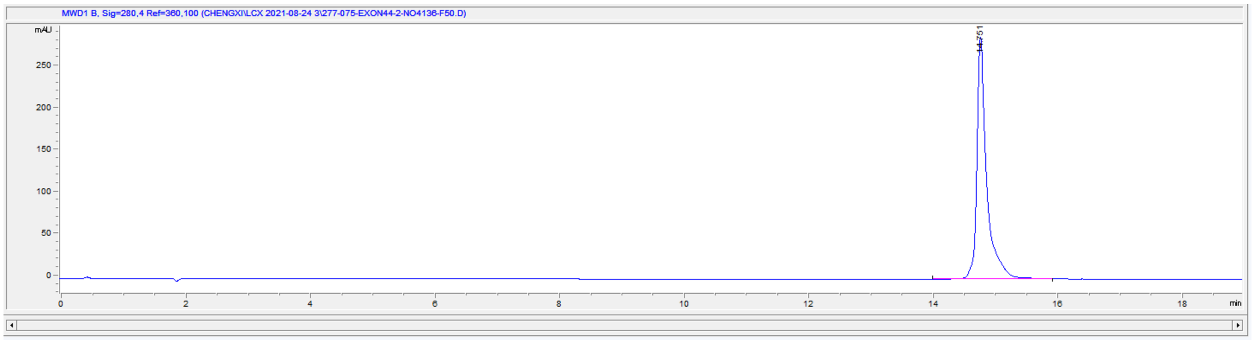

#	Time	Area	Height	Width	Area%	Symmetry
1	14.751	3187.7	288.4	0.157	100.000	0.519

Pure LC-MS data:

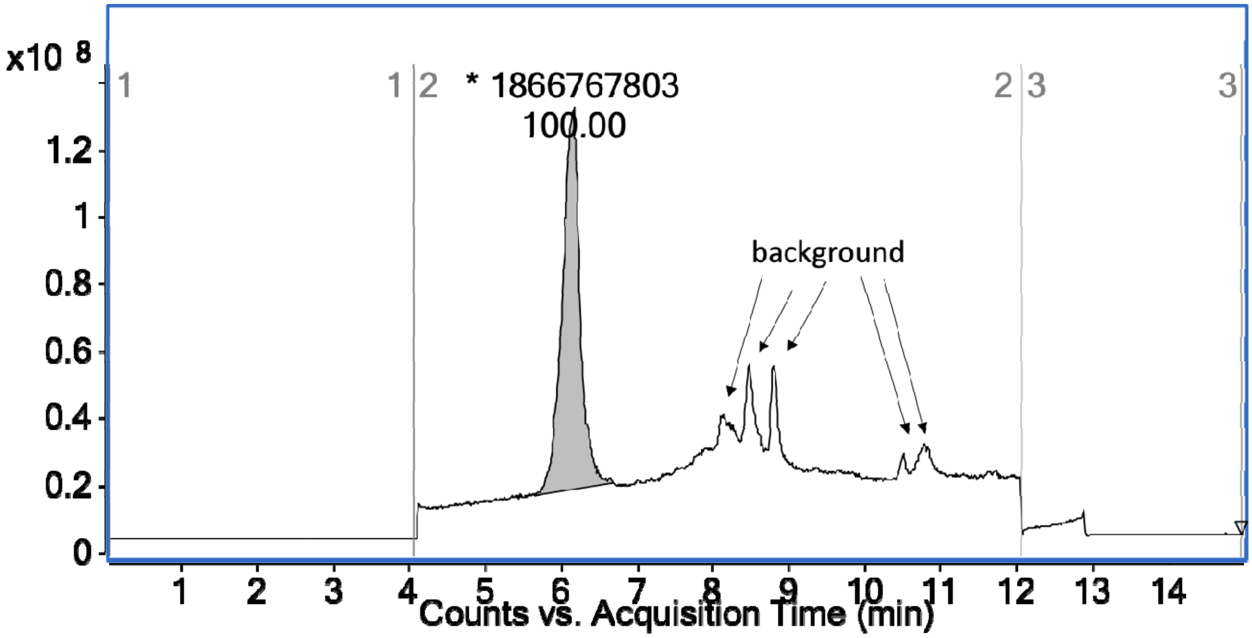

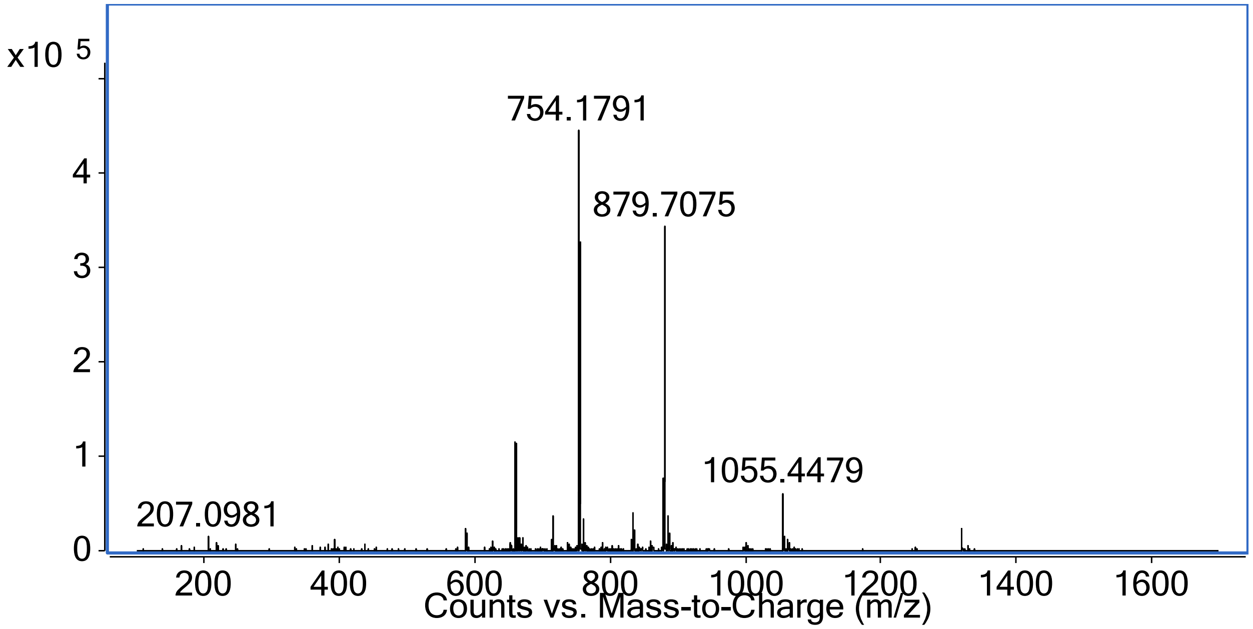
x10 5
754.1791
879.7075
1055.4479
207.0981
4
3
2
1
0
200
400
600
800
1000
1200
1400
1600
Counts vs. Mass-to-Charge (m/z)

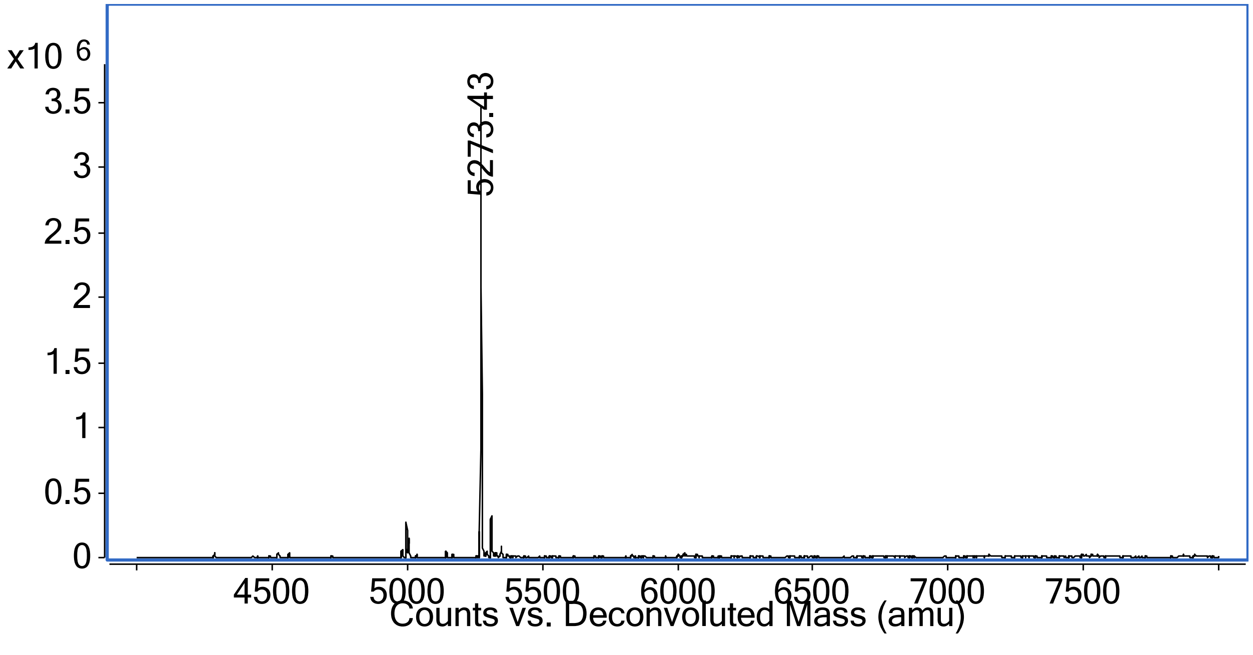
x10 6
5273.43
3.5
3
2.5
2
1.5
1
0.5
0
4500
5000
5500
6000
6500
7000
7500
Counts vs. Deconvoluted Mass (amu)

(III) Difficult sequence, score: 0.32

PNA sequence: AAAGCAAAAATGCAAGCA-KKK-CONH2

Calculated mass: 5323.36 Da

Observed mass: 5323.59 Da (trace amount)

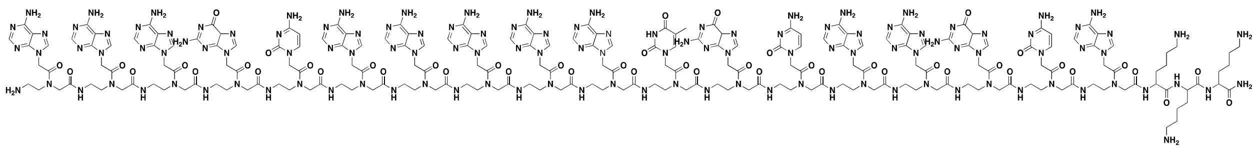

Crude LC-MS data:

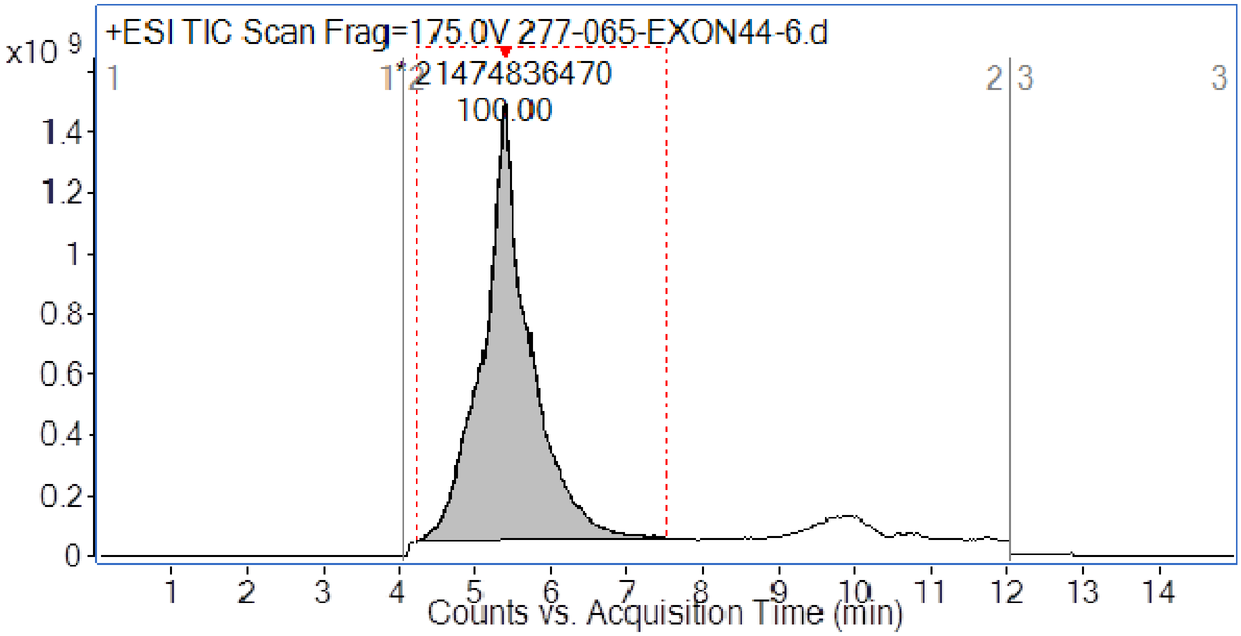

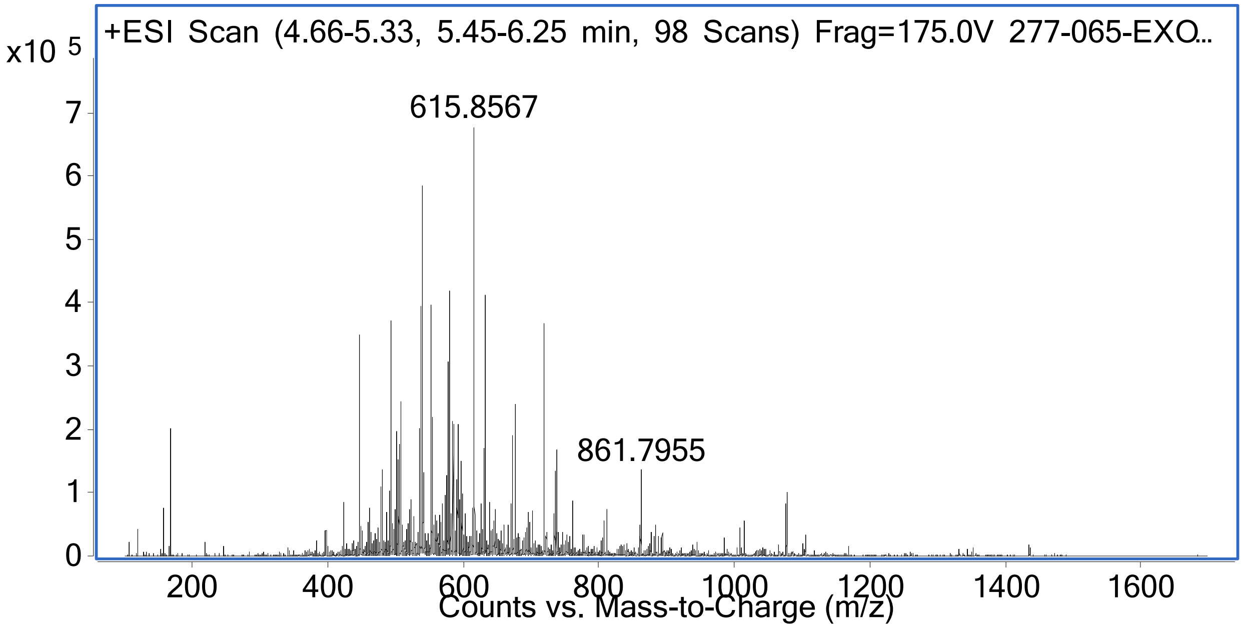

+ESI Scan (4.66-5.33, 5.45-6.25 min, 98 Scans) Frag=175.0V 277-065-EXO...
x10 5
615.8567
861.7955
Counts vs. Mass-to-Charge (m/z)

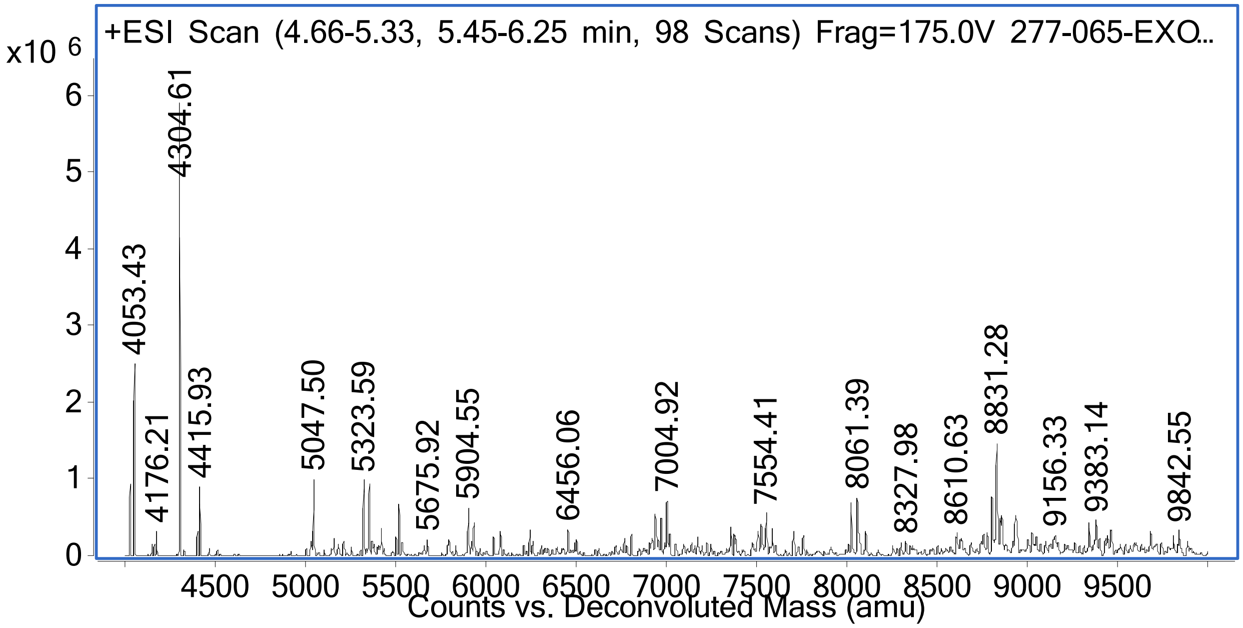

+ESI Scan (4.66-5.33, 5.45-6.25 min, 98 Scans) Frag=175.0V 277-065-EXO...
x10 6
4053.43
4176.21
4304.61
4415.93
5047.50
5323.59
5675.92
5904.55
6456.06
7004.92
7554.41
8061.39
8327.98
8610.63
8831.28
9156.33
9383.14
9842.55
Counts vs. Deconvoluted Mass (amu)

5. Analysis of features importance

5.1 Analysis of features using *n-grams* representation approach

We analyzed the features in the raw dataset weighted by the synthetic yield for the pre-synthesized sequences. All the features, length, 1-mers, and 2-mers, were used for the analysis. Each unique deprotection step, $d \in \{1, 2, \ldots, 240\}$, had associated independent features set, $(length_d, A_d, \ldots, GG_d)$, and the averaged synthetic yield, $area_d$. We multiplied the feature values with synthetic yield of each unique deprotection step and summed across the feature, for instance, $\sum_d length_d \times area_d$. All features were normalized by the sum, to obtain the respective contribution of each feature to the synthetic yield.

We observed that length was a key contributor to synthetic yield, followed by the monomeric nucleic acid composition (Figure S4). In line with common intuition, length was anti-correlated with synthetic yield, i.e., as length of the sequence increases, the synthetic yield decreases. The composition of all monomers, T, C, A, and G, in the same order, in the pre-synthesized sequence was noted to be more important than the dimer composition.

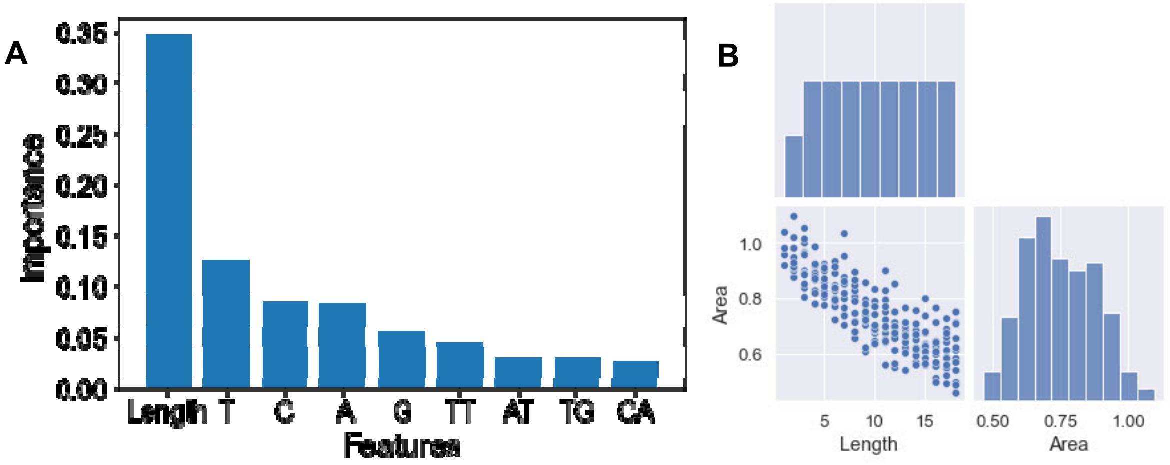

Figure S4. Analysis of features in the raw dataset. A. Individual features important to the synthetic yield are obtained by weighted analysis of different independent features. x-axis represents the features that we selected to train the models, and y-axis represents the importance (or 'contributions') of each feature to the model performances. **B.** Synthetic yield of the PNA sequences is anti-correlated with pre-synthesized sequence length. The dotted plot on the bottom left represents an anti-correlation trend between the length of PNAs (on the x-axis, labelled as 'Length') and the synthetic yield (on the y-axis, labelled as 'Area'). The histogram subpanel on the bottom right shows the summary of the synthetic yield for all sampled PNA sequences. The histogram subpanel on top indicates the length distribution of all analyzed PNA samples.

5.2 Analysis of features using *Ridge* model

Data mining over the training data set informs the feature importance for model performance. As depicted in Figure S5, the relative feature importance contributing to the model prediction was summarized with the Ridge. A higher feature weight indicates a larger contribution to the model prediction. In line with the common intuition, the PNA chain length was ranked as a top important feature by the model (Figure S5). In addition, besides the sequence length, we observed that four PNA monomers, *i.e.,* guanine (G), thymine (T), cytosine (C), and adenine (A), contribute significantly to the model performance. Overall, chain length and four monomers play a more important role than any of the 16 possible dimer permutations with respect to our model performance, and this observation is consistent with a raw data analysis using *n-grams* representation approach (supporting figure S4).

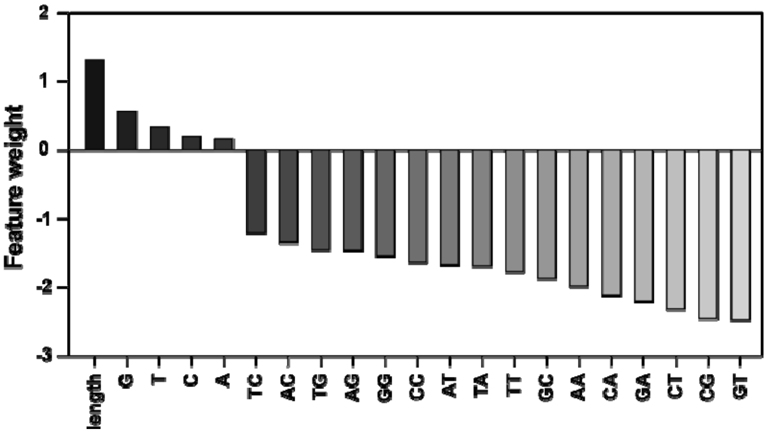

Figure S5. Ridge informs the relative importance of the input 21 features to ML model performance. A higher weight indicates a larger contribution to the model performance.

6. Hyperparameters and optimized hyperparameters

Table S1. Hyperparameters and optimized hyperparameters for regression model architectures. The hyperparameters follow a notation – parameter, datatype, values. Datatypes are categorical, integer and real. For categorical datatype, the list of hyperparameters is noted, and for integer and real datatypes, minimum and maximum values are noted.

Model Architecture [a]	Hyperparameters	Optimized parameters
Linear	'fit_intercept': Categorical([True, False]), 'normalize': Categorical([True, False])	('fit_intercept', False), ('norm alize', False)
Ridge	'fit_intercept': Categorical([True, False]), 'normalize': Categorical([True, False]),	('alpha', 0.004937785846238 9115), ('fit_intercept', True),

	'alpha': Real(1e-3, 1e+3, 'log-uniform'), 'solver': Categorical(['svd', 'cholesky', 'lsqr', 'sparse_cg', 'sag', 'saga'])	('normalize', True), ('solver', 'l sqr')
Lasso	'fit_intercept': Categorical([True, False]), 'normalize': Categorical([True, False]), 'alpha': Real(1e-3, 1e+3, 'log-uniform'), 'precompute': Categorical([True, False]), 'selection': Categorical(['cyclic', 'random'])	('alpha', 0.002318473361504 6775), ('fit_intercept', True), ('normalize', False), ('precom pute', True), ('selection', 'rand om')
SGD	'loss': Categorical(['squared_loss', 'epsilon_insensitive', 'huber', 'squared_epsilon_insensitive']), 'penalty': Categorical(['l1', 'l2', 'elasticnet']), 'alpha': Real(1e-3, 1e+3, 'log-uniform'), 'l1_ratio': Real(1e-3, 1e-1, prior='log-uniform'), 'fit_intercept': Categorical([True, False]), 'learning_rate': Categorical(['invscaling', 'constant', 'optimal', 'adaptive']), 'epsilon': Real(1e-3, 1e+3, 'log-uniform'), 'eta0': Real(1e-2, 1e+1, prior='log-uniform'), 'power_t': Real(1e-2, 1e+1, prior='log-uniform'), 'average': Categorical([True, False])	('alpha', 0.002318473361504 6775), ('average', True), ('epsi lon', 0.004937785846238911 5), ('eta0', 1.7384713009429 9), ('fit_intercept', True), ('l1_ ratio', 0.00386881786896234 14), ('learning_rate', 'invscali ng'), ('loss', 'huber'), ('penalty ', 'l2'), ('power_t', 0.54438507 8717975)
GP-Matern	'alpha': Real(1e-11, 1e-6, 'log-uniform'), 'n_restarts_optimizer': Integer(0, 10)	('alpha', 3.484912420933394e -07), ('n_restarts_optimizer', 6)
GP-RBF	'alpha': Real(1e-11, 1e-6, 'log-uniform'), 'n_restarts_optimizer': Integer(0, 10)	('alpha', 3.484912420933394e -07), ('n_restarts_optimizer', 6)
SVR	'kernel': Categorical(['linear', 'poly', 'rbf', 'sigmoid']), 'degree': Integer(1, 6), 'gamma': Real(1e-6, 1e+1, 'log-uniform'), 'C': Real(1e-2, 1e+1, 'log-uniform'), 'epsilon': Real(1e-3, 1e+1, 'log-uniform'), 'shrinking': Categorical([True, False])	('C', 2.0660514717495357), (' degree', 2), ('epsilon', 0.17964 60960116844), ('gamma', 0.0 04127871450145202), ('kerne l', 'linear'), ('shrinking', False)
RF	'criterion': Categorical(['mse', 'mae']), 'n_estimators': Integer(10, 1000,	('criterion', 'mae'), ('max_dept h', 10), ('n_estimators', 630)

	prior='log-uniform'),<br>'max_depth': Integer(1, 10)	
GB	'loss': Categorical(['ls', 'lad', 'huber', 'quantile']),<br>'learning_rate': Real(1e-2, 1, prior='log-uniform'),<br>'n_estimators': Integer(10, 1000, prior='log-uniform'),<br>'criterion': Categorical(['friedman_mse', 'mse', 'mae',]),<br>'max_depth': Integer(1, 10)	('criterion', 'friedman_mse'), (' learning_rate', 0.3511150694 3935533), ('loss', 'ls'), ('max_ depth', 2), ('n_estimators', 19 7)]
kNN	'weights': Categorical(['uniform', 'distance']),<br>'leaf_size': Integer(10, 100, prior='log-uniform'),<br>'n_neighbors': Integer(2, 20, prior='log-uniform'),<br>'algorithm': Categorical(['auto', 'ball_tree', 'kd_tree', 'brute']),<br>'p': Integer(1, 5)	('algorithm', 'auto'), ('leaf_size ', 80), ('n_neighbors', 12), ('p', 1), ('weights', 'distance')

[a]Abbreviations: SGD – Stochastic gradient descent, GP – Gaussian process, SVR – Support vector regression, RF – Random Forest, GB – gradient boosting, kNN – k-nearest neighbors.

7. ML model architecture performance on validation and test data sets

Table S2. Metrics of performance for different ML model architectures on validation and test data sets. Ridge model performs the best on both validation and test data sets.[a]

Model Architectures	Validation				Test			
	**uRMSE**	**R^2**	**PC**	**SC**	**uRMSE**	**R^2**	**PC**	**SC**
Linear	0.071	0.895	0.949	0.946	0.078	0.932	0.966	0.952
**Ridge**	0.071	0.896	0.949	0.946	**0.075**	**0.934**	**0.967**	**0.954**
Lasso	0.075	0.890	0.946	0.940	0.076	0.933	0.966	0.950
SGD	0.471	0.307	0.923	0.925	0.868	0.237	0.936	0.934
GP-Matern	0.000	1.000	1.000	1.000	0.080	0.929	0.965	0.948
GP-RBF	0.000	1.000	1.000	1.000	0.115	0.899	0.949	0.932
SVR	0.065	0.904	0.955	0.956	0.076	0.933	0.966	0.953
RF	0.019	0.971	0.986	0.987	0.140	0.877	0.938	0.915
GB	0.014	0.980	0.990	0.985	0.126	0.889	0.944	0.918
kNN	0.000	1.000	1.000	1.000	0.137	0.879	0.940	0.915

[a]Abbreviations: uRMSE – unitless/relative root-mean-squared error, PC – Pearson's correlation, SC – Spearman's rank correlation coefficient.

8. References

1. C. Li, A. J. Callahan, M. D. Simon, K. A. Totaro, A. J. Mijalis, K.-S. Phadke, G. Zhang, N. Hartrampf, C. K. Schissel, M. Zhou, H. Zong, G. J. Hanson, A. Loas, N. L. B. Pohl, D. E. Verhoeven and B. L. Pentelute, *Nat. Commun.*, 2021, **12**, 4396.

2. C. Li, A. J. Callahan, K.-S. Phadke, B. Bellaire, C. E. Farquhar, G. Zhang, C. K. Schissel, A. J. Mijalis, N. Hartrampf, A. Loas, D. E. Verhoeven and B. L. Pentelute, *ChemRxiv*, 2021, ACS Cent. Sci., https://doi.org/10.1021/acscentsci.1c01019.

9. Synthetically facile antisense PNA sequences for diseases predicted by ML

9.1 Top 100 antisense PNA sequences for exon 44 of human dystrophin gene

Serial No.	PNA sequences	Predicted scores
4893	GTGGTTACTACAGTAAGT	0.810072944
2105	CATAGTAGTAGCAGTGGT	0.80941179
4896	GTTACTACAGTAAGTTAT	0.809396321
2102	GGACATAGTAGTAGCAGT	0.798569478
2584	CCTAGCCTTAGGTACTTT	0.782730972
10546	GTACTCCCTAGTTAACCT	0.779360373
4895	GGTTACTACAGTAAGTTA	0.774798145
2098	GCTAGGACATAGTAGTAG	0.763334142
10540	GTAAAAGTACTCCCTAGT	0.757872436
12677	GTAATAAGTTCTAGTGTT	0.757802998
9762	GGGTTTTGCCTCAGTAGT	0.756369174
4900	CTACAGTAAGTTATGTTT	0.755060122
2099	CTAGGACATAGTAGTAGC	0.752810424
3577	GTAAGTATCCATTAGAGT	0.750250474
583	GCTAGTTACTCTTTGGGT	0.74895698
2582	TACCTAGCCTTAGGTACT	0.748920243
12674	GGAGTAATAAGTTCTAGT	0.748861336
2631	GTTACCTACTTATCTTTT	0.747406958
584	CTAGTTACTCTTTGGGTT	0.745700212
2620	GTATTAAGAGAGTTACCT	0.741304967
2589	CCTTAGGTACTTTGTTAC	0.73973575

14227	GTATTCTCTAACTGTAGT	0.739031113
2339	GTGACAGTATTAAGGGGT	0.737833234
1455	GGAGGTGGTAGCAAGGTT	0.737513871
5762	CTTACCTCCTATAGAGTT	0.736330349
9684	GCTAGTAATACCACCTGT	0.736330016
2266	GTAGTCGTCAGTATCACT	0.735678494
1328	GTGTGGTTCAGGGCCAGT	0.735095841
2629	GAGTTACCTACTTATCTT	0.73497002
9685	CTAGTAATACCACCTGTT	0.733073248
4903	CAGTAAGTTATGTTTTTT	0.732873461
14047	GTGATAGTTCTAGAGGTT	0.732770718
7465	GTAAATAAGTCTTAGTTT	0.73259452
2103	GACATAGTAGTAGCAGTG	0.732412129
2579	TATTACCTAGCCTTAGGT	0.732210364
4800	GTAGTCTCTTACTCTCTT	0.731911907
14049	GATAGTTCTAGAGGTTTT	0.731441655
3766	GTGTTTACTTACTGCCCT	0.730746877
5842	GTAGCAGATACTGTCAGT	0.730236242
4206	GTACCGAGGTATATTGGT	0.729639593
3768	GTTTACTTACTGCCCTTT	0.729417813
10350	GTGAGTAGTGGGGCACTT	0.727343097
2276	GTATCACTACTATGGTTT	0.727326388
2093	GTTATGCTAGGACATAGT	0.727318915
2233	CAACAGCTAGGGTAGCCT	0.727058143
12312	CAGAGTTTAGTTTCAAGT	0.726849126
2588	GCCTTAGGTACTTTGTTA	0.726567312
2109	GTAGTAGCAGTGGTCAGA	0.726192977
7463	CAGTAAATAAGTCTTAGT	0.724430753
2586	TAGCCTTAGGTACTTTGT	0.723778627
2355	GTGGGAGCTTTAGGAGGT	0.723641512
5515	GTTAGGGTCCCAGTGCAG	0.723305955
2256	GCCCTGTGTGGTAGTCGT	0.721477464
4898	TACTACAGTAAGTTATGT	0.721249393
2261	GTGTGGTAGTCGTCAGTA	0.72107491
4894	TGGTTACTACAGTAAGTT	0.720596952
2583	ACCTAGCCTTAGGTACTT	0.719681787
2274	CAGTATCACTACTATGGT	0.719162622
14056	CTAGAGGTTTTTTCCCCT	0.717775266
2585	CTAGCCTTAGGTACTTTG	0.717113944

14061	GGTTTTTTCCCCTTATTT	0.716426015
1356	GGACAGCATTTAGTAGCT	0.715606071
10553	CTAGTTAACCTTCTACAT	0.715363777
4518	GATAGGGTAGAGCCCTCT	0.714612198
2228	GGGTGCAACAGCTAGGGT	0.714488899
4516	GAGATAGGGTAGAGCCCT	0.714271648
6412	GTTAAACTATAAGTTTTT	0.714135995
5097	GGTACCACACTCCCAGTA	0.714024842
2590	CTTAGGTACTTTGTTACA	0.713786449
2594	GGTACTTTGTTACAGCAG	0.713641714
5385	CCTTATGCTAGTGTATGT	0.713635134
3581	GTATCCATTAGAGTTCCT	0.713575947
2243	GGTAGCCTCCCAAGCCCT	0.713250597
2253	CAAGCCCTGTGTGGTAGT	0.713225351
4523	GGTAGAGCCCTCTGGTAG	0.712980919
2236	CAGCTAGGGTAGCCTCCC	0.712942095
5798	CCATGTTAGGTGTATCCT	0.712113139
7520	GTTATAATAGGGGCTTAC	0.711915603
14062	GTTTTTTCCCCTTATTTT	0.711601675
10549	CTCCCTAGTTAACCTTCT	0.711598722
13125	CAGGAACTTAGTACAAGT	0.711522188
3761	CTGGAGTGTTTACTTACT	0.711309473
3647	CCAAGTCAGACTAGGTAT	0.710879354
14680	TTTTAGCTACTAGGAGGT	0.710552947
3754	GTACTTTCTGGAGTGTTT	0.710505027
7074	CTTATTTTTTCCTAGTCT	0.71020022
5802	GTTAGGTGTATCCTAATA	0.709580246
13468	GGGCTTACAGTCTACCAG	0.709324622
4880	GCAGTTCTTTACCGTGGT	0.709295241
2581	TTACCTAGCCTTAGGTAC	0.709213053
10102	CAGTTATTATATCTGTAT	0.70834526
5395	GTGTATGTCTTCTAGTTA	0.708107196
3661	GTATCCCAGAGGTGTAAT	0.707946324
2112	GTAGCAGTGGTCAGACAT	0.707633845
2263	GTGGTAGTCGTCAGTATC	0.707364785
8966	GTGTGGGGGTGGGCACCT	0.707124567
8968	GTGGGGGTGGGCACCTGT	0.707124567
2401	GAGTGGGTTTGGTTCCCT	0.706535085
4889	TACCGTGGTTACTACAGT	0.706510793

10551	CCCTAGTTAACCTTCTAC	0.706174581

9.2 Top 100 antisense PNA sequences for SARS-CoV-2

Serial No.	PNA sequences	Predicted scores
23165	GTAACTATGTTAGTAGTT	0.815965461
1281	GCCCAGTTCCTAGGTAGT	0.81152538
21921	CTAGTAACAGTATAGGTT	0.811296971
17018	GTATAGATAGTACCAGTT	0.808412937
1283	CCAGTTCCTAGGTAGTAG	0.794064093
23173	GTTAGTAGTTGTACTAAC	0.792968151
26920	GTAGTATGTAGCCATACT	0.791957105
25718	CCAGCCTTTTTAGTAGGT	0.785859147
19772	GTAGTTGTACCACAAGTT	0.782105885
28951	GTATACACCCCTCTTAGT	0.781289832
24723	CAGTTGTGTGGTAGTACT	0.780945223
29145	GTTTAGTGTTCCAGTTTT	0.776942328
19765	GTTAAGTGTAGTTGTACC	0.776394573
14035	GAGGTCCTTTAGTAAGGT	0.776372329
1282	CCCAGTTCCTAGGTAGTA	0.774322877
22779	GTATAGAACCAGTACAGT	0.773816664
24476	GTCTTATTACAGTAGGCT	0.773595193
22784	GAACCAGTACAGTAGGTT	0.773164223
27320	GCTTCACTAGTAGGTTGT	0.769447476
10449	GTAGCAGACTTTAGTGGT	0.769106925
12801	CTCTTACCAGTACCAGGT	0.768189605
23169	CTATGTTAGTAGTTGTAC	0.767988148
24144	GGTAATTACCAGTGTACT	0.767092171
27321	CTTCACTAGTAGGTTGTT	0.766190708
13849	GTTTAGTAAGTGGGTAAG	0.764150784
15282	GTAGTGCGTTTATCTAGT	0.763645312
4020	GTTACACTATTGTAAGGT	0.762808152
11099	GCTTTGTAGGTTACCTGT	0.76184211
25721	GCCTTTTTAGTAGGTATA	0.761269941
17114	GTTGTGTTGTAGTAAGCT	0.760738312
12768	GGGTAGTAGAGAGCTAGG	0.760309179
13279	GTCTTTCAGTACAGGTGT	0.760146185
27318	CAGCTTCACTAGTAGGTT	0.759954645
14041	CTTTAGTAAGGTCAGTCT	0.759440754

18146	GGTGGGAGTAGTCCCTGT	0.759276658
26477	GGTTTTACCTTTTTAGCT	0.759183983
22037	CCTTTAGTGTTATTAGCT	0.758241797
19960	GTAGCTAGTTGTATCCAT	0.757674057
26352	GTCCATTAGTAGCTATGT	0.757674057
9699	GTAAAGTAAGTTTCAGGT	0.757256914
12808	CAGTACCAGGTGGTCCCT	0.756440368
10640	GTTAGATAGCACTCTAGT	0.756333915
6441	GCCAAGTAGGAGTAAGTT	0.754857963
23163	GTGTAACTATGTTAGTAG	0.754632452
26478	GTTTTACCTTTTTAGCTT	0.754359642
1280	GGCCCAGTTCCTAGGTAG	0.753687647
1284	CAGTTCCTAGGTAGTAGA	0.753317901
27326	CTAGTAGGTTGTTCTAAT	0.753028151
13146	GGTCTAGGTTTACCAACT	0.753014279
8830	CATTTTTAGTCTTAGGGT	0.752976105
16577	GTAGGTATTTGTACATAC	0.752457159
23170	TATGTTAGTAGTTGTACT	0.751697948
19775	GTTGTACCACAAGTTACT	0.751550973
19778	GTACCACAAGTTACTTGT	0.751550973
20921	GTGTACTCTATAAGTTTT	0.751127501
23177	GTAGTTGTACTAACAACT	0.750814277
24664	GTATTTCCACTTTTTAGT	0.750535133
25012	GGTAGGATTACTAGTGTA	0.750369311
1368	GGTAGTAGCCAATTTGGT	0.750062929
10301	GTTCCAGAGGTTATAAGT	0.749598033
25062	CTATACCTAGTTGTGTAG	0.749317283
23709	GTGTGTAGTGTTTATAAT	0.749172137
11470	CTGTATTATTAGGTGTAT	0.748545008
12769	GGTAGTAGAGAGCTAGGC	0.748217888
13147	GTCTAGGTTTACCAACTT	0.748189938
12770	GTAGTAGAGAGCTAGGCC	0.747677567
25719	CAGCCTTTTTAGTAGGTA	0.747629393
13277	GAGTCTTTCAGTACAGGT	0.746380183
19543	CTTATACTTAGGTGTCTT	0.746352654
25723	CTTTTTAGTAGGTATAAC	0.746274946
11499	GTAGGTACAGCAACTAGG	0.745999148
11723	CTTAGGTATGCCAGGTAT	0.745940523
19113	GGTCTTGTGTTAGAGGTT	0.745884279

13141	GTGGTGGTCTAGGTTTAC	0.745625029
20543	GTAAACATATTAGTAAGT	0.744927612
16222	GGTAGTTAGAGAAAGTGT	0.744373068
12944	CACTAGTGTAGGTGCACT	0.744154249
11786	GTGTGTAGGTGCCTGTGT	0.743642641
23707	GGGTGTGTAGTGTTTATA	0.743277716
21920	TCTAGTAACAGTATAGGT	0.743203194
19956	CTCTGTAGCTAGTTGTAT	0.743190455
17803	GGCTATAGCTTGTAAGGT	0.742983813
20919	CAGTGTACTCTATAAGTT	0.742963734
28555	GGGTAAGTAACCACAAGT	0.742666673
13087	GTACTTTACTGTTTTTAG	0.741151109
21450	GTATTTGTTTTCGTAGTT	0.741125574
26402	GTAGCCTTATTTAAGGCT	0.741114427
9309	GTATAGTCAATAGTCACT	0.741066929
18892	CCAGTGGTGTGTACCCTT	0.741055707
17924	GTAGTATCTTTAGCTAAG	0.740211014
24813	GTTTTATTTTAGTAACAT	0.739944869
27329	GTAGGTTGTTCTAATGGT	0.739918585
1358	GTAGCTCTTCGGTAGTAG	0.739646919
830	GCTTTAGTGGCAGTACGT	0.739627348
13846	GATGTTTAGTAAGTGGGT	0.739578034
13285	CAGTACAGGTGTTAGCTA	0.739434362
17917	GGCTTCAGTAGTATCTTT	0.739312145
22045	GTTATTAGCTCTCAGGTT	0.739312145
16095	GTGTATTTAGTAAGACGT	0.739092269
16571	CAAGTTGTAGGTATTTGT	0.739026865

9.3 Top 100 antisense PNA sequences for HIV-1

Serial No.	PNA sequences	Predicted scores
6678	GTTTATACTAGGTATGGT	0.802228765
8121	GTACTAGTAGTTCCTGCT	0.796333891
7144	GTAGGTCCTACTAATACT	0.781093457
6199	GTTAGTGGTATTACTTCT	0.779831256
6205	GGTATTACTTCTGTTAGT	0.779831256
8114	CTGAAGGGTACTAGTAGT	0.77422182
8119	GGGTACTAGTAGTTCCTG	0.773542333
3551	GCTACTATTGGTATAGGT	0.772006931

8118	AGGGTACTAGTAGTTCCT	0.768902467
3552	CTACTATTGGTATAGGTT	0.768750163
7162	GTACCTATAGCTTTATGT	0.766642269
7141	GGTGTAGGTCCTACTAAT	0.7650602
8120	GGTACTAGTAGTTCCTGC	0.761451042
4287	GCTAGTTCAGGGTCTACT	0.760846365
33	GCAGTGGGTTCCCTAGTT	0.760653193
9118	GCAGTGGGTTCCCTAGTT	0.760653193
6197	CTGTTAGTGGTATTACTT	0.759778105
4288	CTAGTTCAGGGTCTACTT	0.757589597
8649	GGTTGTAGCTGTCCCAGT	0.757157917
8653	GTAGCTGTCCCAGTATTT	0.756481294
8116	GAAGGGTACTAGTAGTTC	0.754567781
5309	CTACTACAGGTGGCAGGT	0.752473866
39	GGTTCCCTAGTTAGCCAG	0.748863307
9124	GGTTCCCTAGTTAGCCAG	0.748863307
36	GTGGGTTCCCTAGTTAGC	0.74837274
9121	GTGGGTTCCCTAGTTAGC	0.74837274
4282	GGTCTGCTAGTTCAGGGT	0.744813108
5596	GTTACTATGTTTACTTCT	0.744452003
7139	CAGGTGTAGGTCCTACTA	0.741353332
5062	GTATGTATTGTTTTTACT	0.741167576
2204	CAGTACTCCAAGTACTAT	0.740678613
4652	GTATTACTACTGCCCCTT	0.739178613
2979	GCTACTACTATTGGTATT	0.736627679
8006	GGTAGGGCTATACATTCT	0.735979083
2552	GTACTATTATGGTTTTAG	0.734742352
2230	GTACTATTAAACAGTTGT	0.734104397
399	GGTACTAGCTTGTAGCAC	0.7338015
400	GTACTAGCTTGTAGCACC	0.733261179
2844	GGTAGTATCATTATCTAT	0.733222751
448	GCCCTGGTGTGTAGTTCT	0.733172145
7160	CTGTACCTATAGCTTTAT	0.733163667
5058	GTCAGTATGTATTGTTTT	0.732199363
8007	GTAGGGCTATACATTCTT	0.731154743
3169	GGGTTGGGGTCTGTGGGT	0.731030411
2983	CTACTATTGGTATTAGTA	0.729980088
7150	CCTACTAATACTGTACCT	0.729945073
2832	CAACTTATAGCTGGTAGT	0.729662628

38	GGGTTCCCTAGTTAGCCA	0.729662413
9123	GGGTTCCCTAGTTAGCCA	0.729662413
2845	GTAGTATCATTATCTATT	0.728398411
2991	GGTATTAGTATCATTCTT	0.72774597
6217	GTTAGTGCTTTGGTTCCT	0.727559062
7142	GTGTAGGTCCTACTAATA	0.726290125
5233	GGACTACAGTCTACTTGT	0.726122548
7137	GACAGGTGTAGGTCCTAC	0.726078629
5987	GTTTTACATCATTAGTGT	0.725630223
5594	CTGTTACTATGTTTACTT	0.724398853
6351	CACTATAGGCTGTACTGT	0.724316195
3736	GGTACAAGCAGTTTTAGG	0.723812488
34	CAGTGGGTTCCCTAGTTA	0.72345069
9119	CAGTGGGTTCCCTAGTTA	0.72345069
2544	GTTCAGCTGTACTATTAT	0.722835126
7323	CTTTAGTTGCCCCCCTAT	0.7226363
5668	GTTGTGTCAGTTAGGGTG	0.722303044
45	CTAGTTAGCCAGAGAGCT	0.721655077
9130	CTAGTTAGCCAGAGAGCT	0.721655077
8665	GTATTTGTCTACAGCCTT	0.721642363
8593	GGGTTGCTACTGTATTAT	0.720594422
8440	GCACTATAGGGTAATTTT	0.719371716
8003	GCTGGTAGGGCTATACAT	0.7191827
2542	GTGTTCAGCTGTACTATT	0.718687408
8650	GTTGTAGCTGTCCCAGTA	0.718387841
6873	CCATTTAGTACTGTCTTT	0.718385595
8115	TGAAGGGTACTAGTAGTT	0.71822443
445	CTGGCCCTGGTGTGTAGT	0.717943335
3561	GTATAGGTTGCATTACAT	0.717805898
1241	GGGTTGGGAGGTGGGTCT	0.717264409
2864	GTATTATATCAAGTTTAT	0.716615384
8595	GTTGCTACTGTATTATAT	0.716422523
8004	CTGGTAGGGCTATACATT	0.715925932
8113	CCTGAAGGGTACTAGTAG	0.715843766
2549	GCTGTACTATTATGGTTT	0.715770082
2206	GTACTCCAAGTACTATTA	0.714896645
8277	GTGTTTAGCATGGTGTTT	0.714750539
3283	GGTACCCCATAATAGACT	0.714629416
6874	CATTTAGTACTGTCTTTT	0.714101576

6206	GTATTACTTCTGTTAGTG	0.713673906
2984	TACTATTGGTATTAGTAT	0.713405215
5817	CACTAAGGGAGGGGTATT	0.712674249
3181	GTGGGTACACAGGCATGT	0.712590004
3183	GGGTACACAGGCATGTGT	0.712590004
2550	CTGTACTATTATGGTTTT	0.712513314
5588	GTGAGTCTGTTACTATGT	0.712486532
2835	CTTATAGCTGGTAGTATC	0.712455323
2503	GTATTGTTGTTGGGTCTT	0.712385491
7138	ACAGGTGTAGGTCCTACT	0.712249882
3737	GTACAAGCAGTTTTAGGC	0.711721197
6228	GGTTCCTCTAAGGAGTTT	0.711534368
4143	GCTAGTGCCAAGTATTGT	0.711521478
6190	GCTTCTTCTGTTAGTGGT	0.711178656

9.4 Top 100 antisense PNA sequences for ANGPTL3

Serial No.	PNA sequences	Predicted scores
1798	GTAGCGTATAGTTGGTTT	0.78056604
782	GTATACAGTATATATTTT	0.774777847
2560	GTAGTTTATATGTAGTTC	0.771331419
747	GTTTTCATAGTATTAAGT	0.758403393
2431	GAATTAAGTTAGTTAGTT	0.756133384
1115	GTACTAGTTTTTTAAACT	0.754725289
1084	GTATTTAGTCAAGTTTAG	0.743006111
1322	GGTATTCTTTAAGGTTAT	0.742737238
1075	CAGTCCTCTGTATTTAGT	0.736844119
1110	CAAGAGTACTAGTTTTTT	0.735817338
1792	CTAGATGTAGCGTATAGT	0.735302941
2558	TTGTAGTTTATATGTAGT	0.734988069
2559	TGTAGTTTATATGTAGTT	0.734988069
317	GTAGGTCACCATATAACT	0.731716764
2555	GACTTGTAGTTTATATGT	0.728730637
2557	CTTGTAGTTTATATGTAG	0.728323385
839	GTGACTTAGTAGTCATCT	0.723046852
1621	GTTTGTTATATTTACCAT	0.722172575
2561	TAGTTTATATGTAGTTCT	0.721562617
2382	GTTTTAAGTGAAGTTACT	0.721430754
2434	TTAAGTTAGTTAGTTGCT	0.721345765

1082	CTGTATTTAGTCAAGTTT	0.720777074
2438	GTTAGTTAGTTGCTCTTC	0.718059634
2394	GTTACTTCTGGGTGTTCT	0.715669918
2199	GGAGTAGTTCTTGGTGCT	0.715662445
1852	GTTTGTTGTCTTTCCAGT	0.713433431
879	CTTATTTTAATACAGTAT	0.711943048
2202	GTAGTTCTTGGTGCTCTT	0.711178656
1625	GTTATATTTACCATTTAG	0.710923011
170	CCTTTATTATTTTGTACT	0.710643929
305	GTATTGACAAAGGTAGGT	0.709129479
1080	CTCTGTATTTAGTCAAGT	0.708680686
599	GTAACTTGTTACGAGGTT	0.707884916
2563	GTTTATATGTAGTTCTTC	0.706493459
171	CTTTATTATTTTGTACTT	0.70635991
128	GACAGCTTAGTCTGTGGT	0.705598029
2407	GTTCTGGAGTTTCAGGTT	0.705032091
2308	GGTTTAATTGTTTATATT	0.704073558
1801	GCGTATAGTTGGTTTCGT	0.7040334
2437	AGTTAGTTAGTTGCTCTT	0.703890336
809	GGTATAGTGATACCTCAT	0.70246359
606	GTTACGAGGTTTATTTCT	0.701629
843	CTTAGTAGTCATCTCCAT	0.701468712
1091	GTCAAGTTTAGAGTTTTA	0.701067157
1635	CCATTTAGGTTGTTTTCT	0.701023275
2305	GTTGGTTTAATTGTTTAT	0.699925841
2571	GTAGTTCTTCTCAGTTCC	0.699616911
2164	GTTTTACATTTCTTATTT	0.698986323
1087	TTTAGTCAAGTTTAGAGT	0.698278473
1088	TTAGTCAAGTTTAGAGTT	0.698278473
1089	TAGTCAAGTTTAGAGTTT	0.698278473
1539	GTTGATTTTATAGAGTAT	0.698254756
316	GGTAGGTCACCATATAAC	0.696833915
2246	GGGTTCTTGAATACTAGT	0.695597671
1797	TGTAGCGTATAGTTGGTT	0.692419113
2562	AGTTTATATGTAGTTCTT	0.692324161
2389	GTGAAGTTACTTCTGGGT	0.691449953
2391	GAAGTTACTTCTGGGTGT	0.691449953
1627	TATATTTACCATTTAGGT	0.688933433
1620	GGTTTGTTATATTTACCA	0.687574399

781	TGTATACAGTATATATTT	0.686630919
2574	GTTCTTCTCAGTTCCTTT	0.686582528
1113	GAGTACTAGTTTTTTAAA	0.686155955
847	GTAGTCATCTCCATTTGT	0.683534806
159	GATTATTTTTACCTTTAT	0.683091737
1096	GTTTAGAGTTTTAACAAG	0.682754453
603	CTTGTTACGAGGTTTATT	0.68157585
1614	GCTCTTGGTTTGTTATAT	0.681487034
1642	GGTTGTTTCTCCACACT	0.681133719
2494	CTAGGAGGCTTTCAAGTT	0.680961136
1103	GTTTTAACAAGAGTACTA	0.680915879
2198	GGGAGTAGTTCTTGGTGC	0.680779596
752	CATAGTATTAAGTGTTAA	0.680525093
2436	AAGTTAGTTAGTTGCTCT	0.680010922
1631	TTTACCATTTAGGTTGTT	0.679308933
1632	TTACCATTTAGGTTGTTT	0.679308933
1633	TACCATTTAGGTTGTTTT	0.679308933
1793	TAGATGTAGCGTATAGTT	0.679305552
1074	CCAGTCCTCTGTATTTAG	0.678466065
1615	CTCTTGGTTTGTTATATT	0.678230266
1105	TTTAACAAGAGTACTAGT	0.678127195
1106	TTAACAAGAGTACTAGTT	0.678127195
1107	TAACAAGAGTACTAGTTT	0.678127195
455	CTTTAGGGATTTAGGGAT	0.67726767
1323	GTATTCTTTAAGGTTATG	0.676579889
806	TAAGGTATAGTGATACCT	0.675896099
841	GACTTAGTAGTCATCTCC	0.67419823
1065	GTACAATTACCAGTCCTC	0.673896084
1973	GTAGTTCTCCCACGTTTC	0.673747725
1109	ACAAGAGTACTAGTTTTT	0.672768153
532	GGGACTGGGTCTTTGTTT	0.672236476
1093	CAAGTTTAGAGTTTTAAC	0.672230734
1916	GTATATCTTCTCTAGGCC	0.671954532
1077	GTCCTCTGTATTTAGTCA	0.671539026
1551	GAGTATAACCTTCCATTT	0.671238008
749	TTTCATAGTATTAAGTGT	0.670256465
750	TTCATAGTATTAAGTGTT	0.670256465
1544	TTTTATAGAGTATAACCT	0.669742695
1545	TTTATAGAGTATAACCTT	0.669742695

2435	TAAGTTAGTTAGTTGCTC	0.669542188

9.5 Top 100 antisense PNA sequences for ANGPTL4

Serial No.	PNA sequences	Predicted scores
825	GGTCTAGGTGCTTGTGGT	0.734252788
110	GCCTGCAGTGGGTAGTAG	0.729652291
117	GTGGGTAGTAGCGGCCCC	0.727660972
111	CCTGCAGTGGGTAGTAGC	0.719128572
115	CAGTGGGTAGTAGCGGCC	0.710929168
109	GGCCTGCAGTGGGTAGTA	0.710451397
114	GCAGTGGGTAGTAGCGGC	0.709901917
378	GCAGGCTATAGGCCGTGT	0.701803994
381	GGCTATAGGCCGTGTCCT	0.698898624
196	GTACTGGCTGTTGAGGTT	0.698532985
62	GGGTAGGAGGCTGCCTCT	0.698044247
124	GTAGCGGCCCCGCCAGGT	0.696155525
816	CCACCTCATGGTCTAGGT	0.69279517
119	GGGTAGTAGCGGCCCCGC	0.684415394
120	GGTAGTAGCGGCCCCGCC	0.683875072
107	GTGGCCTGCAGTGGGTAG	0.683064123
112	CTGCAGTGGGTAGTAGCG	0.680194989
556	GGCTTCCCAGGGCCGGTT	0.678598741
826	GTCTAGGTGCTTGTGGTC	0.67762487
822	CATGGTCTAGGTGCTTGT	0.67399989
59	GTTGGGTAGGAGGCTGCC	0.671762509
1005	CAGGGGCTAACGGGAGGT	0.670105293
550	CTTGTAGGCTTCCCAGGG	0.669336858
549	CCTTGTAGGCTTCCCAGG	0.668796537
376	CTGCAGGCTATAGGCCGT	0.668325392
960	GTTGTGTCTGCAGGCTGT	0.6682846
548	GCCTTGTAGGCTTCCCAG	0.667228964
819	CCTCATGGTCTAGGTGCT	0.664858457
1297	GTACAAAGTTGGCATTAT	0.664570288
804	GGGCAGGCTTGGCCACCT	0.663972944
106	GGTGGCCTGCAGTGGGTA	0.663863229
553	GTAGGCTTCCCAGGGCCG	0.6638539
882	GGTGCTGCTTCTCCAGGT	0.662638444
1219	GCTCAGTAGCACGGCGGT	0.661682986

820	CTCATGGTCTAGGTGCTT	0.660574438
1017	GGAGGTCGGTGGACCCCT	0.659627849
116	AGTGGGTAGTAGCGGCCC	0.657404078
121	GTAGTAGCGGCCCCGCCA	0.65738545
108	TGGCCTGCAGTGGGTAGT	0.656250204
958	GAGTTGTGTCTGCAGGCT	0.654518598
325	GCTGGGTGGGACGGTGGT	0.653394093
985	CTCAGGGTCCACCCGGCT	0.653181169
987	CAGGGTCCACCCGGCTCT	0.653181169
981	GGACCTCAGGGTCCACCC	0.650161265
1009	GGCTAACGGGAGGTCGGT	0.649653651
259	GGCGCAGTTCTTGTCCCT	0.649074378
561	CCCAGGGCCGGTTGAAGT	0.647626732
379	CAGGCTATAGGCCGTGTC	0.646743649
951	GAGCCTTGAGTTGTGTCT	0.646448336
840	GGTCCAGGAGGCCAAACT	0.645971053
49	GTACAAGAAAGTTGGGTA	0.645827548
1021	GTCGGTGGACCCCTCGGT	0.643449379
286	GTGATCCTGGTCCCAAGT	0.643244665
238	CCAGCCTCCAGAGAGGCT	0.642782758
310	GGGTACGGAGAGGCCGCT	0.642430359
382	GCTATAGGCCGTGTCCTC	0.642270706
828	CTAGGTGCTTGTGGTCCA	0.638861392
914	GCCACCTTGTGGAAGAGT	0.637204528
103	GGTGGTGGCCTGCAGTGG	0.636475954
104	GTGGTGGCCTGCAGTGGG	0.636475954
55	GAAAGTTGGGTAGGAGGC	0.636175493
355	GCCGGCCACGGGTGCAGT	0.635759242
241	GCCTCCAGAGAGGCTCTT	0.634566118
472	GCTGTTGCGGTCCCCCGT	0.63405835
915	CCACCTTGTGGAAGAGTT	0.633947761
814	GGCCACCTCATGGTCTAG	0.633389865
815	GCCACCTCATGGTCTAGG	0.633389865
195	AGTACTGGCTGTTGAGGT	0.632560109
369	CAGTGAGCTGCAGGCTAT	0.632377102
63	GGTAGGAGGCTGCCTCTG	0.631886897
906	GCTGCTGGGCCACCTTGT	0.631667556
190	GCGGAAGTACTGGCTGTT	0.631407992
127	GCGGCCCCGCCAGGTCTT	0.631317565

832	GTGCTTGTGGTCCAGGAG	0.631186718
371	GTGAGCTGCAGGCTATAG	0.630620368
227	GTGCCAAACCACCAGCCT	0.630466549
566	GGCCGGTTGAAGTCCACT	0.629345675
113	TGCAGTGGGTAGTAGCGG	0.62902194
151	GATTCCCTTCTTAAGCTT	0.628683081
835	CTTGTGGTCCAGGAGGCC	0.628442295
834	GCTTGTGGTCCAGGAGGC	0.627415044
613	CCAGCCTCCATCTGAGGT	0.627409415
817	CACCTCATGGTCTAGGTG	0.627178142
298	CCAAGTGGAGAAGGGTAC	0.627138327
373	GAGCTGCAGGCTATAGGC	0.626848694
810	GCTTGGCCACCTCATGGT	0.626513983
949	CTGAGCCTTGAGTTGTGT	0.626395186
129	GGCCCCGCCAGGTCTTCC	0.625714521
507	CCAGACCCAGCCAGAACT	0.624886255
1016	GGGAGGTCGGTGGACCCC	0.624745
360	CCACGGGTGCAGTGAGCT	0.623980922
544	CCCCGCCTTGTAGGCTTC	0.623979748
545	CCCGCCTTGTAGGCTTCC	0.623979748
546	CCGCCTTGTAGGCTTCCC	0.623979748
187	GGAGCGGAAGTACTGGCT	0.62246633
56	AAAGTTGGGTAGGAGGCT	0.622346745
60	TTGGGTAGGAGGCTGCCT	0.621993707
480	GGTCCCCCGTGATGCTAT	0.621749917
441	CATCCCAGTCCCGCAGCT	0.621375289
131	CCCCGCCAGGTCTTCCAG	0.619993365

9.6 Top 100 antisense PNA sequences for APOB

Serial No.	PNA sequences	Predicted scores
7930	GTCTAGTAGAGTTAGGTC	0.792081379
7925	GGGGAGTCTAGTAGAGTT	0.788355126
8499	GGTAGGCTCCTTTTAGGT	0.787291188
8500	GTAGGCTCCTTTTAGGTT	0.782466847
5079	GGTTTACTCTTAGGTGTT	0.779178815
7929	AGTCTAGTAGAGTTAGGT	0.777912081
8538	GGGGTTCTAGCCGTAGTT	0.775596507
5080	GTTTACTCTTAGGTGTTT	0.774354474

7932	CTAGTAGAGTTAGGTCAG	0.773059117
2632	GGGGAGTGTTGGTAGGTT	0.772453688
12613	GGTCCCTGTAGGGTTTGT	0.771713596
8539	GGGTTCTAGCCGTAGTTT	0.770772167
7927	GGAGTCTAGTAGAGTTAG	0.768785945
7928	GAGTCTAGTAGAGTTAGG	0.768785945
12742	CAGGGCCACCAGGTAGGT	0.767665355
1355	CTAGTGTATATCCCAGGT	0.763525648
7940	GTTAGGTCAGCCAGAGTT	0.763093667
1717	GTGTTCCCAGTGGTACTT	0.756824839
1719	GTTCCCAGTGGTACTTGT	0.756824839
11956	GGCAGTGAGGGTAGTTTT	0.756568945
11985	CTTTAGGAAGGTAGTTAT	0.756182877
7224	GGTACTTAGTATCCACAT	0.755596179
3108	GGTTCTTAGTGTTAGCAT	0.752737596
8599	GTTTAAAGTAGTTACCAG	0.752216982
7225	GTACTTAGTATCCACATT	0.750771839
7926	GGGAGTCTAGTAGAGTTA	0.749585051
3109	GTTCTTAGTGTTAGCATT	0.747913256
12609	CCTGGGTCCCTGTAGGGT	0.747343353
8593	GTCACTGTTTAAAGTAGT	0.74708614
13537	GTAAGTAGGTTCATCTTT	0.745654565
1564	CTCTTGGTAGGTCCCAGT	0.745300038
1915	GTAGTAGAAGTTCCATTT	0.744773692
6612	GTGGTAGTTCCAGAGCCT	0.743391914
12610	CTGGGTCCCTGTAGGGTT	0.743059334
1908	GAGGGCTGTAGTAGAAGT	0.742854989
3742	GGTTAAGCTCACAGTACT	0.738998921
6435	GTGTATCTTCTAGGGTCT	0.73827616
11393	GCTACTTCCAGTTTTACT	0.737023178
5320	GTGGAAGTATTTAGTGTT	0.734753694
13824	CATAGTTGTATGTGTACT	0.734332873
12616	CCCTGTAGGGTTTGTCTT	0.733410653
7256	GCAGTACTACTTCCACTT	0.731783102
2153	CCTTTAGTCTTAGAGGCT	0.731726967
3273	CTAGCTGTAAGTGGTTTT	0.730754987
12618	CTGTAGGGTTTGTCTTAT	0.730319397
3267	GGCCCTCTAGCTGTAAGT	0.729154341
7935	GTAGAGTTAGGTCAGCCA	0.729147932

7257	CAGTACTACTTCCACTTT	0.728526334
2631	CGGGGAGTGTTGGTAGGT	0.727280406
6603	CAGCTTTTTGTGGTAGTT	0.727207664
1569	GGTAGGTCCCAGTGGTGC	0.727099408
1570	GTAGGTCCCAGTGGTGCC	0.726559086
6437	GTATCTTCTAGGGTCTCT	0.724850709
12985	GGTTAGTTTTTTCAGTTC	0.724350008
9295	GTAGTTCTCATACTTTAG	0.724234226
12986	GTTAGTTTTTTCAGTTCC	0.723809686
6304	GTCAGTAAGGTTCTTAGC	0.722990332
3270	CCTCTAGCTGTAAGTGGT	0.722942618
4449	GTTTGTTTTTCTTATACT	0.722265343
9883	GGACCAGTTGTACAAGTT	0.721751714
9415	GTAGGAGGAGTTAAACCT	0.721597451
7711	GTTTTCCAGTACAACTAT	0.721415642
11389	GTTGGCTACTTCCAGTTT	0.720989922
3567	CTCCTTTAGCGGTAGAGT	0.720032286
8507	CCTTTTAGGTTACCAGCC	0.719881064
1347	GTTCCTCCCTAGTGTATA	0.719630189
12372	GGTCCTTGTCTTTAGGCT	0.718957951
169	GCTTATAGTCTACTGCCT	0.718847266
3271	CTCTAGCTGTAAGTGGTT	0.718658599
1715	GTGTGTTCCCAGTGGTAC	0.718446713
8597	CTGTTTAAAGTAGTTACC	0.717707492
8503	GGCTCCTTTTAGGTTACC	0.716488765
1236	GTGTAATCACTAGGTCTT	0.716428717
11962	GAGGGTAGTTTTCAGCAT	0.716369198
2150	GTTCCTTTAGTCTTAGAG	0.715844602
11400	CCAGTTTTACTCCAGCCT	0.715240789
12614	GTCCCTGTAGGGTTTGTC	0.715085678
3569	CCTTTAGCGGTAGAGTAC	0.714608145
8544	CTAGCCGTAGTTTCCCAT	0.714262966
8540	GGTTCTAGCCGTAGTTTC	0.714144249
12612	GGGTCCCTGTAGGGTTTG	0.713875863
8541	GTTCTAGCCGTAGTTTCC	0.713603927
13073	CCCATCTTCTTAGTACCT	0.713528181
8584	GTATTTCAGGTCACTGTT	0.712881394
1913	CTGTAGTAGAAGTTCCAT	0.712624154
6524	GTTATTTTGGTGTCCTGT	0.711845169

5074	GTTTTGGTTTACTCTTAG	0.711692401
11401	CAGTTTTACTCCAGCCTT	0.71095677
8505	CTCCTTTTAGGTTACCAG	0.710767608
6372	GGCCTACCAGAGACAGGT	0.710751652
1558	CTTCCACTCTTGGTAGGT	0.710461107
10965	CTCCTGTAGGCCTCAGTT	0.710253863
1104	GTAGCACCTCTGTGGTCT	0.710222598
9653	GGGTTGTTTCCAAGTTTT	0.709970021
7933	TAGTAGAGTTAGGTCAGC	0.709794777
8537	AGGGGTTCTAGCCGTAGT	0.709623632
1710	GCCCTGTGTGTTCCCAGT	0.709489105
8038	GGTCTTGAGTTTCCAGGT	0.70931611
13074	CCATCTTCTTAGTACCTT	0.709244162
10849	GGTAGCCTCAGTCTGCTT	0.709226612

9.7 Top 100 antisense PNA sequences for APOC3

Serial No.	PNA sequences	Predicted scores
260	GGTGCTCCAGTAGTCTTT	0.739448446
65	GTGGGGTAGGAGAGCACT	0.718401435
269	GTAGTCTTTCAGGGAACT	0.702010273
259	CGGTGCTCCAGTAGTCTT	0.694275163
161	GGCAGGTGGACTTGGGGT	0.689074546
163	CAGGTGGACTTGGGGTAT	0.686470219
264	CTCCAGTAGTCTTTCAGG	0.685418989
263	GCTCCAGTAGTCTTTCAG	0.683851417
64	GGTGGGGTAGGAGAGCAC	0.683518586
261	GTGCTCCAGTAGTCTTTC	0.682820529
169	GACTTGGGGTATTGAGGT	0.677372707
62	GAGGTGGGGTAGGAGAGC	0.676321724
92	GTCCCTTTTAAGCAACCT	0.675645726
171	CTTGGGGTATTGAGGTCT	0.674789567
156	GGATAGGCAGGTGGACTT	0.671136644
49	GGGGGCCAGGCATGAGGT	0.670412623
466	CAAGGAGTACCCGGGGCT	0.66902465
266	CCAGTAGTCTTTCAGGGA	0.665877545
54	CCAGGCATGAGGTGGGGT	0.663660578
57	GGCATGAGGTGGGGTAGG	0.662444166
174	GGGGTATTGAGGTCTCAG	0.65749548

175	GGGTATTGAGGTCTCAGG	0.65749548
253	CCTTAACGGTGCTCCAGT	0.656221243
9	CTTCTTGTCCAGCTTTAT	0.653880713
108	CTACAGGGGCAGCCCTGG	0.650795676
162	GCAGGTGGACTTGGGGTA	0.65030447
107	CCTACAGGGGCAGCCCTG	0.650255354
256	TAACGGTGCTCCAGTAGT	0.648681407
106	ACCTACAGGGGCAGCCCT	0.648539178
319	GGGCCACCTGGGACTCCT	0.648297558
5	GCAGCTTCTTGTCCAGCT	0.6467537
150	CAGGATGGATAGGCAGGT	0.646262998
296	GGTCACCCAGCCCCTGGC	0.645594577
176	GGTATTGAGGTCTCAGGC	0.645404189
470	GAGTACCCGGGGCTGCAT	0.645400765
297	GTCACCCAGCCCCTGGCC	0.645054255
6	CAGCTTCTTGTCCAGCTT	0.643496933
59	CATGAGGTGGGGTAGGAG	0.641926119
301	CCCAGCCCCTGGCCTGCT	0.639726536
362	GGTCTTGGTGGCGTGCTT	0.6395947
41	GCCTGGAGGGGGGCCAGG	0.638808864
68	GGGTAGGAGAGCACTGAG	0.638478083
104	CAACCTACAGGGGCAGCC	0.638477664
267	CAGTAGTCTTTCAGGGAA	0.637714111
103	GCAACCTACAGGGGCAGC	0.637450413
94	CCCTTTTAAGCAACCTAC	0.636742983
506	GCAGCTGCCTCTAGGGAT	0.636223776
210	GGTCTGACCTCAGGGTCC	0.63606598
265	TCCAGTAGTCTTTCAGGG	0.63424594
15	GTCCAGCTTTATTGGGAG	0.634005372
165	GGTGGACTTGGGGTATTG	0.633300976
258	ACGGTGCTCCAGTAGTCT	0.631225978
96	CTTTTAAGCAACCTACAG	0.630534897
83	GAGAATACTGTCCCTTTT	0.629338517
164	AGGTGGACTTGGGGTATT	0.62866111
42	CCTGGAGGGGGGCCAGGC	0.628285146
248	CTTGTCCTTAACGGTGCT	0.627951453
182	GAGGTCTCAGGCAGCCAC	0.627910947
113	GGGGCAGCCCTGGAGATT	0.627296187
316	GCTGGGCCACCTGGGACT	0.627217156

55	CAGGCATGAGGTGGGGTA	0.625430825
245	GAACTTGTCCTTAACGGT	0.62345156
207	GTTGGTCTGACCTCAGGG	0.622252529
58	GCATGAGGTGGGGTAGGA	0.621157653
503	GGAGCAGCTGCCTCTAGG	0.620826494
504	GAGCAGCTGCCTCTAGGG	0.620826494
20	GCTTTATTGGGAGGCCAG	0.620704265
168	GGACTTGGGGTATTGAGG	0.619534974
67	GGGGTAGGAGAGCACTGA	0.619277189
177	GTATTGAGGTCTCAGGCA	0.618914566
254	CTTAACGGTGCTCCAGTA	0.61799149
8	GCTTCTTGTCCAGCTTTA	0.617714964
45	GGAGGGGGGCCAGGCATG	0.61257489
48	GGGGGGCCAGGCATGAGG	0.61257489
469	GGAGTACCCGGGGCTGCA	0.610802589
95	CCTTTTAAGCAACCTACA	0.610793682
184	GGTCTCAGGCAGCCACGG	0.610522662
17	CCAGCTTTATTGGGAGGC	0.610180547
18	CAGCTTTATTGGGAGGCC	0.610180547
21	CTTTATTGGGAGGCCAGC	0.610180547
211	GTCTGACCTCAGGGTCCA	0.609576357
12	CTTGTCCAGCTTTATTGG	0.609468431
1	CATAGCAGCTTCTTGTCC	0.609266524
195	GCCACGGCTGAAGTTGGT	0.608265273
295	CGGTCACCCAGCCCCTGG	0.607688245
347	CAGTGCATCCTTGGCGGT	0.607611593
485	CATGGCACCTCTGTTCCT	0.606336487
373	CGTGCTTCATGTAACCCT	0.605461176
157	GATAGGCAGGTGGACTTG	0.604979294
50	GGGGCCAGGCATGAGGTG	0.604255273
51	GGGCCAGGCATGAGGTGG	0.604255273
52	GGCCAGGCATGAGGTGGG	0.604255273
53	GCCAGGCATGAGGTGGGG	0.604255273
349	GTGCATCCTTGGCGGTCT	0.603678972
463	CAACAAGGAGTACCCGGG	0.60264058
406	GGGAGGCATCCTCGGCCT	0.602516605
43	CTGGAGGGGGGCCAGGCA	0.602335845
299	CACCCAGCCCCTGGCCTG	0.602046221
191	GGCAGCCACGGCTGAAGT	0.602029211

472	GTACCCGGGGCTGCATGG	0.601329034

9.8 Top 100 antisense PNA sequences for LPA

Serial No.	PNA sequences	Predicted scores
3202	GTAGTAGCAGTCCTGTAC	0.781941537
3201	GGTAGTAGCAGTCCTGTA	0.770340672
2429	GGGTAGTATTCTGTGGTT	0.76862234
1154	GTCCCAGTAACAGTGGTT	0.756931196
1405	GTAGTTTTCTGGGGTCCT	0.754004125
1747	GTAGTTTTCTGGGGTCCT	0.754004125
2431	GTAGTATTCTGTGGTTCT	0.746877271
6169	GTAGTTTTCTGTGGTCCT	0.745684509
1980	GTGTCAGGTTGCAGTACT	0.743349803
2640	GTGTCAGGTTGCAGTACT	0.743349803
2982	GTGTCAGGTTGCAGTACT	0.743349803
3324	GTGTCAGGTTGCAGTACT	0.743349803
3666	GTGTCAGGTTGCAGTACT	0.743349803
3205	GTAGCAGTCCTGTACCCC	0.742052021
3211	GTCCTGTACCCCGGGGGT	0.741527317
1676	GTTGTGTAACACCAGGGT	0.740342057
1679	GTGTAACACCAGGGTTGT	0.740342057
3216	GTACCCCGGGGGTTTCCT	0.740198254
1681	GTAACACCAGGGTTGTTT	0.739012993
3224	GGGGTTTCCTCAGTCAGT	0.738466773
3195	CATAATGGTAGTAGCAGT	0.738267585
3773	GGGTAGTATTCTGGGGTC	0.725138379
4115	GGGTAGTATTCTGGGGTC	0.725138379
4457	GGGTAGTATTCTGGGGTC	0.725138379
4799	GGGTAGTATTCTGGGGTC	0.725138379
5141	GGGTAGTATTCTGGGGTC	0.725138379
5483	GGGTAGTATTCTGGGGTC	0.725138379
5825	GGGTAGTATTCTGGGGTC	0.725138379
2418	GGCCACCATTTGGGTAGT	0.725069054
3774	GGTAGTATTCTGGGGTCC	0.724598058
4116	GGTAGTATTCTGGGGTCC	0.724598058
4458	GGTAGTATTCTGGGGTCC	0.724598058
4800	GGTAGTATTCTGGGGTCC	0.724598058
5142	GGTAGTATTCTGGGGTCC	0.724598058

5484	GGTAGTATTCTGGGGTCC	0.724598058
5826	GGTAGTATTCTGGGGTCC	0.724598058
2056	CCTGCAGTAGTTCCTGGT	0.723679314
2398	CCTGCAGTAGTTCCTGGT	0.723679314
2716	CCTGCAGTAGTTCCTGGT	0.723679314
3058	CCTGCAGTAGTTCCTGGT	0.723679314
532	GGTTTCTCCCCAGCCAGT	0.723544702
2420	CCACCATTTGGGTAGTAT	0.722464727
1734	GGCCAGCATTTGGGTAGT	0.721118113
3078	GGCCAGCATTTGGGTAGT	0.721118113
3213	CCTGTACCCCGGGGGTTT	0.720145103
1403	GGGTAGTTTTCTGGGGTC	0.719661598
1745	GGGTAGTTTTCTGGGGTC	0.719661598
3089	GGGTAGTTTTCTGGGGTC	0.719661598
1303	GTTGCAGTACTCCCACCT	0.719493543
1987	GTTGCAGTACTCCCACCT	0.719493543
2989	GTTGCAGTACTCCCACCT	0.719493543
3331	GTTGCAGTACTCCCACCT	0.719493543
3673	GTTGCAGTACTCCCACCT	0.719493543
4015	GTTGCAGTACTCCCACCT	0.719493543
4357	GTTGCAGTACTCCCACCT	0.719493543
4699	GTTGCAGTACTCCCACCT	0.719493543
5041	GTTGCAGTACTCCCACCT	0.719493543
5383	GTTGCAGTACTCCCACCT	0.719493543
5725	GTTGCAGTACTCCCACCT	0.719493543
6067	GTTGCAGTACTCCCACCT	0.719493543
1404	GGTAGTTTTCTGGGGTCC	0.719121276
1746	GGTAGTTTTCTGGGGTCC	0.719121276
3090	GGTAGTTTTCTGGGGTCC	0.719121276
3422	CCAGCATTTGGGTAGTAT	0.718513786
3764	CCAGCATTTGGGTAGTAT	0.718513786
4106	CCAGCATTTGGGTAGTAT	0.718513786
4448	CCAGCATTTGGGTAGTAT	0.718513786
4790	CCAGCATTTGGGTAGTAT	0.718513786
5132	CCAGCATTTGGGTAGTAT	0.718513786
5474	CCAGCATTTGGGTAGTAT	0.718513786
5816	CCAGCATTTGGGTAGTAT	0.718513786
2421	CACCATTTGGGTAGTATT	0.718180708
1735	GCCAGCATTTGGGTAGTT	0.716293772

3079	GCCAGCATTTGGGTAGTT	0.716293772
6157	GCCAGCATTTGGGTAGTT	0.716293772
3200	TGGTAGTAGCAGTCCTGT	0.716139479
3765	CAGCATTTGGGTAGTATT	0.714229767
4107	CAGCATTTGGGTAGTATT	0.714229767
4449	CAGCATTTGGGTAGTATT	0.714229767
4791	CAGCATTTGGGTAGTATT	0.714229767
5133	CAGCATTTGGGTAGTATT	0.714229767
5475	CAGCATTTGGGTAGTATT	0.714229767
5817	CAGCATTTGGGTAGTATT	0.714229767
1736	CCAGCATTTGGGTAGTTT	0.713037004
3080	CCAGCATTTGGGTAGTTT	0.713037004
6158	CCAGCATTTGGGTAGTTT	0.713037004
2430	GGTAGTATTCTGTGGTTC	0.711994422
1153	CGTCCCAGTAACAGTGGT	0.711757913
6167	GGGTAGTTTTCTGTGGTC	0.711341981
2423	CCATTTGGGTAGTATTCT	0.711324397
6168	GGTAGTTTTCTGTGGTCC	0.710801659
1538	GGGGTTTGCTCAGTTGGT	0.710321327
3767	GCATTTGGGTAGTATTCT	0.710297146
4109	GCATTTGGGTAGTATTCT	0.710297146
4451	GCATTTGGGTAGTATTCT	0.710297146
4793	GCATTTGGGTAGTATTCT	0.710297146
5135	GCATTTGGGTAGTATTCT	0.710297146
5477	GCATTTGGGTAGTATTCT	0.710297146
5819	GCATTTGGGTAGTATTCT	0.710297146
2545	GTAGCAGTCCTGGACTGT	0.709882047

9.9 Top 100 antisense PNA sequences for PCSK9

Serial No.	PNA sequences	Predicted scores
761	GGTTGGGGGTCAGTACCC	0.735085947
753	GGCCACCAGGTTGGGGGT	0.734065293
489	GGGTAGCAGGCAGCACCT	0.731591929
767	GGGTCAGTACCCGCTGGT	0.723227941
759	CAGGTTGGGGGTCAGTAC	0.718354143
179	CAGTCAGGGTCCAGCCCT	0.715921257
368	GGTCCTCCACCTCCCAGT	0.715399255
1845	GTAGGTGCCAGGCAACCT	0.713408256

1581	GTACCGTGGAGGGGTAAT	0.709932077
758	CCAGGTTGGGGGTCAGTA	0.706212956
1833	CAGCACCACCACGTAGGT	0.705702665
156	GGTCCCAGGGAGGGCACT	0.703378563
1023	CCCCAAAGTCCCCAGGGT	0.69801787
410	GGCCCTGTTGGTGGCAGT	0.697268642
488	GGGGTAGCAGGCAGCACC	0.69670908
1380	CACCCCTGCCAGGTGGGT	0.696010772
762	GTTGGGGGTCAGTACCCG	0.695612042
1100	GGGAGTAGAGGCAGGCAT	0.695455494
676	GTAGGCCCCGAGTGTGCT	0.693870034
1216	GGCAGCAGCACCACCAGT	0.693560454
1578	CCGGTACCGTGGAGGGGT	0.692269787
1952	GCACCAGCTCCTCGTAGT	0.69042247
477	GCAGTTGGCCTGGGGTAG	0.686559399
480	GTTGGCCTGGGGTAGCAG	0.686559399
1229	CCAGTGGCCCCACAGGCT	0.684950369
75	GCTGGTGCTGCCTGTAGT	0.681869909
78	GGTGCTGCCTGTAGTGCT	0.681869909
1302	GCCCTTCCCTTGGCAGTT	0.681433484
1815	GAGGTGGGTCTCCTCCTT	0.681013246
178	CCAGTCAGGGTCCAGCCC	0.680498086
484	GCCTGGGGTAGCAGGCAG	0.680323337
134	CGTAGGCCCCCAGGACGT	0.680128741
177	GCCAGTCAGGGTCCAGCC	0.679470835
1200	GTACCCACCCGCCAGGGG	0.678945172
673	CGTGTAGGCCCCGAGTGT	0.678918586
516	GTAGACACCCTCACCCCC	0.67853933
754	GCCACCAGGTTGGGGGTC	0.677437376
757	ACCAGGTTGGGGGTCAGT	0.677109505
181	GTCAGGGTCCAGCCCTCC	0.676565465
746	GCAGGGCGGCCACCAGGT	0.676264035
478	CAGTTGGCCTGGGGTAGC	0.676035681
1580	GGTACCGTGGAGGGGTAA	0.675333901
175	CAGCCAGTCAGGGTCCAG	0.674290001
152	GGGAGGTCCCAGGGAGGG	0.673390143
747	CAGGGCGGCCACCAGGTT	0.673007267
1272	CTCCAGGCCTATGAGGGT	0.672297202
756	CACCAGGTTGGGGGTCAG	0.672256541

485	CCTGGGGTAGCAGGCAGC	0.669799618
1592	GGGTAATCCGCTCCAGGT	0.669213196
353	GCTTGTGGGTGCCAAGGT	0.669105209
1943	GCAAGGCTAGCACCAGCT	0.667842
126	GTCTACGGCGTAGGCCCC	0.667161588
1034	CCAGGGTCACCGGCTGGT	0.667146942
768	GGTCAGTACCCGCTGGTC	0.666600023
769	GTCAGTACCCGCTGGTCC	0.666059702
490	GGTAGCAGGCAGCACCTG	0.66543458
491	GTAGCAGGCAGCACCTGG	0.66543458
765	GGGGGTCAGTACCCGCTG	0.665390208
766	GGGGTCAGTACCCGCTGG	0.665390208
150	GTGGGAGGTCCCAGGGAG	0.665070526
760	AGGTTGGGGGTCAGTACC	0.664829053
1593	GGTAATCCGCTCCAGGTT	0.664388855
475	CTGCAGTTGGCCTGGGGT	0.664330361
135	GTAGGCCCCCAGGACGTG	0.663969014
141	CCCCAGGACGTGGGAGGT	0.663560471
1884	GTGGAAGGTGGCTGTGGT	0.663444654
1037	GGGTCACCGGCTGGTCCT	0.663214321
165	GAGGGCACTGCAGCCAGT	0.662918142
674	GTGTAGGCCCCGAGTGTG	0.662758859
420	GTGGCAGTGGACACGGGT	0.662491108
1518	GGTGTCTAGGAGATACAC	0.662092881
1519	GTGTCTAGGAGATACACC	0.661552559
153	GGAGGTCCCAGGGAGGGC	0.661298852
483	GGCCTGGGGTAGCAGGCA	0.661122443
375	CACCTCCCAGTGGGAGCT	0.661040023
1099	GGGGAGTAGAGGCAGGCA	0.660857318
132	GGCGTAGGCCCCCAGGAC	0.66019734
1500	GTGGTCACTCTGTATGCT	0.660002852
1503	GTCACTCTGTATGCTGGT	0.660002852
121	GTGTTGTCTACGGCGTAG	0.659410269
1097	CTGGGGAGTAGAGGCAGG	0.657640012
1949	CTAGCACCAGCTCCTCGT	0.656943868
1096	GCTGGGGAGTAGAGGCAG	0.65607244
123	GTTGTCTACGGCGTAGGC	0.655638595
687	GTGTGCTGACCACACAGT	0.655261433
1839	CACCACGTAGGTGCCAGG	0.654434072

1579	CGGTACCGTGGAGGGGTA	0.654040033
1838	CCACCACGTAGGTGCCAG	0.653893751
734	GGGTGCTGGGGGGCAGGG	0.65365551
174	GCAGCCAGTCAGGGTCCA	0.653521534
1093	GAGGCTGGGGAGTAGAGG	0.65336684
1497	CCGGTGGTCACTCTGTAT	0.652830699
755	CCACCAGGTTGGGGGTCA	0.652515325
1020	GGTCCCCAAAGTCCCCAG	0.651913671
1021	GTCCCCAAAGTCCCCAGG	0.651913671
1030	GTCCCCAGGGTCACCGGC	0.6512366
369	GTCCTCCACCTCCCAGTG	0.649241906
1897	GTGGTTCCGTGCTCGGGT	0.648980421
560	GCTTGCCCCCTTGGGCCT	0.648430624
1521	GTCTAGGAGATACACCTC	0.648127108

9.10 Top 100 antisense PNA sequences for GCGR

Serial No.	PNA sequences	Predicted scores
194	GGGGGGTAGGTGGGGGCT	0.77746143
188	GGGGGTGGGGGGTAGGTG	0.746350255
189	GGGGTGGGGGGTAGGTGG	0.746350255
190	GGGTGGGGGGTAGGTGGG	0.746350255
191	GGTGGGGGGTAGGTGGGG	0.746350255
192	GTGGGGGGTAGGTGGGGG	0.746350255
1023	CCAGCAGCCAGCAGTAGT	0.743794072
1024	CAGCAGCCAGCAGTAGTT	0.739510054
1306	GTAGCCCACTGTGTACAT	0.734111757
197	GGGTAGGTGGGGGCTGCT	0.734095639
199	GTAGGTGGGGGCTGCTGT	0.725776022
187	TGGGGGTGGGGGGTAGGT	0.719536337
985	GGCCAGGCCCAGCAGGTT	0.717973306
301	CTGGGGTCCCAGCAGGGT	0.714220875
186	CTGGGGGTGGGGGGTAGG	0.712871653
195	GGGGGTAGGTGGGGGCTG	0.711304081
987	CCAGGCCCAGCAGGTTGT	0.711221262
1302	GGCTGTAGCCCACTGTGT	0.709806622
1811	CAGTCCTGGTGGGTGTGT	0.709182366
1496	GGCAGGTACCAGGGGCAG	0.708800371
1497	GCAGGTACCAGGGGCAGG	0.708800371

724	GTAGTCTGTGTGGTGCAT	0.703983216
993	CCAGCAGGTTGTGCAGGT	0.703982288
285	CAGAGTCCAGCCCTAGCT	0.703136928
1599	GGGGCAGCAGGCTCAGGT	0.702673543
1605	GCAGGCTCAGGTTGTGGT	0.700589988
1300	CAGGCTGTAGCCCACTGT	0.700313792
1813	GTCCTGGTGGGTGTGTAC	0.699825603
1501	GTACCAGGGGCAGGAGAT	0.699406435
196	GGGGTAGGTGGGGGCTGC	0.69921279
349	GCCACCAGCCAAGGGGGT	0.698585154
1600	GGGCAGCAGGCTCAGGTT	0.697849203
999	GGTTGTGCAGGTACAGGC	0.695165847
1000	GTTGTGCAGGTACAGGCC	0.694625526
303	GGGGTCCCAGCAGGGTTC	0.694566836
1819	GTGGGTGTGTACGCTCCT	0.691462874
1026	GCAGCCAGCAGTAGTTGG	0.691165151
1020	CCACCAGCAGCCAGCAGT	0.690096442
1009	GTACAGGCCCTCCACCAG	0.689925601
1602	GCAGCAGGCTCAGGTTGT	0.689529586
281	GTGCCAGAGTCCAGCCCT	0.689487041
243	GGGCGTCCAGTTCTGGGT	0.689485214
1298	GACAGGCTGTAGCCCACT	0.68947148
997	CAGGTTGTGCAGGTACAG	0.689444691
1288	GGCCCCCAGGGACAGGCT	0.688996814
717	GGAACTTGTAGTCTGTGT	0.687954676
305	GGTCCCAGCAGGGTTCAG	0.687278107
1594	GGGAGGGGGCAGCAGGCT	0.686666837
1003	GTGCAGGTACAGGCCCTC	0.685484093
1473	GGTGTTGCACTTTGTGGT	0.68487225
244	GGCGTCCAGTTCTGGGTT	0.684660874
1030	CCAGCAGTAGTTGGCCAC	0.684592374
1304	CTGTAGCCCACTGTGTAC	0.684204985
1290	CCCCCAGGGACAGGCTGT	0.682244769
1493	CAAGGCAGGTACCAGGGG	0.681664189
1492	CCAAGGCAGGTACCAGGG	0.681123867
1027	CAGCCAGCAGTAGTTGGC	0.680641433
1006	CAGGTACAGGCCCTCCAC	0.679942204
1491	GCCAAGGCAGGTACCAGG	0.679556295
351	CACCAGCCAAGGGGGTCT	0.67894798

184	CACTGGGGGTGGGGGTA	0.678782002
1488	GGTGCCAAGGCAGGTACC	0.678525406
283	GCCAGAGTCCAGCCCTAG	0.678237476
246	CGTCCAGTTCTGGGTTGT	0.677908829
295	CCCTAGCTGGGGTCCCAG	0.677904896
1614	GGTTGTGGTGACACTGGT	0.676926574
289	GTCCAGCCCTAGCTGGGG	0.676877645
561	CAGCCACCAGCAGGCCCT	0.673889966
1875	CGTCCTGGCCAGTGGGGT	0.673716082
448	GGCCCTGTGGTTGCTGGT	0.67328287
1816	CTGGTGGGTGTGTACGCT	0.671950045
176	GACAGCCACACTGGGGGT	0.671900667
1029	GCCAGCAGTAGTTGGCCA	0.671423936
247	GTCCAGTTCTGGGTTGTC	0.671278535
1303	GCTGTAGCCCACTGTGTA	0.671036547
1008	GGTACAGGCCCTCCACCA	0.670724707
943	GATGCCCAGGTAGAGGCT	0.670389079
1498	CAGGTACCAGGGGCAGGA	0.66908143
996	GCAGGTTGTGCAGGTACA	0.668676224
553	GTAGAGGACAGCCACCAG	0.668580074
304	GGGTCCCAGCAGGGTTCA	0.668077213
313	CAGGGTTCAGAAGGGGCT	0.668007421
198	GGTAGGTGGGGGCTGCTG	0.667938289
284	CCAGAGTCCAGCCCTAGC	0.667713758
273	CCCTCTGGGTGCCAGAGT	0.667609163
1883	CCAGTGGGGTGTCCTCCC	0.667572717
1884	CAGTGGGGTGTCCTCCCC	0.667572717
1881	GGCCAGTGGGGTGTCCTC	0.667085788
1005	GCAGGTACAGGCCCTCCA	0.666773766
1882	GCCAGTGGGGTGTCCTCC	0.666545466
450	CCCTGTGGTTGCTGGTGT	0.666530826
291	CCAGCCCTAGCTGGGGTC	0.666353926
292	CAGCCCTAGCTGGGGTCC	0.666353926
296	CCTAGCTGGGGTCCCAGC	0.666353926
994	CAGCAGGTTGTGCAGGTA	0.665752534
1753	GCCTCTGGGCAGCTAGCT	0.665703672
1500	GGTACCAGGGGCAGGAGA	0.664808259
315	GGGTTCAGAAGGGGCTCT	0.664074799
1292	CCCAGGGACAGGCTGTAG	0.663756231

1627	CTGGTCACCGTAGAGCTT	0.663379701

9.11 Top 100 antisense PNA sequences for SGLT2

Serial No.	PNA sequences	Predicted scores
1034	GGAGCAGGTGGTAGGAGT	0.748956347
1474	GCTGTCAGGTACACGGGT	0.709419827
124	GCAGTTGGTCTTAGGCAT	0.699913366
1218	GGTCTGTACCGTGTCCGT	0.696024254
566	GAAGTAGAGGTAGTGCAC	0.693083382
873	CAGGTACCCACACAGGAT	0.692741791
1035	GAGCAGGTGGTAGGAGTC	0.69232843
569	GTAGAGGTAGTGCACGCC	0.691767239
390	CCATTCTGTACAGGGAGT	0.688170601
1497	CAGCCAGCCCAGTAGCAG	0.685416018
1665	GAAGTAGCCGCCCACAGT	0.682582911
1040	GGTGGTAGGAGTCGGGCC	0.681688551
1503	GCCCAGTAGCAGCACCAC	0.68073968
1500	CCAGCCCAGTAGCAGCAC	0.67781599
1501	CAGCCCAGTAGCAGCACC	0.67781599
1038	CAGGTGGTAGGAGTCGGG	0.676507716
1124	CTGCTCCCAGGTATTTGT	0.676414523
865	GTCAGCTTCAGGTACCCA	0.675790853
433	CCAGGTCCTCCCGTTCCT	0.675466049
1037	GCAGGTGGTAGGAGTCGG	0.674940144
870	CTTCAGGTACCCACACAG	0.672860719
1028	GGTGCCGGAGCAGGTGGT	0.672348281
436	GGTCCTCCCGTTCCTCCT	0.671533428
101	GGCTGTGGCTTATGGTGT	0.670191614
563	GGCGAAGTAGAGGTAGTG	0.668498235
1674	GCCCACAGTGCCTCTGTT	0.668414112
1394	GGGAGAGCACAGACAGGT	0.667144279
76	GGCTCCTCACCCCCACTT	0.666718897
437	GTCCTCCCGTTCCTCCTT	0.666709087
202	GGGCCCAGCTCGGGTCCT	0.666502354
266	GGTAAGGGGCGGAGGACT	0.665832121
1499	GCCAGCCCAGTAGCAGCA	0.664647552
1223	GTACCGTGTCCGTGTACA	0.66411049
1677	CACAGTGCCTCTGTTGGT	0.662742711

125	CAGTTGGTCTTAGGCATA	0.662710863
397	GTACAGGGAGTGAGGAGC	0.661432967
1031	GCCGGAGCAGGTGGTAGG	0.661098716
19	GGGGAGCCAGGACCTTCT	0.660802449
81	CTCACCCCCACTTCCTGT	0.659966852
40	CCCACTGCCCCTTCCCCT	0.659933539
115	GTGTCCAACGCAGTTGGT	0.659318154
127	GTTGGTCTTAGGCATAGA	0.658437691
955	CTGCACCAGTACCAGCCC	0.657781159
1400	GCACAGACAGGTAGAGGC	0.657569426
954	GCTGCACCAGTACCAGCC	0.656753908
99	GAGGCTGTGGCTTATGGT	0.656425612
564	GCGAAGTAGAGGTAGTGC	0.656406944
1446	CAGGTACTGTGGCATCGT	0.655736069
41	CCACTGCCCCTTCCCCTT	0.65564952
1668	GTAGCCGCCCACAGTGCC	0.654583323
1120	GTCGCTGCTCCCAGGTAT	0.654551159
1253	GCCCTCCTGTCACCGTGT	0.653814061
279	GGACTGCCCACCCCACCT	0.653532905
429	GCATCCAGGTCCTCCCGT	0.652488842
864	CGTCAGCTTCAGGTACCC	0.652282852
869	GCTTCAGGTACCCACACA	0.652092252
862	GGCGTCAGCTTCAGGTAC	0.651795923
42	CACTGCCCCTTCCCCTTT	0.651365501
863	GCGTCAGCTTCAGGTACC	0.651255602
747	GGAGCAGCCCACCTCCGT	0.650732724
1043	GGTAGGAGTCGGGCCGGG	0.650534263
1044	GTAGGAGTCGGGCCGGGG	0.650534263
1492	GCAAACAGCCAGCCCAGT	0.65050138
130	GGTCTTAGGCATAGAAGC	0.649987496
400	CAGGGAGTGAGGAGCCTT	0.649758913
28	GGACCTTCTCACCCCACT	0.649664862
1655	GTCCTGCCAGGAAGTAGC	0.649583476
131	GTCTTAGGCATAGAAGCC	0.649447175
284	GCCCACCCCACCTCTGCT	0.649382193
430	CATCCAGGTCCTCCCGTT	0.649232075
867	CAGCTTCAGGTACCCACA	0.649168562
906	GTGGGTCAGGCTCTTCCC	0.648835996
509	GGTGCACAGGGAGACCGT	0.648184784

1779	GGCCTTCTGGGCCCCCAT	0.648101392
1477	GTCAGGTACACGGGTGCA	0.648047355
257	CTCCTCCTGGGTAAGGGG	0.647916735
1396	GAGAGCACAGACAGGTAG	0.647575098
1398	GAGCACAGACAGGTAGAG	0.647575098
256	CCTCCTCCTGGGTAAGGG	0.647376414
35	CTCACCCCACTGCCCCTT	0.646125425
117	GTCCAACGCAGTTGGTCT	0.645892702
255	GCCTCCTCCTGGGTAAGG	0.645808841
1475	CTGTCAGGTACACGGGTG	0.64483005
1251	CAGCCCTCCTGTCACCGT	0.644321231
343	GTGCCGGGGCCTGGGGCT	0.643181249
1504	CCCAGTAGCAGCACCACG	0.642833348
515	CAGGGAGACCGTGAGGGT	0.642555495
1041	GTGGTAGGAGTCGGGCCG	0.642214647
1570	CCAGTCCCTGCCAGGCCC	0.642130565
562	TGGCGAAGTAGAGGTAGT	0.641684316
1033	CGGAGCAGGTGGTAGGAG	0.641120991
1569	GCCAGTCCCTGCCAGGCC	0.641103314
1443	GCGCAGGTACTGTGGCAT	0.64086739
861	GGGCGTCAGCTTCAGGTA	0.640195057
958	CACCAGTACCAGCCCGAG	0.639587427
1470	CCCCGCTGTCAGGTACAC	0.639437411
176	GAGCAGGGCATTGAGGTT	0.639218644
475	CCAGGCGGTGGAGGTGCT	0.638869679
950	GGTCGCTGCACCAGTACC	0.63841016
337	GGCTTGGTGCCGGGGCCT	0.638356908

9.12 Top 100 antisense PNA sequences for BRAF

Serial No.	PNA sequences	Predicted scores
400	GTTACTCCGTACCTTACT	0.756706176
1208	GCTTTCACGTTAGTTAGT	0.751599391
1216	GTTAGTTAGTGAGCCAGG	0.741283783
1548	GTGGTATTGGGTGGTGTT	0.735459623
1220	GTTAGTGAGCCAGGTAAT	0.73280165
864	GGGTAACAATAGCCAGTT	0.719614374
396	GACAGTTACTCCGTACCT	0.7168425
0	GGGTTTTTTTTTTTTTTT	0.715017905

1217	TTAGTTAGTGAGCCAGGT	0.714469864
1943	GGTACCACTGTCCTCTGT	0.714173539
1326	GTTCTATTGTGTTTATAT	0.712361309
866	GTAACAATAGCCAGTTGT	0.711294757
1	GGTTTTTTTTTTTTTTTT	0.710193565
1514	GTTAGGGCAGTCTCTGCT	0.709766934
1944	GTACCACTGTCCTCTGTT	0.709349199
939	GTACTCCTACTTCATTTT	0.707883834
1117	GTCCCGTCTACCAAGTGT	0.707284971
1545	CCTGTGGTATTGGGTGGT	0.70626504
2	GTTTTTTTTTTTTTTTTT	0.705369224
1941	CAGGTACCACTGTCCTCT	0.704680709
929	GTTTTCCTGAGTACTCCT	0.700823273
1936	CCTTGCAGGTACCACTGT	0.699980714
978	GTGTAGGTGCTGTCACAT	0.697414075
973	CTGAGGTGTAGGTGCTGT	0.696938359
937	GAGTACTCCTACTTCATT	0.695446895
675	CTATTTTTACTGTGAGGT	0.694599594
1241	GCAGGGGGGGTAGCAGAC	0.694543802
1207	GGCTTTCACGTTAGTTAG	0.693761658
1115	GAGTCCCGTCTACCAAGT	0.693518968
1934	CACCTTGCAGGTACCACT	0.693411574
398	CAGTTACTCCGTACCTTA	0.692410014
1215	CGTTAGTTAGTGAGCCAG	0.691286159
388	GGCTTTTGGACAGTTACT	0.690634781
1321	GACAGGTTCTATTGTGTT	0.688555072
1542	CTTCCTGTGGTATTGGGT	0.688015248
1209	CTTTCACGTTAGTTAGTG	0.687009614
1119	CCCGTCTACCAAGTGTTT	0.685902756
1509	CAGATGTTAGGGCAGTCT	0.68421966
1213	CACGTTAGTTAGTGAGCC	0.683686131
1744	GTTGTGTGTTGTAAGTGG	0.683038969
1238	GAGGCAGGGGGGGTAGCA	0.682942937
1240	GGCAGGGGGGGTAGCAGA	0.682942937
395	GGACAGTTACTCCGTACC	0.681959651
1120	CCGTCTACCAAGTGTTTT	0.681618737
2254	GTCCAATAGGGCCTCTAT	0.681560317
2262	GGGCCTCTATATGTTCCT	0.68145396
1027	CTTTCCCTTGTAGACTGT	0.680770122

1218	TAGTTAGTGAGCCAGGTA	0.680524129
1511	GATGTTAGGGCAGTCTCT	0.680287038
2184	CTAGCTTGCTGGTGTATT	0.67847684
1325	GGTTCTATTGTGTTTATA	0.677763133
1247	GGGGTAGCAGACAAACCT	0.675970083
1899	GTGCTTTCTTTAGACTGT	0.675458853
1025	CACTTTCCCTTGTAGACT	0.674200982
2264	GCCTCTATATGTTCCTGT	0.673134343
1538	GCCTCTTCCTGTGGTATT	0.672481902
850	GCCCTCACACCACTGGGT	0.671160575
1203	GTAAGGCTTTCACGTTAG	0.669882243
1242	CAGGGGGGGTAGCAGACA	0.669621752
97	GTTCATTTATTTTCCTTT	0.668987294
1940	GCAGGTACCACTGTCCTC	0.668230287
1249	GGTAGCAGACAAACCTGT	0.667650466
1124	CTACCAAGTGTTTTCATT	0.667079444
2266	CTCTATATGTTCCTGTGT	0.66692262
1897	CAGTGCTTTCTTTAGACT	0.665966022
750	GTAAGTAATCCATGCCCT	0.665741692
2256	CCAATAGGGCCTCTATAT	0.665654884
926	CGTGTTTTCCTGAGTACT	0.664791423
1323	CAGGTTCTATTGTGTTTA	0.664122585
1736	CGTACAAAGTTGTGTGTT	0.6639258
397	ACAGTTACTCCGTACCTT	0.663306563
977	GGTGTAGGTGCTGTCACA	0.662815899
1901	GCTTTCTTTAGACTGTCT	0.662033401
1550	GGTATTGGGTGGTGTTCA	0.660661699
966	GTAACTGCTGAGGTGTAG	0.659908729
425	GGAGACAGGTATCCTCGT	0.658777297
2172	GTTGGAGTGCATCTAGCT	0.658666315
2194	GGTGTATTCTTCATAGGC	0.658493568
780	GTGCAATATCTATAAGTT	0.658366417
927	GTGTTTTCCTGAGTACTC	0.658161128
1931	CCACACCTTGCAGGTACC	0.657988403
2195	GTGTATTCTTCATAGGCC	0.657953247
1979	CTAACGATAGGTTTTTGT	0.657696435
975	GAGGTGTAGGTGCTGTCA	0.655619037
870	CAATAGCCAGTTGTGGCT	0.655440479
1947	CCACTGTCCTCTGTTTGT	0.655013

1749	GTGTTGTAAGTGGAACAT	0.654221694
865	GGTAACAATAGCCAGTTG	0.653457024
1239	AGGCAGGGGGGGTAGCAG	0.653432234
433	GTATCCTCGTCCCACCAT	0.652808206
1629	CTGTACTACAACGCTGGT	0.652710406
462	GGTCCCTGTTGTTGATGT	0.652279741
399	AGTTACTCCGTACCTTAC	0.651026111
1948	CACTGTCCTCTGTTTGTT	0.650728981
745	GGCGTGTAAGTAATCCAT	0.650303314
934	CCTGAGTACTCCTACTTC	0.649824142
1742	AAGTTGTGTGTTGTAAGT	0.649695348
1122	GTCTACCAAGTGTTTTCA	0.649039142
100	CATTTATTTTCCTTTTGT	0.648934143
1508	CCAGATGTTAGGGCAGTC	0.648796489

9.13 Top 100 antisense PNA sequences for EGFR

Serial No.	PNA sequences	Predicted scores
523	GGTACGTGGTGGGGTTGT	0.743970098
1010	GTAGTACATATTTCCTCT	0.743915527
550	GTGGGGGGCAGGTGTCCT	0.724254027
530	GGTGGGGTTGTAGAGCAT	0.715457558
716	GGTCAGTTTCTGGCAGTT	0.712562031
878	GCACAGGGCAGGGTTGTT	0.710941576
521	CTGGTACGTGGTGGGGTT	0.710491496
524	GTACGTGGTGGGGTTGTA	0.705200023
528	GTGGTGGGGTTGTAGAGC	0.699029388
534	GGGTTGTAGAGCATGAGT	0.693371939
288	GTAGCATTTATGGAGAGT	0.692695316
787	GGCAGCTGCCCAGGTGGT	0.69103258
788	GCAGCTGCCCAGGTGGTT	0.686208239
647	GTTGTGGCAGCAGTCACT	0.685701231
881	CAGGGCAGGGTTGTTGCT	0.684032114
1165	GGACCACCTCACAGTTAT	0.683984829
494	GTATTTGCCCTCGGGGTT	0.681203226
949	CCTTCAGTCCGGTTTTAT	0.680187161
1166	GACCACCTCACAGTTATT	0.679160489
188	CAGAGGAGGAGTATGTGT	0.677678233
950	CTTCAGTCCGGTTTTATT	0.675903143

721	GTTTCTGGCAGTTCTCCT	0.674610486
327	CCTATTCCGTTACACACT	0.673850881
796	CCAGGTGGTTCTGGAAGT	0.670207885
328	CTATTCCGTTACACACTT	0.669566862
277	GTTTAATATTCGTAGCAT	0.669222087
261	GTGCAGTTTTTGAAGTGT	0.668133064
548	GAGTGGGGGCAGGTGTC	0.66782588
263	GCAGTTTTTGAAGTGTTT	0.666804001
946	GCTCCTTCAGTCCGGTTT	0.66587076
545	CATGAGTGGGGGCAGGT	0.6642009
947	CTCCTTCAGTCCGGTTTT	0.662613992
552	GGGGGCAGGTGTCCTTG	0.661591954
527	CGTGGTGGGGTTGTAGAG	0.661123056
656	GCAGTCACTGGGGGACTT	0.660760337
1009	CGTAGTACATATTTCCTC	0.659035055
196	GAGTATGTGTGAAGGAGT	0.658467308
159	GTTTTCAGAATATCCAGT	0.657985777
944	CAGCTCCTTCAGTCCGGT	0.657706994
717	GTCAGTTTCTGGCAGTTC	0.655934113
1001	GGAATTTTCGTAGTACAT	0.65579963
874	CATTGCACAGGGCAGGGT	0.655513019
144	GTGATTTCCTTTACGGTT	0.65450051
266	GTTTTTGAAGTGTTTAAT	0.653308366
1467	GGTGGCCTGTCGTCCGGT	0.65326444
146	GATTTCCTTTACGGTTTT	0.653171446
557	GCAGGTGTCCTTGCACGT	0.652975734
997	CATAGGAATTTTCGTAGT	0.65287594
275	GTGTTTAATATTCGTAGC	0.652793917
646	GGTTGTGGCAGCAGTCAC	0.650818382
1005	TTTTCGTAGTACATATTT	0.650557224
1237	GCTTGTTACTCGTGCCTT	0.650263295
553	GGGGGCAGGTGTCCTTGC	0.649500663
531	GTGGGGTTGTAGAGCATG	0.649300209
1133	CCTCTGCACATAGGTAAT	0.647158731
1162	CAAGGACCACCTCACAGT	0.646464866
879	CACAGGGCAGGGTTGTTG	0.646351799
1081	GGACATAACCAGCCACCT	0.646263987
955	GTCCGGTTTTATTTGCAT	0.645320571
745	CACCCCAGCAGCTCCCAT	0.6442103

1084	CATAACCAGCCACCTCCT	0.643140526
1134	CTCTGCACATAGGTAATT	0.642874712
1008	TCGTAGTACATATTTCCT	0.642744855
611	GCTCTCCCGGGGGCCTGT	0.642516582
520	TCTGGTACGTGGTGGGGT	0.642397718
632	GCCTGCAGCACACTGGTT	0.641304892
652	GGCAGCAGTCACTGGGGG	0.641007456
184	GATCCAGAGGAGGAGTAT	0.640984609
254	GATGGAGGTGCAGTTTTT	0.640630367
539	GTAGAGCATGAGTGGGGG	0.640358547
525	TACGTGGTGGGGTTGTAG	0.639749265
800	GTGGTTCTGGAAGTCCAT	0.639500968
941	GGGCAGCTCCTTCAGTCC	0.637775593
319	CACCAATACCTATTCCGT	0.637545606
533	GGGGTTGTAGAGCATGAG	0.635534206
508	GGTTCACATCCATCTGGT	0.635201838
634	CTGCAGCACACTGGTTGT	0.635093169
551	TGGGGGGCAGGTGTCCTT	0.634778035
555	GGGCAGGTGTCCTTGCAC	0.634611906
480	GTGGCACCAAAGCTGTAT	0.634484755
272	GAAGTGTTTAATATTCGT	0.633665015
482	GGCACCAAAGCTGTATTT	0.633155691
596	GCAGACCAGGCAGTCGCT	0.632973669
1013	GTACATATTTCCTCTGAT	0.632801289
650	GTGGCAGCAGTCACTGGG	0.632687839
405	CTGTCGGCCCCACAGGCT	0.631560446
547	TGAGTGGGGGGCAGGTGT	0.63148253
789	CAGCTGCCCAGGTGGTTC	0.631147894
969	GCATCATAGTTAGATAAG	0.631132592
990	GCTAAGGCATAGGAATTT	0.630646868
264	CAGTTTTTGAAGTGTTTA	0.629601498
561	GTGTCCTTGCACGTGGCT	0.628989962
476	GCAGGTGGCACCAAAGCT	0.627596251
991	CTAAGGCATAGGAATTTT	0.6273901
535	GGTTGTAGAGCATGAGTG	0.62721459
536	GTTGTAGAGCATGAGTGG	0.62721459
1004	ATTTTCGTAGTACATATT	0.626795549
292	CATTTATGGAGAGTGAGT	0.625589101
618	CGGGGGCCTGTGCAGCCT	0.625368802

715	TGGTCAGTTTCTGGCAGT	0.624415103

9.14 Top 100 antisense PNA sequences for HER2

Serial No.	PNA sequences	Predicted scores
197	GGGGGGCAGTACTAGGTT	0.804336501
198	GGGGGCAGTACTAGGTTT	0.799512161
4187	GGTAGGTGAGTTCCAGGT	0.77897841
4188	GTAGGTGAGTTCCAGGTT	0.77415407
4180	GTGGGCAGGTAGGTGAGT	0.762722349
2550	GTAGGAGAGGTCAGGTTT	0.760928389
707	GGTCCCAGTAATAGAGGT	0.758706392
1019	GCAGGGGTACTGTGGGGT	0.754253056
708	GTCCCAGTAATAGAGGTT	0.753882052
1190	CCAGCCCTAGTGTCAGGT	0.751949105
1022	GGGGTACTGTGGGGTCCT	0.751347686
3474	GTTGTAGGGACAGGCAGT	0.751328869
3470	GGTAGTTGTAGGGACAGG	0.751139707
470	GGTTCCTTAGGACAGGTT	0.747094435
3463	GTAGAAAGGTAGTTGTAG	0.746889506
199	GGGGCAGTACTAGGTTTC	0.742884243
3471	GTAGTTGTAGGGACAGGC	0.739048416
203	CAGTACTAGGTTTCAGGG	0.737163087
698	GTGGGTCCTGGTCCCAGT	0.73647449
3890	GGTAGCAGAGCTGGGGGT	0.735995792
3459	GTCCGTAGAAAGGTAGTT	0.735973893
201	GGCAGTACTAGGTTTCAG	0.735595515
202	GCAGTACTAGGTTTCAGG	0.735595515
4220	GGCAGCCCTGGTAGAGGT	0.734915149
1185	GGGCTCCAGCCCTAGTGT	0.734715378
208	CTAGGTTTCAGGGACAGT	0.734646649
710	CCCAGTAATAGAGGTTGT	0.733828901
3891	GTAGCAGAGCTGGGGGTT	0.731171451
1301	GGGGTACCAGATACTCCT	0.726300323
205	GTACTAGGTTTCAGGGAC	0.725289887
629	GACCCAGGTACTCTGGGT	0.722152605
1183	GAGGGCTCCAGCCCTAGT	0.720949376
3819	GTTGGTGTCTATCAGTGT	0.720779598
200	GGGCAGTACTAGGTTTCA	0.716394621

196	TGGGGGGCAGTACTAGGT	0.716189573
3461	CCGTAGAAAGGTAGTTGT	0.715920742
2472	GTCCACACAGGAGTGGGT	0.715756685
632	CCAGGTACTCTGGGTTCT	0.715285447
3993	GACAGGGGTGGTATTGTT	0.71512448
3144	GATGTATAGGTAACCTGT	0.713959582
3147	GTATAGGTAACCTGTGAT	0.713959582
1194	CCCTAGTGTCAGGTCCCC	0.713620564
1009	GTCTCAGAGGGCAGGGGT	0.71326905
1193	GCCCTAGTGTCAGGTCCC	0.712593313
643	GGGTTCTCTGCCGTAGGT	0.712087611
1049	GCTGTAGAGGGCTGGGGT	0.71201002
701	GGTCCTGGTCCCAGTAAT	0.711918453
3673	CAGCAGTCAGTGGGCAGT	0.711587653
1044	GTACCGCTGTAGAGGGCT	0.710983934
3546	CGTGTCTGTGTTGTAGGT	0.710981722
4182	GGGCAGGTAGGTGAGTTC	0.709589708
4183	GGCAGGTAGGTGAGTTCC	0.709049387
785	GTGTCAAGTACTCGGGGT	0.708905935
1052	GTAGAGGGCTGGGGTCAT	0.706856447
1565	CCCCCTTTTCCAGCAGGT	0.706446506
2468	CCAGGTCCACACAGGAGT	0.705723533
4037	CCAGGGCATAGTTGTCCT	0.705224635
1298	GCTGGGGTACCAGATACT	0.705219922
4179	GGTGGGCAGGTAGGTGAG	0.704884617
779	CCTGGGGTGTCAAGTACT	0.704831484
2728	GGGAGCCCCTGCAGTACT	0.703819915
645	GTTCTCTGCCGTAGGTGT	0.703767994
4185	CAGGTAGGTGAGTTCCAG	0.70332823
3611	GGAGGCAGGCCAGGCAGT	0.701792671
2598	GGGAGGGTCCTTATAGTG	0.701302624
2599	GGAGGGTCCTTATAGTGG	0.701302624
2600	GAGGGTCCTTATAGTGGG	0.701302624
3142	GAGATGTATAGGTAACCT	0.70019358
2930	GGTCCCAGGGCACCGTGT	0.700085223
1196	CTAGTGTCAGGTCCCCAC	0.69981245
1020	CAGGGGTACTGTGGGGTC	0.699192711
2843	GGTGGCAGGCCAGGCCCT	0.698811875
2983	GCCAGTCCACTGCCCAGT	0.696683903

2597	AGGGAGGGTCCTTATAGT	0.696662757
1017	GGGCAGGGGTACTGTGGG	0.696415324
1018	GGCAGGGGTACTGTGGGG	0.696415324
2603	GGTCCTTATAGTGGGCAC	0.696408195
1191	CAGCCCTAGTGTCAGGTC	0.695861509
3553	GTGTTGTAGGTGACCAGG	0.6956916
623	CGTCCAGACCCAGGTACT	0.695600433
1871	CCAGGTAGCTCATCCCCT	0.695542881
3547	GTGTCTGTGTTGTAGGTG	0.694821996
1023	GGGTACTGTGGGGTCCTC	0.694719769
245	GTTCTTAGACACTCCCTT	0.694254133
3033	GCCCAGCCCTTGCAGGGT	0.693447213
2984	CCAGTCCACTGCCCAGTT	0.693427135
3477	GTAGGGACAGGCAGTCAC	0.692956151
3984	GGCCCCTGTGACAGGGGT	0.692277921
3680	CAGTGGGCAGTGGCCCCT	0.692059831
3555	GTTGTAGGTGACCAGGGC	0.691919926
2477	CACAGGAGTGGGTGCAGT	0.691752594
1872	CAGGTAGCTCATCCCCTT	0.691258862
650	CTGCCGTAGGTGTCCCTT	0.690953817
3458	CGTCCGTAGAAAGGTAGT	0.69080061
471	GTTCCTTAGGACAGGTTC	0.690466517
1325	CCACCAGGTCCCCCATGT	0.689776556
298	GCTTTCAGTACCCAGGAT	0.689557983
624	GTCCAGACCCAGGTACTC	0.688970138
1195	CCTAGTGTCAGGTCCCCA	0.687671263
500	GGTTCCCCTGGAAGTGGT	0.686939689

9.15 Top 100 antisense PNA sequences for KRAS

Serial No.	PNA sequences	Predicted scores
2828	GTAGTCCTAGTTATAGAT	0.801556625
2818	GTTACCAGGAGTAGTCCT	0.794471345
2822	CCAGGAGTAGTCCTAGTT	0.791547655
2826	GAGTAGTCCTAGTTATAG	0.786159343
370	GTATACTATAGTGTCTAT	0.785960478
2825	GGAGTAGTCCTAGTTATA	0.766958449
2833	CCTAGTTATAGATTACCT	0.753903518
2823	CAGGAGTAGTCCTAGTTA	0.753317901

5239	CCTACTAGGACCATAGGT	0.752835341
369	GGTATACTATAGTGTCTA	0.751362302
4324	GTCTTTATAGTAATTTAT	0.738565339
490	ATTAGGAGTAGTACAGTT	0.737503601
4768	CTTAGGTATTCAGTTTCT	0.736055377
2824	AGGAGTAGTCCTAGTTAT	0.734931309
2831	GTCCTAGTTATAGATTAC	0.731294524
2107	GTGTACAGTAATTGTCCT	0.729313847
4976	CTTGTACTAGTATGCCTT	0.729015949
2821	ACCAGGAGTAGTCCTAGT	0.72849847
1592	GTAATTTAGTCTTTTTTT	0.727611776
4979	GTACTAGTATGCCTTAAG	0.727365573
5243	CTAGGACCATAGGTACAT	0.726601367
2907	GTCTATTCATACCAGGGT	0.726137471
4321	GGAGTCTTTATAGTAATT	0.72547596
1586	GTCTATGTAATTTAGTCT	0.722321234
4322	GAGTCTTTATAGTAATTT	0.720651619
4314	GCTATTAGGAGTCTTTAT	0.71913354
4971	TACCACTTGTACTAGTAT	0.718857914
2816	CTGTTACCAGGAGTAGTC	0.718330599
4772	GGTATTCAGTTTCTTTTT	0.716792406
2395	GGATACCATATACCCAGT	0.716558875
3655	GTTAATTTAACCAGTGTT	0.716201756
2834	CTAGTTATAGATTACCTA	0.715673764
1740	GGTAATTAACCACTACCT	0.7156128
5240	CTACTAGGACCATAGGTA	0.714605587
489	AATTAGGAGTAGTACAGT	0.713624187
367	CTGGTATACTATAGTGTC	0.713451309
3133	GTTAGGGGAATTACAAGT	0.712624756
1862	GGTACTGTGTCAAGTCTT	0.712280999
2829	TAGTCCTAGTTATAGATT	0.712080634
2353	CACTAGCAGTACCTAAGG	0.711980225
3899	GCCCTAGTCCCTCCCCAT	0.711734364
1741	GTAATTAACCACTACCTT	0.710788459
3505	CCTAGTCCAGTGATACTT	0.710232676
217	GTTATCCTATTTTTTTGT	0.709839482
491	TTAGGAGTAGTACAGTTC	0.709461699
1991	GTTTATTTGTACCCAGAT	0.708542874
1977	CTAGTTCAGGCACTGTTT	0.708542137

3900	CCCTAGTCCCTCCCCATT	0.708477596
1884	CCCTACCTAAACAGTGTT	0.70800107
2815	ACTGTTACCAGGAGTAGT	0.707084991
711	GTTTCAGTTTACACTATA	0.707065303
2350	CCACACTAGCAGTACCTA	0.706054007
3506	CTAGTCCAGTGATACTTT	0.705948657
5558	CTACCACAAGTTTATATT	0.705510209
4326	CTTTATAGTAATTTATCT	0.705086737
3901	CCTAGTCCCTCCCCATTT	0.704193577
1451	CTATAAAAGTTAGGTTCT	0.703940258
2098	CACCTTAATGTGTACAGT	0.702691556
494	GGAGTAGTACAGTTCATG	0.702620343
1646	GTAATAATTAGGTAACAT	0.702487162
366	ACTGGTATACTATAGTGT	0.702205701
2837	GTTATAGATTACCTAAGG	0.701380954
1588	CTATGTAATTTAGTCTTT	0.700939019
375	CTATAGTGTCTATGGCTT	0.700750004
1459	GTTAGGTTCTAAATTCCT	0.700744333
3902	CTAGTCCCTCCCCATTTT	0.699909558
493	AGGAGTAGTACAGTTCAT	0.697980477
365	CACTGGTATACTATAGTG	0.697352737
2355	CTAGCAGTACCTAAGGAC	0.697183334
368	TGGTATACTATAGTGTCT	0.697161109
2827	AGTAGTCCTAGTTATAGA	0.696161234
1912	GTAGTTTCACATAGCAAT	0.695514746
2895	GGATAGGGTTCTGTCTAT	0.695495159
5567	GTTTATATTCAGTCATTT	0.695494892
5246	GGACCATAGGTACATCTT	0.695242008
2820	TACCAGGAGTAGTCCTAG	0.695074853
1980	GTTCAGGCACTGTTTATT	0.694755948
5194	GCCTGTTTTGTGTCTACT	0.693508874
2013	CTATTAATTTTTAAGTAT	0.69330371
458	GTCACTGTAACTATTTTT	0.693145852
3498	CCAACAACCTAGTCCAGT	0.692463718
5562	CACAAGTTTATATTCAGT	0.692257978
2810	GTATTACTGTTACCAGGA	0.69184734
5357	CCCCAGTCCTCATGTACT	0.691714527
2400	CCATATACCCAGTGCCTT	0.69116225
2348	GACCACACTAGCAGTACC	0.690779304

2896	GATAGGGTTCTGTCTATT	0.690670818
364	TCACTGGTATACTATAGT	0.690591969
454	CATAGTCACTGTAACTAT	0.690458867
2484	GGTTTATGAGGCCAAGGT	0.689708758
4969	ATTACCACTTGTACTAGT	0.689619458
2329	GCCTTAGTAAGATATTAC	0.689289663
3478	CCTATCTAGAACCTAAGT	0.68857152
492	TAGGAGTAGTACAGTTCA	0.687796417
1875	GTCTTAACACCCTACCTA	0.687432989
5196	CTGTTTTGTGTCTACTGT	0.687297151
3294	GATGGTGTAACATAGGTT	0.687073167
875	GTAACCCAGTTAGCTCTG	0.686649685
5255	GTACATCTTCAGAGTCCT	0.686478683
4362	CCTTATAATAGTTTCCAT	0.686228038

9.16 Top 100 antisense PNA sequences for MDM2

Serial No.	PNA sequences	Predicted scores
1370	CAGTAGGTACAGACATGT	0.738660125
1283	GTTTAGTCATAATATACT	0.733325263
1356	GGTTACAGCACCATCAGT	0.717785662
1148	CTACTACCAAGTTCCTGT	0.715500079
422	GTGTTGAGTTTTCCAGTT	0.713092881
377	CTATAGTTTTTTTACAAT	0.706466267
1146	GACTACTACCAAGTTCCT	0.704657767
1364	CACCATCAGTAGGTACAG	0.704608393
1376	GTACAGACATGTTGGTAT	0.700888165
429	GTTTTCCAGTTTGGCTTT	0.698803261
1055	CTTGTACAAGGTCCTTTT	0.693853739
371	CATTCACTATAGTTTTTT	0.690822557
1366	CCATCAGTAGGTACAGAC	0.689811503
213	GTCTTGGGTTTCTTCCCT	0.688625844
49	GGGCAGGGCTTATTCCTT	0.687202166
1164	GTAGATCATGGTATATAT	0.686946473
427	GAGTTTTCCAGTTTGGCT	0.686366323
375	CACTATAGTTTTTTTACA	0.685683089
1372	GTAGGTACAGACATGTTG	0.685490882
1052	GCTCTTGTACAAGGTCCT	0.68501412
1363	GCACCATCAGTAGGTACA	0.683839927

785	GTTCACTTACACCAGCAT	0.68240641
50	GGCAGGGCTTATTCCTTT	0.682377825
1053	CTCTTGTACAAGGTCCTT	0.681757352
1361	CAGCACCATCAGTAGGTA	0.680916237
1371	AGTAGGTACAGACATGTT	0.680851016
1357	GTTACAGCACCATCAGTA	0.679015587
1349	GTGAGGTGGTTACAGCAC	0.677571491
51	GCAGGGCTTATTCCTTTT	0.677553485
458	CCCCTTTATCTTTCCCTT	0.674594723
63	CCTTTTCTTTAGCTTCTT	0.674501604
54	GGGCTTATTCCTTTTCTT	0.670364096
459	CCCTTTATCTTTCCCTTT	0.670310704
64	CTTTTCTTTAGCTTCTTT	0.670217585
34	GGTTGTCTACATACTGGG	0.670165278
638	CAGTAACTTGATATACCT	0.669087499
1058	GTACAAGGTCCTTTTGAT	0.667630612
215	CTTGGGTTTCTTCCCTTT	0.667243629
461	CTTTATCTTTCCCTTTAT	0.667219448
1375	GGTACAGACATGTTGGTA	0.666289989
55	GGCTTATTCCTTTTCTTT	0.665539756
1170	CATGGTATATATTTTCCT	0.664712047
1158	GTTCCTGTAGATCATGGT	0.664032824
38	GTCTACATACTGGGCAGG	0.663929216
1367	CATCAGTAGGTACAGACA	0.663862202
628	GCCTGATACACAGTAACT	0.66162741
206	CTTCTTTGTCTTGGGTTT	0.660004655
209	CTTTGTCTTGGGTTTCTT	0.660004655
1353	GGTGGTTACAGCACCATC	0.658917041
1351	GAGGTGGTTACAGCACCA	0.658861163
1173	GGTATATATTTTCCTGTG	0.658807595
416	CAGCTTGTGTTGAGTTTT	0.658533877
629	CCTGATACACAGTAACTT	0.658370642
35	GTTGTCTACATACTGGGC	0.658073987
994	CTAGATGAGGTAGATGGT	0.657840179
408	GCCCTCTTCAGCTTGTGT	0.657273782
1049	GAAGCTCTTGTACAAGGT	0.656510136
1076	CACTCCCACCTTCAAGGT	0.656329512
1316	GAACCAGGGTCTCTTGTT	0.654881783
1142	GATTGACTACTACCAAGT	0.65409747

409	CCCTCTTCAGCTTGTGTT	0.654017014
1360	ACAGCACCATCAGTAGGT	0.651812786
683	CTTCTTCACTAAGGCTAT	0.651399216
1347	CTGTGAGGTGGTTACAGC	0.650662029
0	CTAGGGGAAATAAGTTAG	0.650586315
424	GTTGAGTTTTCCAGTTTG	0.650430807
670	GAGAGTTCTTGTCCTTCT	0.649819268
1162	CTGTAGATCATGGTATAT	0.649320154
133	CCATTTTTAGGTCGACCT	0.649291699
1282	CGTTTAGTCATAATATAC	0.648444791
875	CACACAGAGCCAGGCTTT	0.648267893
864	CTCCCTTATTACACACAG	0.648198324
204	CTCTTCTTTGTCTTGGGT	0.647908268
866	CCCTTATTACACACAGAG	0.647857774
57	CTTATTCCTTTTCTTTAG	0.647538147
1151	CTACCAAGTTCCTGTAGA	0.646930745
1174	GTATATATTTTCCTGTGC	0.646716304
413	CTTCAGCTTGTGTTGAGT	0.646437489
1368	ATCAGTAGGTACAGACAT	0.645475609
686	CTTCACTAAGGCTATAAT	0.645092971
689	CACTAAGGCTATAATCTT	0.645092971
134	CATTTTTAGGTCGACCTT	0.64500768
1352	AGGTGGTTACAGCACCAT	0.644747743
40	CTACATACTGGGCAGGGC	0.640104391
453	GATTTCCCCTTTATCTTT	0.639803558
624	CCCTGCCTGATACACAGT	0.639738663
991	CTTCTAGATGAGGTAGAT	0.639590387
1362	AGCACCATCAGTAGGTAC	0.639532334
374	TCACTATAGTTTTTTTAC	0.639254594
691	CTAAGGCTATAATCTTCT	0.63823666
867	CCTTATTACACACAGAGC	0.636306804
868	CTTATTACACACAGAGCC	0.636306804
1092	GTGACACCTGTTCTCACT	0.634646593
1374	AGGTACAGACATGTTGGT	0.634262848
1167	GATCATGGTATATATTTT	0.634204901
926	GGCGTTTTCTTTGTCGTT	0.633718658
822	CGTCCCTGTAGATTCACT	0.633700035
366	GGAATCATTCACTATAGT	0.633447968
372	ATTCACTATAGTTTTTTT	0.633013448

428	AGTTTTCCAGTTTGGCTT	0.632830386

9.17 Top 100 antisense PNA sequences for PD-L1

Serial No.	PNA sequences	Predicted scores
330	GGTGGTGGTCTTACCACT	0.731634589
782	CTACCACATATAGGTCCT	0.726401596
626	GTCTGTAGCTACTATGCT	0.715892311
327	GGTGGTGGTGGTCTTACC	0.70332088
621	CCTCTGTCTGTAGCTACT	0.699346393
172	GGATGTGCCAGAGGTAGT	0.697655958
258	CTCATTAGTTGTTGTGTT	0.694573043
173	GATGTGCCAGAGGTAGTT	0.692831618
337	GTCTTACCACTCAGGACT	0.689554378
618	GGCCCTCTGTCTGTAGCT	0.689524859
178	GCCAGAGGTAGTTCTGGG	0.686094503
383	CGGCCTTGGGGTAGCCCT	0.685972328
180	CAGAGGTAGTTCTGGGAT	0.68151406
536	GGTACACCCCTGCATCCT	0.681337111
721	GCCAGGTCTAATTGTTTT	0.680400628
710	CAATTAGTGCAGCCAGGT	0.679426251
384	GGCCTTGGGGTAGCCCTC	0.679342033
491	CAGTAATTCGCTTGTAGT	0.678860559
176	GTGCCAGAGGTAGTTCTG	0.677774886
722	CCAGGTCTAATTGTTTTT	0.67714386
390	GGGGTAGCCCTCAGCCTG	0.676877645
328	GTGGTGGTGGTCTTACCA	0.676831257
333	GGTGGTCTTACCACTCAG	0.676037559
334	GTGGTCTTACCACTCAGG	0.676037559
716	GTGCAGCCAGGTCTAATT	0.67549363
718	GCAGCCAGGTCTAATTGT	0.67549363
331	GTGGTGGTCTTACCACTC	0.675006671
386	CCTTGGGGTAGCCCTCAG	0.673620877
719	CAGCCAGGTCTAATTGTT	0.672236862
206	CAGCTGTATGGTTTTCCT	0.672130505
771	GCTACCATACTCTACCAC	0.671281801
783	TACCACATATAGGTCCTT	0.670404206
780	CTCTACCACATATAGGTC	0.670314
414	CAGTTCATGTTCAGAGGT	0.670094029

775	CCATACTCTACCACATAT	0.669072388
620	CCCTCTGTCTGTAGCTAC	0.663923222
387	CTTGGGGTAGCCCTCAGC	0.662069908
622	CTCTGTCTGTAGCTACTA	0.661116639
779	ACTCTACCACATATAGGT	0.659068392
29	GCTTCTTTGAGTTTGTAT	0.658748973
825	CAGCAAATGCCAGTAGGT	0.655546836
507	GTCGGCACCACCATAGCT	0.65483833
336	GGTCTTACCACTCAGGAC	0.654671529
617	GGGCCCTCTGTCTGTAGC	0.65464201
106	GTCAGTGCTACACCAAGG	0.654523193
590	CCAGGGAGAGCTGGTCCT	0.654383482
17	GTGTATCACTTTGCTTCT	0.653849448
385	GCCTTGGGGTAGCCCTCA	0.652852411
19	GTATCACTTTGCTTCTTT	0.652520384
624	CTGTCTGTAGCTACTATG	0.651302534
321	GGAATTGGTGGTGGTGGT	0.651226038
504	GTAGTCGGCACCACCATA	0.65111443
175	TGTGCCAGAGGTAGTTCT	0.650960968
268	GTTGTGTTGATTCTCAGT	0.650776747
619	GCCCTCTGTCTGTAGCTA	0.650754784
591	CAGGGAGAGCTGGTCCTT	0.650099463
389	TGGGGTAGCCCTCAGCCT	0.650063726
786	CACATATAGGTCCTTGGG	0.648246588
834	CCAGTAGGTCATGAATAT	0.648233748
785	CCACATATAGGTCCTTGG	0.647706266
392	GGTAGCCCTCAGCCTGAC	0.647191997
726	GTCTAATTGTTTTTCTAC	0.646582351
179	CCAGAGGTAGTTCTGGGA	0.646375563
772	CTACCATACTCTACCACA	0.646359751
623	TCTGTCTGTAGCTACTAT	0.644541766
734	GTTTTTCTACTGGGAATT	0.642856098
644	GAACCTTCAGGTCTTCCT	0.64091601
137	CTCCCAGAATTACCAAGT	0.638002064
169	GGAGGATGTGCCAGAGGT	0.637301787
282	CAGTGTGCTGGTCACATT	0.636508736
391	GGGTAGCCCTCAGCCTGA	0.635591132
501	CTTGTAGTCGGCACCACC	0.635153392
725	GGTCTAATTGTTTTTCTA	0.634981485

763	GTCATATTGCTACCATAC	0.634907088
614	GCCGGGCCCTCTGTCTGT	0.634196965
500	GCTTGTAGTCGGCACCAC	0.634126141
712	ATTAGTGCAGCCAGGTCT	0.633400169
249	GTAGAAAATCTCATTAGT	0.633369666
471	GTATGGGGCATTGACTTT	0.632813515
112	GCTACACCAAGGCATAAT	0.632063332
235	CTCCTAAAAGTGCAGTAG	0.631683147
776	CATACTCTACCACATATA	0.630842634
141	CAGAATTACCAAGTGAGT	0.63042254
773	TACCATACTCTACCACAT	0.629784877
211	GTATGGTTTTCCTCAGGA	0.628105584
209	CTGTATGGTTTTCCTCAG	0.627593878
257	TCTCATTAGTTGTTGTGT	0.626479266
828	CAAATGCCAGTAGGTCAT	0.626407492
200	CCAATTCAGCTGTATGGT	0.626301134
584	CATTTCCCAGGGAGAGCT	0.626280122
778	TACTCTACCACATATAGG	0.625644775
380	CTTCGGCCTTGGGGTAGC	0.625600713
276	GATTCTCAGTGTGCTGGT	0.625379253
184	GGTAGTTCTGGGATGACC	0.625028808
146	TTACCAAGTGAGTCCTTT	0.62487909
612	CAGCCGGGCCCTCTGTCT	0.624704135
149	CCAAGTGAGTCCTTTCAT	0.624643476
531	GCAGCGGTACACCCCTGC	0.624568732
477	GGCATTGACTTTCACAGT	0.62433736
412	GTCAGTTCATGTTCAGAG	0.62398983

9.18 Top 100 antisense PNA sequences for VEGF

Serial No.	PNA sequences	Predicted scores
246	GTTGTGCTGTAGGAAGCT	0.670162308
338	GCACACACTCCAGGCCCT	0.660100172
278	GCTGGCCTTGGTGAGGTT	0.655232849
329	CCTCAGTGGGCACACACT	0.654428771
23	GGGAGGCTCCTTCCTCCT	0.653746367
566	GGTGGAGGTAGAGCAGCA	0.652537701
279	CTGGCCTTGGTGAGGTTT	0.651976081
569	GGAGGTAGAGCAGCAAGG	0.651354457

214	GCTCTATCTTTCTTTGGT	0.650488413
190	GGCCCACAGGGATTTTCT	0.646465091
542	GTGCAGCCTGGGACCACT	0.642883311
191	GCCCACAGGGATTTTCTT	0.641640751
561	GGCATGGTGGAGGTAGAG	0.640358547
570	GAGGTAGAGCAGCAAGGC	0.639263166
80	GTTCGTTTAACTCAAGCT	0.639002601
341	CACACTCCAGGCCCTCGT	0.638292412
84	GTTTAACTCAAGCTGCCT	0.638184918
438	GAAGATGTCCACCAGGGT	0.636875296
272	CTATGTGCTGGCCTTGGT	0.63617269
428	GGTACTCCTGGAAGATGT	0.636032242
444	GTCCACCAGGGTCTCGAT	0.635975129
193	CCACAGGGATTTTCTTGT	0.635429028
249	GTGCTGTAGGAAGCTCAT	0.634786901
209	GTCTTGCTCTATCTTTCT	0.633567685
17	CCCTGAGGGAGGCTCCTT	0.633352665
551	GGGACCACTTGGCATGGT	0.631205016
70	CTGCAAGTACGTTCGTTT	0.631134185
334	GTGGGCACACACTCCAGG	0.63006964
186	GCAAGGCCCACAGGGATT	0.629775066
562	GCATGGTGGAGGTAGAGC	0.628267256
522	CCCCCCTCCTTCTGCCAT	0.626843253
187	CAAGGCCCACAGGGATTT	0.626518298
336	GGGCACACACTCCAGGCC	0.625757644
386	GGGGCACACAGGATGGCT	0.625509275
573	GTAGAGCAGCAAGGCAAG	0.625356301
337	GGCACACACTCCAGGCCC	0.625217323
196	CAGGGATTTTCTTGTCTT	0.624288698
567	GTGGAGGTAGAGCAGCAA	0.623833946
321	GTTGGACTCCTCAGTGGG	0.622252529
269	CTCCTATGTGCTGGCCTT	0.622206917
129	GCAGGAACATTTACACGT	0.62171725
251	GCTGTAGGAAGCTCATCT	0.621361449
282	GCCTTGGTGAGGTTTGAT	0.620928614
387	GGGCACACAGGATGGCTT	0.620684935
526	CCTCCTTCTGCCATGGGT	0.620684922
241	CATTTGTTGTGCTGTAGG	0.619938928
121	GTGTTTTTGCAGGAACAT	0.619123474

73	CAAGTACGTTCGTTTAAC	0.618627011
515	GATTCTGCCCCCCTCCTT	0.618286062
123	GTTTTTGCAGGAACATTT	0.61779441
115	GAGTCTGTGTTTTTGCAG	0.617254018
76	GTACGTTCGTTTAACTCA	0.616908523
459	GATTGGATGGCAGTAGCT	0.615885314
65	CACATCTGCAAGTACGTT	0.615638834
318	GATGTTGGACTCCTCAGT	0.615564192
345	CTCCAGGCCCTCGTCATT	0.614726222
420	CTCATCAGGGTACTCCTG	0.61375269
206	CTTGTCTTGCTCTATCTT	0.613514534
611	GCAGAAAGTTCATGGTTT	0.612373519
211	CTTGCTCTATCTTTCTTT	0.612185471
357	GTCATTGCAGCAGCCCCC	0.610045
576	GAGCAGCAAGGCAAGGCT	0.609879308
441	GATGTCCACCAGGGTCTC	0.609291684
324	GGACTCCTCAGTGGGCAC	0.608416438
541	GGTGCAGCCTGGGACCAC	0.608000462
380	GCATCAGGGGCACACAGG	0.60766402
419	TCTCATCAGGGTACTCCT	0.606991922
533	CTGCCATGGGTGCAGCCT	0.60679391
572	GGTAGAGCAGCAAGGCAA	0.606155407
72	GCAAGTACGTTCGTTTAA	0.605458573
339	CACACACTCCAGGCCCTC	0.605039827
230	GTCTGCATTCACATTTGT	0.604472755
609	CAGCAGAAAGTTCATGGT	0.604209753
563	CATGGTGGAGGTAGAGCA	0.603345206
31	CCTTCCTCCTGCCCGGCT	0.603132996
221	CTTTCTTTGGTCTGCATT	0.603008525
74	AAGTACGTTCGTTTAACT	0.600525092
416	CGATCTCATCAGGGTACT	0.599405271
602	CCCAAGACAGCAGAAAGT	0.5991143
199	GGATTTTCTTGTCTTGCT	0.598735354
464	GATGGCAGTAGCTGCGCT	0.598624354
467	GGCAGTAGCTGCGCTGAT	0.598624354
330	CTCAGTGGGCACACACTC	0.598341174
117	GTCTGTGTTTTTGCAGGA	0.598053124
21	GAGGGAGGCTCCTTCCTC	0.59731822
548	CCTGGGACCACTTGGCAT	0.597186093

558	CTTGGCATGGTGGAGGTA	0.596620712
27	GGCTCCTTCCTCCTGCCC	0.596502702
540	GGGTGCAGCCTGGGACCA	0.596399596
215	CTCTATCTTTCTTTGGTC	0.595428068
440	AGATGTCCACCAGGGTCT	0.595122386
603	CCAAGACAGCAGAAAGTT	0.594830281
60	CTTGTCACATCTGCAAGT	0.59437953
422	CATCAGGGTACTCCTGGA	0.594211246
276	GTGCTGGCCTTGGTGAGG	0.59389984
332	CAGTGGGCACACACTCCA	0.593056299
417	GATCTCATCAGGGTACTC	0.592774976
425	CAGGGTACTCCTGGAAGA	0.591941236
521	GCCCCCCTCCTTCTGCCA	0.590677505
24	GGAGGCTCCTTCCTCCTG	0.587589017
